# Synthesis of Polyoxygenated Heterocycles by Diastereoselective Functionalization of a Bio-Based Chiral Aldehyde Exploiting the Passerini Reaction

**DOI:** 10.3390/molecules25143227

**Published:** 2020-07-15

**Authors:** Gabriella Vitali Forconesi, Luca Banfi, Andrea Basso, Chiara Lambruschini, Lisa Moni, Renata Riva

**Affiliations:** 1 Dipartimento di Chimica e Chimica Industriale, Università di Genova, via Dodecaneso 31, 16146 Genova, Italy; gabriella.vitali.f@gmail.com (G.V.F.); banfi@chimica.unige.it (L.B.); andrea.basso@unige.it (A.B.); chiara.lambruschini@unige.it (C.L.); lisa.moni@unige.it (L.M.); 2 Dipartimento di Farmacia, Università di Genova, viale Cembrano 4, 16147 Genova, Italy

**Keywords:** multicomponent reactions, Passerini reaction, diastereoselective reactions, lipase, desymmetrization, Michael reaction, bio-based, biomass

## Abstract

A chiral bio-based building block, prepared by the lipase-mediated desymmetrization of an erythritol derivative, was further functionalized and then submitted to stereoselective Passerini reactions, allowing the synthesis of a small library of new molecules. Thanks to the presence of different functional groups, further cyclizations were performed providing bicyclic polyoxygenated heterocycles.

## 1. Introduction

Over the last 50 years, a tremendous progress in methodologies and techniques of organic synthesis has made possible the preparation of nearly any molecule. However, not all the total syntheses reported over the years can be scaled up into cost-effective and environmentally sustainable industrial processes. According to the principles of Green Chemistry, issues such as step economy, atom economy, and operational simplicity are becoming more and more important.

Consequently, also the synthesis of libraries of compounds profits from the development of new environmentally benign, short, selective, and atom and step economic synthetic pathways.

However, the sustainability of a synthesis not only relies on the employed methodology, and the availability of the starting materials plays a very important role as well. In the last few decades, building blocks extracted from oil or other fossil materials have been the starting point for many syntheses, including polymerizations. In order to preserve these resources, in recent years, great attention within the scientific community and the governments as well has been given to the alternative exploitation of renewable sources or waste [1]. The most readily accessible biomass is undoubtedly lignocellulosic feedstock, which is a powerful precursor of many bio-based chemicals and polymers [2]. This very complex matrix is characterized by three main components: lignin, cellulose, and hemicelluloses. They can be separated and submitted to degradation processes, allowing the isolation of high added-value molecules, which can be further elaborated by synthetic methodologies or by fermentation processes.

In this context, multicomponent reactions (MCRs) can improve sustainability in both target- and diversity-oriented syntheses [3]. MCRs are still underexploited because of some limitations that hamper their full utilization. For example, they usually rely mostly on commercially available inputs. A possible solution is offered by the development of efficient pre-MCR sequences, converting simple bio-based building blocks into more complex (chiral) components. Chiral inputs may be very useful, making it possible the control of diastereoselection during the MCR and in the post-condensation transformations as well, although this goal is not easily achieved [4].

In recent years we synthesized enantiopure chiral building blocks by a chemoenzymatic [5,6,7,8,9,10] and/or organocatalytic [7,11,12] approach, often using bio-based starting materials [5,6,9,10], and used them for controlling the diastereoselectivity in different isocyanide-based MCRs (Ugi-Joullié [5,7], Passerini [6,8,9,10], or Ugi [11,12] reactions).

**Scheme 1:**
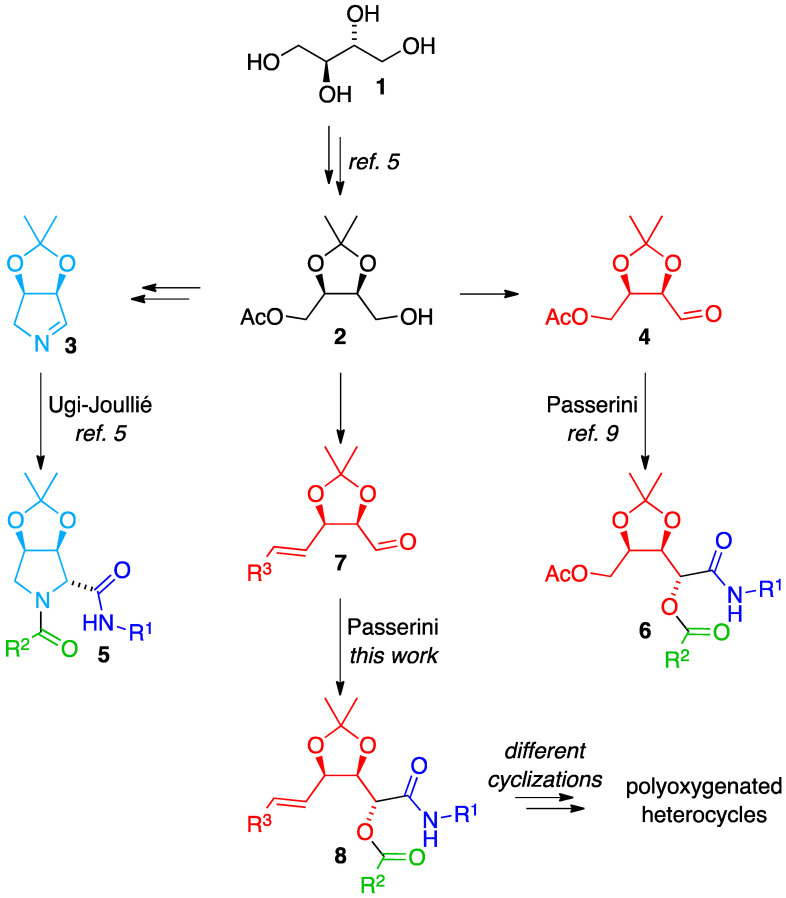
General strategy for the stereoselective functionalization of the bio-based building block **1**.

A bio-based building block readily available from lignocellulosic biomass is erythritol **1**, which is produced from glycerol [13] or from waste cooking oil [14] by means of osmophilic yeasts, such as *Yarrowia lipolytica*. This molecule is used as artificial sweetener [15], but it has found many applications in polymer chemistry, such as biodegradable polyurethanes [16,17] or polyesters [18], or in the synthesis of plant derived phthalates [19]. Erythritol, a *meso* compound, can be efficiently desymmetrized by means of Amano PS lipase [5] to give chiral monoesters, such as **2**. An appropriate functional group manipulation allowed us to obtain different chiral building blocks, namely pyrroline **3** and aldehyde **4** which were used in diastereoselective Ugi-Joullié [5,9] or a Passerini reaction [9], affording **5** and **6** respectively (Scheme 1).

Herein, we present a different elaboration of **2**, aiming to introduce a double bond. We planned to submit the resulting aldehyde **7** to a diastereoselective Passerini reaction affording **8**, followed by a series of new post-condensation transformations [20] ending up with the synthesis of polyoxygenated heterocycles.

## 2. Results and Discussion

### 2.1. Synthesis of the Bio-Based Precursor of Chiral Aldehyde to Be Used in the Passerini Reaction

**Scheme 2:**
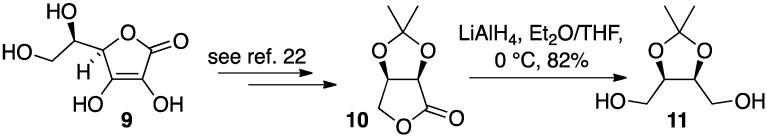
Alternative synthesis of **11**.

Chiral building block **2** (Scheme 1) can be prepared by the chemoenzymatic desymmetrization of diol **11**, which, in turn, was prepared from erythritol **1**, as previously described (Scheme 2) [5,21]. However, we also developed an efficient alternative synthesis starting from D-isoascorbic acid **9**, an antioxidant readily accessible from simple sugars. Actually, lactone **10**, prepared through a known procedure [22], can be efficiently reduced with LiAlH_4_, affording **11** in high overall yield and avoiding the difficult chromatographic purification that was needed in our previous synthesis. Even if the chirality of **9** is destroyed during the transformation into meso-**11** and reintroduced during the following lipase-mediated desymmetrization, this is compensated by the high overall yield and by the possibility to obtain both enantiomeric series, thanks to the complementary enzymatic acylation and hydrolysis [5]. Moreover, direct conversion of lactone **10** into aldehyde **4** is not feasible. The starting building block **9** is cheap and renewable.

**Scheme 3:**
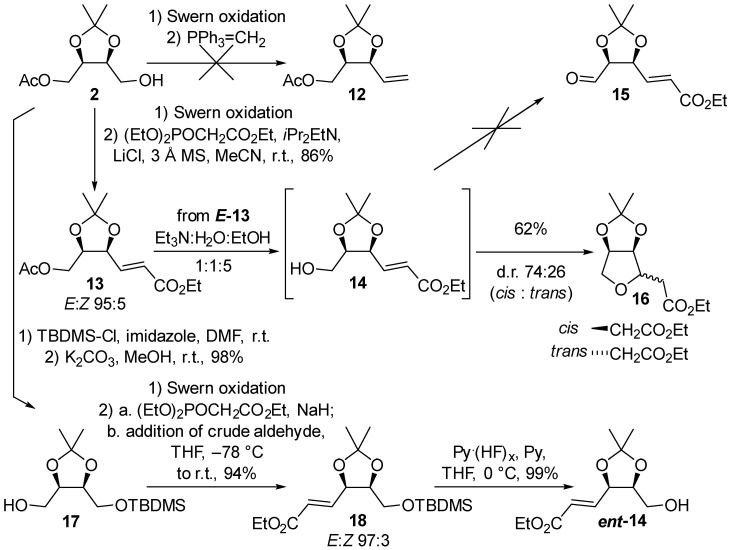
Synthesis of both enantiomers of **14**.

Initially we planned to synthesize aldehyde **7** (Scheme 1) bearing a terminal double bond (R^3^ = H) [23] and for this purpose we submitted **2** to Swern oxidation followed by Wittig olefination, but, under a variety of conditions, we never succeeded to get **12** (Scheme 3).

Then we switched to the carbethoxymethylenation which has the advantage of introducing two functional groups at once (a double bond conjugated with an ester), which could have enabled exploring different post-condensation transformations after the multicomponent reaction.

However, this reaction turned out to be not so trivial as expected. A Wittig olefination was first performed with (carbethoxymethylene)triphenylphosporane on aldehyde **4**: when the aldehyde was prepared by Swern oxidation of **2**, compound **13** was isolated in a good (73%) overall yield, but in a 41:59 *E*:*Z* ratio. Also, a partial epimerization (2%) at the stereogenic center α to the aldehyde was detected, as demonstrated by GC-MS. Switching to TEMPO/(diacetoxyiodo)benzene oxidation and performing the olefination in the presence of triethylamine, the yield increased to 89% and the diastereomeric ratio reverted to 70:30 (*E*:*Z*), but again a partial epimerization (6%) occurred.

Thus, we turned to Horner–Wadsworth–Emmons olefination. After careful optimization, the reaction in the presence of lithium chloride and Hünig’s base [24] afforded **13** in good overall yield (86%) and high stereoselectivity (95:5 *E*:*Z*), without any trace of epimerization. The minor Z isomer was readily removed by chromatography.

Then we planned to selectively hydrolyze the acetyl ester moiety on ***E*-13** to release **14**, the precursor of aldehyde **15**. Initially we tried basic conditions (1 M KOH in MeOH, Et_3_N/H_2_O/MeOH 1:1:5). In all cases, even employing a mild base like Et_3_N, which was successfully used for an analogous of **13** [9], we never identified expected **14**, isolating instead the Michael product **16** in good yield [79% (KOH) or 88% (Et_3_N)]. Moreover, we observed a total or a partial transesterification to give the methyl ester analogue of **16**. With KOH transesterification was complete, whereas with Et_3_N we obtained a Me/Et ratio of 66:34. The substitution of MeOH with EtOH (Et_3_N/H_2_O/EtOH 1:1:5) prevented transesterification but afforded a slower and complete conversion into **16** in a moderate, unoptimized, 62% overall yield. **16** was obtained as an inseparable diastereomeric mixture (d.r. 74:26, *cis*:*trans*, determined by GC-MS).

To avoid basic conditions, we switched to the enzymatic hydrolysis of the acetate. We tried several enzymes, working at pH 7 (phosphate buffer) in the presence of different cosolvents (about 15% of THF or *i*Pr_2_O) [25] and at different concentrations (0.07–0.15 M). The hydrolysis turned out to be very slow and, only using lipase from *Candida antarctica* (Novozym 435) we isolated, after 7–8 days, just a moderate amount (around 50%, depending on the concentration of **13**) of **14**. Lipase from porcine pancreas, either the commercial one or the one supported on Celite following our protocol [26], was ineffective. On the contrary, lipase from *Pseudomonas cepacia* from Amano promoted a slow hydrolysis of the acetyl but, after 6 days, only the Michael product **16** was isolated in 54% yield.

In order to suppress the Michael cyclization, we decided to substitute the acetyl with a protecting group that could be cleaved under acidic conditions, such as the *t*-butyldimethylsilyl ether. It was easily introduced through a high-yield, epimerization-free protection–deprotection sequence affording **17** [23]. Of course, this sequence produces ***ent*-14**. However, starting from the pseudoenantiomeric monobutyrate of **2**, obtained by enzymatic monohydrolysis of the corresponding dibutyrate [5], **14** could be synthesized as well. In this case, the olefination gave best results simply treating the aldehyde with the anion of triethyl phosphonoacetate, affording ***E*-18** in almost diastereomerically pure form [23].

Silyl ether removal was not as simple as expected. Under basic conditions, using *n*Bu_4_NF, TBDMS removal was rapidly followed by the intramolecular Michael reaction to give ***ent*-16**. Employing 40% HF in MeCN we also observed, as side reaction, the hydrolysis of the acetonide, although this problem was never observed on very similar substrates [5]. 25% H_2_SiF_6_ in MeCN afforded ***ent*-14** in only 30% yield, again for the partial cleavage of the acetonide [27].

Finally, we tried hydrogen fluoride pyridine complex (Olah’s reagent) in pyridine/THF [28]. After a careful optimization of the reaction conditions, with the aim of reducing the amount of Olah’s reagent, we found that lowering from 125 to 35 the equivalents, ***ent*-14** can be isolated in almost quantitative yield, even if a longer time is required (3 days vs 30 h).

### 2.2. Stereoselective Passerini Reaction

**Scheme 4:**
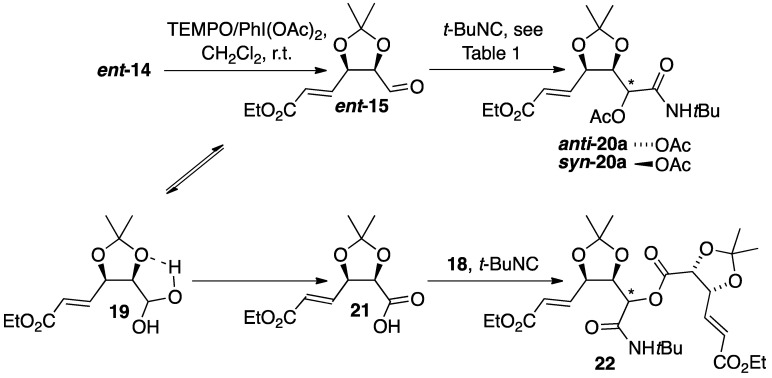
General strategy for the synthesis of Passerini products **20**. In compounds **20** and **22** * means the formation of a mixture of diastereoisomers (both *R* and *S* configuration).

***Ent*-14** is the precursor of the chiral aldehyde ***ent*-15** that we wanted to use in the diastereoselective Passerini reaction (Scheme 4). Initially, we decided to oxidize the alcohol with TEMPO/(diacetoxyiodo)benzene in methylene chloride, followed by *t*-butyl isocyanide addition, in a one-pot procedure. The third component, the carboxylic acid (AcOH), is already present, being a by-product of the oxidation. The classic Passerini conditions (entry 1, Table 1) afforded an acceptable overall yield, but only a fair diastereoselectivity (61:39) with ***anti*-20a** prevailing.

In order to increase the d.r., we added ZnBr_2_, a Lewis acid that proved to be very efficient in Passerini reactions of similar erythritol-derived aldehydes [9]. Moreover, according to our optimized procedure, we diluted the CH_2_Cl_2_ solution of the oxidation step with THF to improve the solubility of the Lewis acid (entry 2). We observed an increased stereoselectivity, but the overall yield was still unsatisfactory. The main problem seems to be the overoxidation of the intermediate aldehyde to carboxylic acid **21**, which of course can compete with acetic acid in the multicomponent reaction, affording **22**. This overoxidation is likely due to the easy formation, in the presence of humidity, of the hydrated form **19**, stabilized by an intramolecular H-bond.

Working under strictly anhydrous conditions (3 Å MS), we suppressed the overoxidation and the yield increased to some extent but diastereoselectivity slightly decreased (entry 3).

**Scheme 5:**
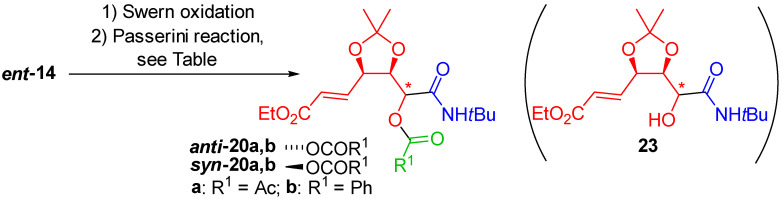
The stereoselective Passerini reaction described in Table 2. * means the formation of a mixture of diastereoisomers (both *R* and *S* configuration).

For these reasons, we decided to perform the oxidation under Swern conditions (Scheme 5), which are known to completely suppress overoxidation. Although this protocol does not permit a one-pot procedure, it has the advantage to allow the use of different carboxylic acids in the Passerini reaction. Taking care of the work-up conditions, performed under slightly acidic conditions, we were able to completely avoid the epimerization of aldehyde ***ent*-15**, which was used as such, avoiding chromatography, because of its instability over silica gel. When ***ent*-15** was submitted to a traditional Passerini reaction in methylene chloride, we isolated a mixture of ***anti*-** and ***syn*-20a** in excellent 92% overall yield from ***ent*-14** but with a poor diastereomeric ratio (59:41) (entry 1, Table 2). The use of THF further increased the yield, but not the diastereoselectivity, which was even worse (entry 2).

Moreover, the reaction turned out to be very slow (42 h vs. 1.5 h), which also favored a partial epimerization of the aldehyde.

The addition of a substoichiometric amount of ZnBr_2_ had a beneficial effect on the d.r. and an accelerating effect on the rate (4 h vs. 31 h), but the overall yield decreased (entry 3).

A problem we observed only in the ZnBr_2_ promoted Passerini is the formation of small amounts (usually less than 10%) of a diastereomeric mixture of the so called ‘truncated Passerini products’ **23**, which are not separable by chromatography from **20a**, but can be acetylated in situ maintaining the overall d.r.. The formation of ‘truncated’ products in the presence of Lewis acids is not completely unexpected and is well documented [29].

Unfortunately, the separation of ***anti*-** and ***syn*-20a** is possible, but troublesome, and therefore we decided to use benzoic acid, instead of acetic acid, because the two epimers of **20b** can be separated more easily by chromatography and by HPLC. The Passerini with no additives gave results like the one with acetic acid (entry 5). In the presence of ZnBr_2_, the rate increase was lower, compared to acetic acid (cfr. entries 3 and 6). We also studied the influence of the temperature: the reaction done at 0 °C resulted almost comparable to the one at 20 °C (entries 7 and 6), while at −30 °C the rate dramatically decreased and a lower yield and d.r. were observed (entry 8).

Based on our recent results on another chiral bio-based aldehyde [10], we also tried a modification of the Passerini reaction using zinc dicarboxylates. Using Zn(OAc)_2_, the diastereoselectivity diminished considerably and the reaction became very slow, most likely because the Zn salt is poorly soluble (entry 4). Switching to zinc benzoate, the reaction rate increased noticeably but the yield was only modest and the d.r. was not satisfactory (entry 9). The unsatisfactory overall yield is most likely because recovery of **20** is incomplete. In our previous work, to liberate the Passerini product from zinc, a strongly acidic work-up was used, but here this acidic work-up is not possible, because of the presence of the very labile isopropylidene moiety.

**Scheme 6:**
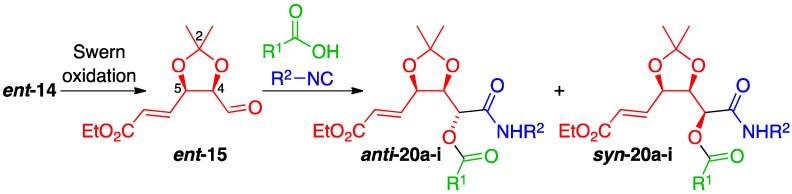
Scope of the diastereoselective Passerini reaction.

After all, since the conditions using substoichiometric ZnBr_2_ (entries 6) were the best performing ones, we decided to study the scope of the reaction (Scheme 6) as summarized in Table 3.

We used diverse isocyanides (primary, secondary, and tertiary aliphatic or aromatic) and carboxylic acids (aliphatic and aromatic). The overall yield over two steps (oxidation and Passerini) was good in all cases. The stereoselectivity ranges from moderate to excellent. We noticed that the stereoselectivity depends only marginally on the structure of the carboxylic acid. The structure of the isocyanide on the contrary influences more the stereoselectivity. In some cases we observed excellent d.r.s (>10:1), which are uncommon in intermolecular Passerini reactions of chiral aldehydes [4].

**Scheme 7:**
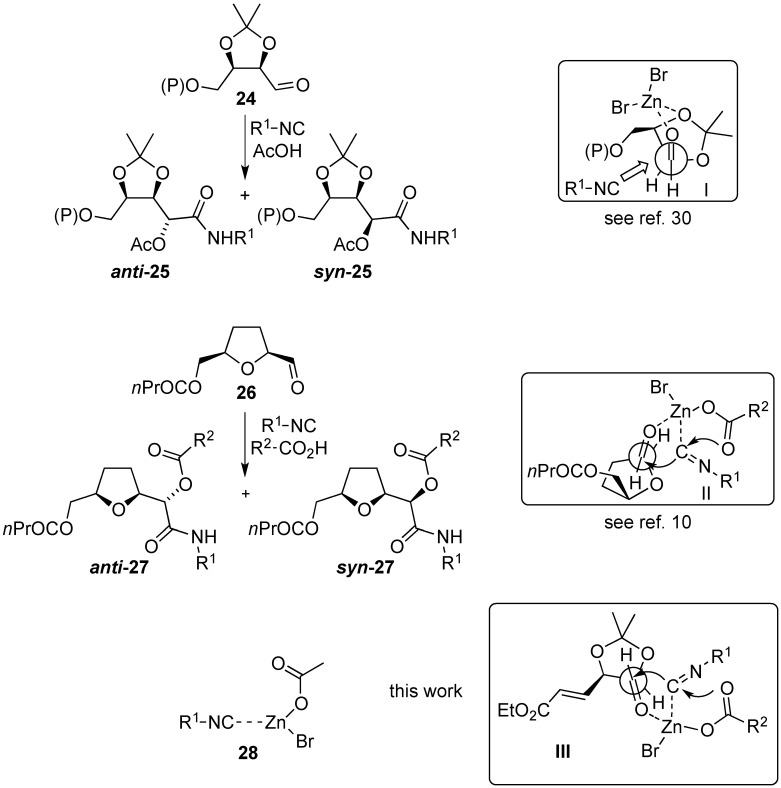
Models for rationalization of stereoselectivity in the Passerini reaction.

The role of ZnBr_2_ in promoting the stereoselectivity is not completely clear. On the previously described similar aldehyde **24** [9], we reported a model for explaining the stereoselectivity, based on the chelation of the metal by the carbonyl oxygen and the oxygen on carbon β to the aldehyde (**I**, Scheme 7) [30]. On the other hand, the outcome of chiral aldehyde **26** [10] led us to revise our rationale, also because in that case a β-chelation is clearly not possible. In the light of the observation that, performing the Passerini reaction in *i*Pr_2_O, the initially unsoluble ZnBr_2_ is partially dissolved upon addition of the isocyanide, we supposed a zinc coordination by it to give intermediate **28**. Therefore, in our previous paper, we proposed a concerted transition state, where **28** is also coordinated by the carbonyl oxygen. The formation of ***anti*-27** was then rationalized through a preferred transition state **II**.

If a similar mechanism is working on aldehyde ***ent*-14**, a possible transition state explaining the prevailing formation of ***anti*-20** would be **III**.

### 2.3. Elaboration of Passerini Products

**Scheme 8:**
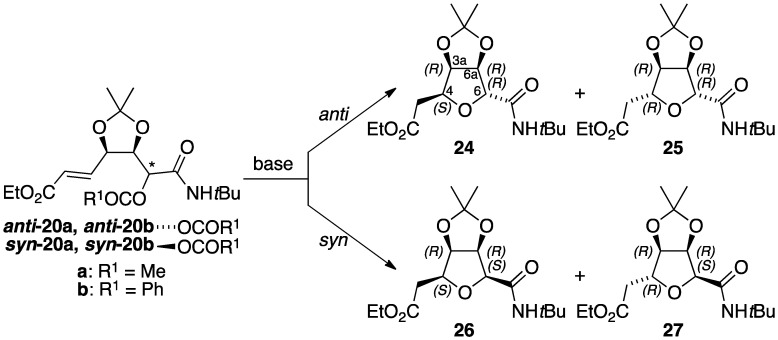
Michael cyclization of both stereoisomers of Passerini products **20a** and **20b**. * means both *R* and *S* diastereoisomers of **20a** or **20b**, which have been independently converted into **24**/**25** (***anti*-20a** or ***anti*-20b**) and **26**/**27** (***syn*-20a** or ***syn*-20b**).

The presence of additional functional groups on the Passerini products allowed us to study different post-condensation transformations thus increasing the scaffold diversity. Aware of the easy intramolecular *5-exo-trig* cyclization of **14** to give **16**, we expected that basic solvolysis of **20a** and **20b**, would afford fast cyclization of the resulting secondary alcohol. The sequence was studied on both ***anti*** and ***syn*** diastereoisomers (Scheme 8), changing the reaction conditions to optimize yield and d.r. (Table 4).

The reaction was performed independently on the separated diastereoisomers of two Passerini products, differing by the acyl substituent (R^1^ = Me, **20a**; R^1^ = Ph, **20b**), which of course afford the same tetrahydrofuran. All reactions turned out to be stereoselective, whatever the base employed. KOH was not satisfying (entries 1 and 6): despite the high reaction rate and the d.r.s which are among the best (entries 1 and 6), the isolated yield was rather poor.

Switching to sodium ethoxide [31], the reaction was slower and the d.r.s were comparable, but the yield increased significantly (entries 2 and 7). We texted the influence of the temperature as well: working a t 0 °C the reaction was slower, as expected, and we isolated the mixture of **24** and **25** in higher yield but no beneficial effect on the d.r. was observed (entry 4). As far as it concerns the starting acyl derivative, the cyclization of ***anti*-20b** affording **24** turned out to be more efficient than that of ***anti*-20a** (entries 3 and 2), while ***syn*-20a** provided **26** in higher yield but with the same d.r. as ***syn*-20b** (entries 7 and 8). Milder conditions employing Et_3_N as base in EtOH required very long time and higher temperature which negatively affects either the yield and the d.r. We also performed a one-pot, three-step sequence: Swern oxidation/Passerini reaction/cyclization. The overall yield is almost comparable, but the purification is really complex because both the Passerini and the Michael reaction are not completely stereoselective, which renders this procedure unpractical.

The relative configuration of compounds **24**–**27** was determined by NMR studies as described in the Supplementary Materials.

**Scheme 9:**
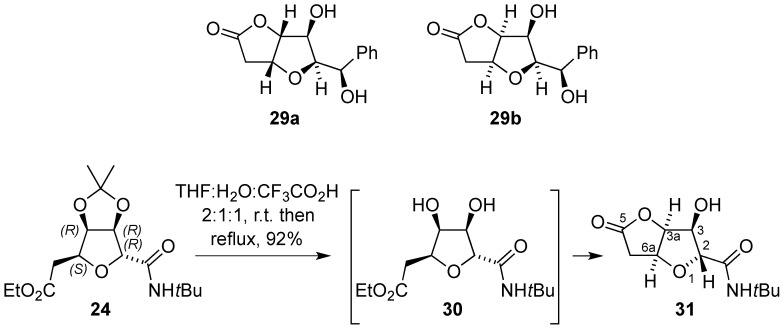
Synthesis of (+)-goniofufurone analogues.

The structure of **24** suggested the possibility to transform it into a bicyclic lactone, structurally correlated with styryllactones, such as (+)-goniofufurone **29a** [32] and its diastereoisomer **29b** [33] (Scheme 9), characterized by cytotoxic activity against several tumor cell lines [34].

For this purpose, we cleaved the isopropylidene moiety by means of trifluoroacetic acid using the previously reported conditions (MeOH:TFA:H_2_O 1.6:1:1, 87%) [5] and then further increased the yield substituting MeOH with THF (THF:TFA:H_2_O 2:1:1, 97%) although the reaction required more time (8 days vs. 3) [33] (Scheme 9).

The structure of **31** is partially rigid and, coupling MM2 calculations with NMR data, we were able to unambiguously establish the *cis* junction between the two *O*-heterocycles, and the relative *trans* relationship between the substituents on C_2_ and C_3_ (more details are reported in the Supplementary Materials). This assignment is also corroborated, especially for the relative configuration of ring junction, by comparison with the NMR spectra of **29a** and **29b**.

This confirmed the configuration of compound **24** and hence of **25**–**27** as well. Moreover, since the configuration of the stereogenic center generated during the Passerini reaction is not affected during the intramolecular Michael cyclization, we were able to assign the ***anti*** relative configuration to the prevailing stereoisomer in compounds **20**, which is also supported by spectroscopic analogies with similar compounds.

**Scheme 10:**
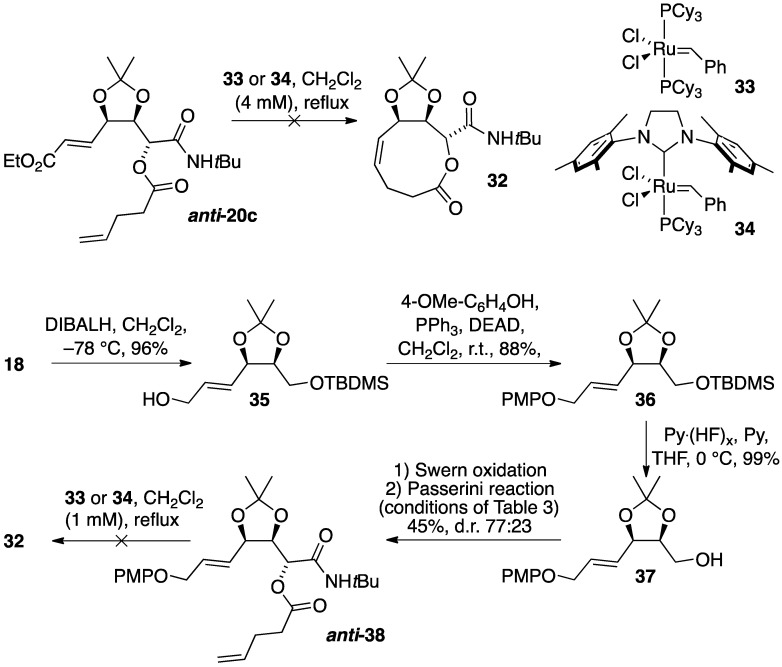
Attempted ring closing metathesis.

Finally, we decided to exploit the two double bonds of **20c** to perform a ring closing metathesis expecting to obtain **32** presumably as the *Z* stereoisomer. Although the cyclization of medium size rings is expected to be difficult, there are already several reports in the literature on the formation of 9- [35] or 10-membered lactones [36] through this strategy, but all of them used only terminal double bonds. We attempted the RCM reaction on the major ***anti*** stereoisomer and performed different experiments using either first (**33**) or second-generation Grubbs catalysts (**34**), and working under high dilution conditions (Scheme 10). Unfortunately, we only recovered unreacted starting material.

Reasoning that the presence of the ester conjugated with the double bond could be a problem, we decided to reduce ester **18** to the allylic alcohol **35** [37], which was selectively protected as anisyl ether (**36**) and finally desilylated to afford **37**, the precursor of the aldehyde to be involved in a Passerini reaction under the optimized conditions of Table 3. The MCR was only moderately stereoselective (77:13) and on ***anti*-38** we tried again the RCM with both catalysts, working in high dilution (1 mM). Again, the expected product was not obtained, and we only isolated in low yield (15%) the product of an intermolecular metathesis. One of the possible reasons of the failed cyclization is probably the presence of a substituted double bond which in many instances can hamper the RCM, though in the literature successful cyclizations involving a substituted double bond are well documented [38].

## 3. Materials and Methods

### 3.1. General Information

NMR spectra (see Supplementary Materials) were recorded on a Gemini 300 MHz instrument (Varian, Palo Alto, CA, USA) at r.t. in CDCl_3_ at 300 MHz (^1^H), and 75 MHz (^13^C), using, as internal standard, TMS (^1^H-NMR in CDCl_3_, 0.000 ppm) or the central peak of CDCl_3_ (^13^C in CDCl_3_, 77.02 ppm). Chemical shifts are reported in ppm (δ scale). Peak assignments were made with the aid of gCOSY and gHSQC experiments. In ABX system, the proton A is considered upfield and B downfield. IR spectra were recorded as solid, oil, or foamy samples, with the ATR (attenuated total reflectance) technique. TLC analyses were carried out on silica gel plates and viewed at UV (λ = 254 nm or 360 nm) and developed with Hanessian stain (dipping into a solution of (NH_4_)_4_MoO_4_·4H_2_O (21 g) and Ce(SO_4_)_2_·4H_2_O (1 g) in H_2_SO_4_ (31 mL) and H_2_O (469 mL) and warming). R_f_ values were measured after an elution of 5–7 cm. Column chromatography was done with the ‘flash’ methodology by using 220–400 mesh silica. Petroleum ether (40–60 °C) is abbreviated as PE. All reactions employing dry solvents were carried out under nitrogen. After extractions, the aqueous phases were always re-extracted two times with the appropriate organic solvent, and the organic extracts were always dried over Na_2_SO_4_ and filtered before evaporation to dryness. HRMS: samples were analyzed with a Synapt G2 QToF mass spectrometer (Waters, Milford, MA, USA). MS signals were acquired from 50 to 1200 *m/z* in either ESI positive or negative ionization mode. GC-MS were carried out using an HP-1 column (12 m long, 0.2 mm wide), electron impact at 70 eV, and a mass temperature of about 170 °C. Only *m/z* > 33 were detected. All analyses were performed (unless otherwise stated) with a constant He flow of 1.0 mL min^−1^ with initial temp. 70 °C, init. time 2 min, rate 20 °C min^−1^, final temp. 260 °C, inj. temp. 250 °C, det. temp. 280 °C. HPLC analyses were carried out on a HP-1100 system (Agilent, Santa Clara, CA, USA) equipped with a HYDRO RP column (150 × 3 mm, 4 μ) at 25 °C with flow = 0.5 mL/min and isocratic elution (CH_3_CN/H_2_O 50:50). Detection was done with UV at 220 nm; b) C6 PHENYLIC RP column (150 × 3 mm, 4 μ) at 25 °C with flow = 0.34 mL/min and gradient H_2_O/MeOH 35:65 for 20 min, then up to 30:70 until min 25. Then H_2_O:MeOH 30:70. Detection was done with UV at 220 nm.

### 3.2. Syntheses

*((4R,5S)-2,2-dimethyl-1,3-dioxolane-4,5-diyl)dimethanol* (**11**). Method A: from erythritol through a known procedure [21]. Method B: D-isoascorbic acid **9** was transformed into lactone **10**, as previously reported [22]. Then, to a suspension of LiAlH_4_ (1.756 g, 46.50 mmol) in THF dry (45 mL) at 0 °C, a solution of **10** (3.659 g, 23.15 mmol) in dry THF (32 mL) was added dropwise (40 min). The mixture was allowed to reach room temperature and stirred for 3 h. Then it was cooled to 0 °C and carefully quenched with Fieser method: deionized water (1.7 mL), NaOH (15%, 1.7 mL) and deionized water (5.1 mL) were sequentially added dropwise. The mixture was stirred until a white suspension was obtained, which was filtered through a Celite cake. The Celite was washed several times with boiling THF and the filtrate was concentrated. The residue was triturated with Et_2_O to afford **11** (3.082 g, 82% yield) as a white solid, which analytical data are agree with the reported ones [21].

*Ethyl 3-((4S,5R)-5-(acetoxymethyl)-2,2-dimethyl-1,3-dioxolan-4-yl)acrylate* (**13**). (a) Swern oxidation: to a solution of dry DMSO (87 μL, 1.22 mmol) in dry CH_2_Cl_2_ (2.9 mL), a solution of oxalyl chloride in dry CH_2_Cl_2_ (2.0 M, 515 μL) was added at −78 °C. After 10 min stirring a solution of alcohol **2** (100 mg, 0.49 mmol) in dry CH_2_Cl_2_ (2 mL) was added dropwise, and the solution was stirred for 10 min. Then Et_3_N (320 μL, 2.30 mmol) was added and the solution was stirred for 1 h at −78 °C. The reaction was quenched by addition of 5% aq. (NH_4_)H_2_PO_4_ (10 mL, added with 1 N HCl solution to reach a final pH = 4) and extracted with Et_2_O (3 × 10 mL). The combined organic layers were washed with 5% aq. NaHCO_3_ (10 mL) and brine (10 mL), dried (Na_2_SO_4_), and concentrated to afford the expected aldehyde as a yellow oil, which was used as such for the next reaction. (b) Horner-Wadsworth- Emmons reaction: to a suspension of LiCl (31 mg, 0.73 mmol) and 3 Å molecular sieves (10 mg/0.1 mmol aldehyde) in dry MeCN (5.4 mL) triethyl phosphonoacetate (165 μL, 0.73 mmol), diisopropylethylamine (85 μL, 0.49 mmol) and the crude aldehyde were added. The reaction was stirred at room temperature for 28 h, then it was filtered on a Celite cake. The solution was diluted with saturated aq. NH_4_Cl (10 mL) and extracted with Et_2_O (3 × 10 mL). The combined organic layers were washed with brine (10 mL), dried, and concentrated to afford a 95:5 *E:Z* mixture (^1^H-NMR). The residue was purified by chromatography (PE:Et_2_O 7:3) to afford ***E*-13** (108 mg, 81% yield from **11**) and ***Z*-13** (4.9 mg, 5% yield from **11**) as pale yellow oils. ***E*-13**: R_f_ = 0.22 (PE:Et_2_O 7:3). ^1^H-NMR (CDCl_3_): δ 6.85 (dd, *J* = 15.6, 5.5 Hz, 1H, CH=CHCO_2_Et), 6.15 (dd, *J* = 15.6, 1.6 Hz, 1H, CHCO_2_Et), 4.83 (ddd, *J* = 6.8, 5.5, 1.6 Hz, 1H, H-4), 4.46 (dt, *J* = 6.8, 5.3 Hz, 1H, H-5), 4.21 (q, *J* = 7.1 Hz, 2H, CH_2_CH_3_), 4.09, 3.95 (AB part of an ABX syst., *J*_AB_ = 10.0 Hz, *J*_AX_ = 4.1 Hz, *J*_BX_ = 4.9 Hz, 2H, CH_2_OAc), 2.07 (s, 3H, CH_3_CO), 1.53, 1.40 (2 s, 2 × 3H, (CH_3_)_2_C), 1.30 (t, *J* = 7.1 Hz, 3H, CH_3_CH_2_). ***Z*-13**: R_f_ = 0.38 (PE:Et_2_O 7:3). ^1^H-NMR (CDCl_3_): δ 6.29 (dd, *J* = 11.6, 7.0 Hz, 1H, CH=CHCO_2_Et), 5.94 (dd, *J* = 11.6, 1.7 Hz, 1H, CHCO_2_Et), 5.65 (td, *J* = 7.2, 1.7 Hz, 1H, *H*-4), 4.69 (td, *J* = 7.2, 3.3 Hz, 1H, H-5), 4.18 (q, *J* = 7.2 Hz, 2H, CH_2_CH_3_), 4.12 (dd, *J* = 11.7, 3.4 Hz, 1H, CHHOAc), 3.87 (dd, *J* = 11.7, 6.9 Hz, 1H, CHHOAc), 2.07 (s, 3 H, CH_3_CO), 1.53, 1.40 (2 s, 2 × 3H, (CH_3_)_2_C), 1.30 (t, *J* = 7.2 Hz, 3H, CH_3_CH_2_).

*Ethyl 2-((3aS,4S,6aR)-2,2-dimethyltetrahydrofuro[3,4-d][1,3]dioxol-4-yl)acetate* (***anti*-16**) and *Ethyl 2-((3aS,4R,6aR)-2,2-dimethyltetrahydrofuro[3,4-d][1,3]dioxol-4-yl)acetate* (***syn*-16**). A solution of **13** (50 mg, 0.18 mmol) in EtOH (1 mL) was treated with H_2_O (200 μL) and Et_3_N (200 μL) and stirred for 6 days at room temperature. The reaction was quenched with saturated aq. NH_4_Cl (10 mL) and extracted with Et_2_O (3 × 10 mL). The combined organic layers were washed with brine (10 mL), dried, and concentrated. The residue was purified by chromatography (PE: Et_2_O 6:4 to Et_2_O + 1% EtOH) to give **16** (26 mg, 62% yield, colorless oil)) as an inseparable mixture of *syn* and *anti* stereoisomers (74:26 by GC-MS). R_f_ = 0.30 (PE:Et_2_O 1:1). The individual NMR spectra were extrapolated from the NMR spectra of a 74:26 (*syn*:*anti*) mixture and are in agreement with the previously reported [31], where however the complete NMR spectra of ***syn*-16** are not available. ***Syn*-16**: ^1^H-NMR (CDCl_3_): δ 4.78 (dd, *J* = 6.1, 3.4 Hz, 1H, H-6a), 4.71 (dd, *J* = 6.1, 3.7 Hz, 1H, H-3a), 4.17 (q, *J* = 7.1 Hz, 2H, CH_2_CH_3_), 4.01 (d, *J* = 10.7 Hz, 1H, H-6), 3.86 (td, *J* = 7.2, 3.7 Hz, 1H, H-4), 3.49 (dd, *J* = 10.7, 3.6 Hz, 1H, H-6); 2.77, 2.75 (AB part of an ABX system, J_AB_ = 16.7 Hz, J_AX_ = 7.0 Hz, J_BX_ = 6.4 Hz, 2H, CH_2_CO_2_Et), 1.47, 1.33 (2 s, 2 × 3H, (CH_3_)_2_C), 1.27 (t, *J* = 7.0 Hz, 3H, CH_3_CH_2_). ^13^C-NMR (CDCl_3_): δ 171.2 (C=O), 112.2 (C-2), 81.1 (C-6a), 80.8 (C-3a), 78.3 (C-4), 72.7 (C-6), 60.8 (CH_2_CH_3_), 33.8 (CH_2_CO_2_Et), 26.0, 24.9 ((CH_3_)_2_C), 14.2 (CH_3_CH_2_). ***Anti*-16**: ^1^H-NMR: δ 4.82 (ddd, *J* = 6.0, 4.2, 1.2 Hz, 1H, H-6a), 4.57 (dd, *J* = 6.0, 1.6 Hz, 1H, H-3a), 4.45 (td, *J* = 7.1, 1.6 Hz, 1H, H-4); 4.17 (q, *J* = 7.1 Hz, 2H, CH_2_CH_3_), 3.99 (dd, *J* = 10.7, 1.5 Hz, 1 H, H-6); 3.49–3.43 (m, 1H, H-6), 2.48 (d, *J* = 7.1 Hz, 2H, CH_2_CO_2_Et), 1.52, 1.33 (2 s, 2 × 3H, (CH_3_)_2_C), 1.27 (t, *J* = 7.0 Hz, 3H, CH_3_CH_2_). ^13^C-NMR (CDCl_3_): δ 170.4 (C=O), 113.0 (C-2), 84.5 (C-3a), 81.1 (C-4), 81.0 (C-6a), 72.3 (C-6), 60.6 (CH_2_CH_3_), 36.4 (CH_2_CO_2_Et), 26.6, 25.0 ((CH_3_)_2_C), 14.2 (CH_3_CH_2_).

*((4R,5S)-5-(((tert-butyldimethylsilyl)oxy)methyl)-2,2-dimethyl-1,3-dioxolan-4-yl)methanol* (**17**). It was prepared in two steps from **2**, as previously described, as a pale yellow oil in 98% overall yield [23]. R_f_ = 0.60 (PE:Et_2_O 1:1). [α]^23^_D_ = +3.73 (c = 1.03, CHCl_3_). HRMS (ESI+): *m/z* 299.1661 (M + Na^+^). C_13_H_28_O_4_SiNa requires: 299.1649. The other analytical data agree with the reported ones.

*(E)-Ethyl 3-((4R,5S)-5-(((tert-butyldimethylsilyl)oxy)methyl)-2,2-dimethyl-1,3-dioxolan-4-yl)acrylate* (**18**). It was prepared in two steps from **17**, as previously described, as a colorless oil in 94% overall yield and a 97:3 *E*:*Z* ratio [23]. R_f_ = 0.76 (PE:Et_2_O 6:4). [α]^23^_D_ = +12.03 (c = 0.97, CHCl_3_). The analytical data agree with the reported ones.

*(E)-ethyl 3-((4R,5S)-5-(hydroxymethyl)-2,2-dimethyl-1,3-dioxolan-4-yl)acrylate* (***ent*-14**). A solution of **18** (803 mg, 2.33 mmol) was dissolved in THF/pyridine (24.6 mL, 65:35) and cooled to 0 °C. Then Olah’s reagent (hydrogen fluoride pyridine complex) (~70% HF, 2.1 mL, ~81 mmol) was added and the reaction was stirred at the same temperature for 3 days. The reaction was quenched with saturated aq. NH_4_Cl (20 mL) and extracted with Et_2_O (3 × 20 mL). The combined organic layers were washed with water (4 × 25 mL) and brine (20 mL), dried and concentrated. The residue was purified by chromatography (PE:Et_2_O 3:7) to give ***ent*-14** (532 mg, 99% yield) as a colorless oil The analytical data are in agreement with the reported ones [39]. R_f_ = 0.25 (PE/Et_2_O 3:7). [α]^24^_D_ = −33.32 (c = 1.14, CHCl_3_).

*General procedure for the synthesis of Passerini products* (**20a–i**, **38**). (a) Swern oxidation: the same procedure described above for the oxidation of **2** was followed, starting from ***ent*-14**. The crude yellow oil was thoroughly dried by azeotropic water removal and then it was directly employed in the next Passerini reaction. (b) General procedure the for the Passerini reaction under classic conditions (method A): a solution of crude aldehyde ***ent*-15** in dry CH_2_Cl_2_ (**20a**) or THF (**20a**, **20b**, **20c**, **38**) (1 M) at 20 °C was treated with the appropriate carboxylic acid (1.1 eq) and isocyanide (1.1 eq) and stirred until complete. The solution was concentrated and purified by chromatography. (c) General procedure for the Passerini reaction with ZnBr_2_ (**20a–i**, **38**) (method B): a solution of crude ***ent*-14** (2 M in THF), carboxylic acid (1.1 equiv.) and isocyanide (1.1 equiv.) were added to a solution of ZnBr_2_ (0.4 equiv.) in dry THF (1 M) at 20 °C. The resulting solution was stirred at 20 °C until complete. After quenching with saturated aq. NH_4_Cl, an extraction with ethyl acetate was performed. The combined organic layers were washed with 5% aq. NaHCO_3_, dried and concentrated. The residue was purified by chromatography.

*Ethyl (E)-3-((4R,5R)-5-((R)-1-acetoxy-2-(tert-butylamino)-2-oxoethyl)-2,2-dimethyl-1,3-dioxolan-4-yl)acrylate* (**20a**). It was prepared by method A in CH_2_Cl_2_ (overall yield *anti + syn*: 92% from ***ent*-14**, d.r. 59:41) or in THF (overall yield *anti + syn*: 97% from ***ent*-14**, d.r. 51:49), and by method B (overall yield *anti + syn*: 78% from ***ent*-14**, d.r. 80:20). Chromatography was performed with PE:CH_2_Cl_2_:Et_2_O 3:1:1. The separation of the two diastereoisomers was possible only after repeated chromatographies. ***Anti*-20a**: colorless oil. R_f_ = 0.68 (PE:Et_2_O 2:8). [α]^24^_D_ = −50.26 (c = 0.815, CHCl_3_). IR (ATR): ν_max_ 3355, 2981, 2938, 1751, 1719, 1682, 1525, 1456, 1368, 1303, 1253, 1210, 1179, 1161, 1122, 1060, 1033, 982, 938, 883, 861, 795. GC-MS: R_t_ 9.89 min: m/z 356 (M^+^ − 15, 0.2), 198 (5.1), 172 (6.8), 171 (8.6), 170 (12), 154 (7.3), 152 (8.6), 151 (7.0), 143 (11), 131 (5.3), 130 (17), 129 (6.7), 126 (14), 125 (13), 113 (6.1), 112 (30), 109 (7.9), 108 (5.5), 101 (8.1), 97 (24), 85 (15), 84 (43), 83 (5.6), 81 (10), 69 (6.0), 59 (17), 58 (43), 57 (52), 56 (5.1), 55 (10), 43 (100), 42 (6.3), 41 (23), 39 (9.9). ^1^H-NMR (CDCl_3_): δ 6.89 (dd, *J* = 15.6, 5.4 Hz, 1H, CHCH=CH), 6.12 (dd, *J* = 15.6, 1.6 Hz, 1H, O=CCH=CH), 5.82 (broad s, 1H, NH), 4.87 (ddd, *J* = 6.7, 5.4, 1.6 Hz, 1H, H-4), 4.85 (d, *J* = 7.9 Hz, 1H, CHOAc), 4.66 (dd, *J* = 7.9, 6.5 Hz, 1H, H-5), 4.19 (q, *J* = 7.1 Hz, 2H, CH_2_CH_3_), 2.10 (s, 3 H, CH_3_CO), 1.51, 1.38 (2 s, 2 × 3H, C(CH_3_)_2_), 1.32 (s, 9H, C(CH_3_)_3_), 1.27 (t, *J* = 7.1 Hz, 3H, CH_2_CH_3_). ^13^C-NMR (CDCl_3_): δ 169.4, 166.0, 165.8 (C=O), 141.3 (CHCH=C), 122.5 (CHCH=CH), 109.6 (C-2), 76.4 (C-5), 76.1 (C-4), 71.3 (CHOAc), 60.6 (CH_2_CH_3_), 51.7 (C(CH_3_)_3_), 28.6 (C(CH_3_)_3_), 27.6, 25.0 (CH_3_CCH_3_), 20.6 (CH_3_CO), 14.2 (CH_3_CH_2_). HRMS (ESI+): m/z 394.1835 (M + Na^+^). C_18_H_29_NNaO_7_ requires: 394.1836. **Syn-20a**: colorless oil. R_f_ = 0.79 (PE:Et_2_O 2:8). [α]^24^_D_ = −62.52 (c = 0.90, CHCl_3_). IR (ATR): ν_max_ 3361, 2981, 2937, 2875, 1751, 1719, 1682, 1525, 1456, 1368, 1303, 1254, 1210, 1178, 1161, 1121, 1060, 1033, 983, 938, 883, 862, 795, 661. GC-MS: R_t_ 9.79 min: m/z 356 (M^+^ − 15, 0.8), 198 (12), 172 (6.0), 171 (8.3), 170 (9.1), 154 (5.1), 152 (9.9), 151 (5.2), 143 (8.6), 198 (12), 172 (6.0), 171 (8.3), 170 (9.1), 154 (5.1), 152 (9.9), 151 (5.2), 143 (8.6), 131 (6.8), 130 (19), 129 (7.2), 126 (12), 125 (11), 113 (5.7), 112 (27), 109 (6.0), 108 (5.3), 101 (8.5), 97 (22), 85 (15), 84 (36), 83 (5.4), 81 (9.0), 69 (6.3), 59 (16), 58 (44), 57 (49), 55 (11), 43 (100), 42 (6.3), 41 (22), 39 (9.1). ^1^H-NMR (CDCl_3_): δ 6.81 (dd, *J* = 15.6, 5.6 Hz, 1 H, CH=CHCO), 6.10 (dd, *J* = 15.6, 1.5 Hz, 1H, CH=CHCO), 5.86 (broad s, 1H, NH), 4.94 (d, *J* = 5.7 Hz, 1H, CHOAc), 4.89 (ddd, *J* = 7.0, 5.6, 1.5 Hz, H-4), 4.75 (dd, *J* = 7.0, 5.7 Hz, H-5), 4.20 (q, *J* = 7.1 Hz, 2 H, CH_2_CH_3_), 2.15 (s, 3 H, CH_3_CO), 1.53, 1.39 (2 s, 2 × 3H, (CH_3_)_2_C), 1.32 (s, 9 H, C(CH_3_)_3_), 1.29 (t, *J* = 7.1 Hz, CH_3_CH_2_). ^13^C-NMR (CDCl_3_): δ 170.1, 166.2, 165.6 (C=O), 141.8 (CHCH=C), 123.5 (CHCH=CH), 109.7 (C-2), 76.7 (C-5), 75.9 (C-4), 72.8 (CHOAc), 60.6 (CH_2_CH_3_), 51.6 (C(CH_3_)_3_), 28.5 (C(CH_3_)_3_), 27.2, 25.2 (CH_3_CCH_3_), 21.0 (CH_3_CO), 14.2 (CH_3_CH_2_). HRMS (ESI+): m/z 394.1830 (M + Na^+^). C_18_H_29_NNaO_7_ requires: 394.1836.

*Ethyl (E)-3-((4R,5R)-5-((R)-1-benzoyloxy-2-(tert-butylamino)-2-oxoethyl)-2,2-dimethyl-1,3-dioxolan-4-yl)acrylate* (**20b**). It was prepared by method A in THF (overall yield *anti + syn*: 97% from ***ent*-14**, d.r. 45:55), and by method B (overall yield *anti + syn*: 59% from ***ent*-14**, d.r. 75:25). Chromatography was performed with PE:CH_2_Cl_2_:Et_2_O 3:1:1. ***Anti*-20b**: white solid. M.p. 103.5–107.1 °C (CH_2_Cl_2_). R_f_ = 0.64 (PE:Et_2_O 4:6). R_f_ = 0.25 (PE/Et_2_O 3:7), [α]^24^_D_= −53.53 (c = 0.97, CHCl_3_). IR (ATR): ν_max_ 3338, 3077, 2977, 1712, 1669, 1602, 1535, 1453, 1368, 1294, 1253, 1238, 1218, 1179, 1161, 1114, 1068, 1047, 1029, 1001, 989, 881, 819, 804, 787, 760, 709, 686, 671, 626. GC-MS: R_t_ 11.91 min: *m/z* 418 (M^+^ − 15, 2.2), 130 (5.2), 112 (8.9), 106 (7.0), 105 (100), 84 (12), 77 (16), 58 (6.7), 57 (16), 43 (7.8), 41 (7.1). ^1^H-NMR (CDCl_3_): δ 6.89 (dd, *J* = 15.6, 5.4 Hz, 1H, CHCH=CH), 6.12 (dd, *J* = 15.6, 1.6 Hz, 1H, O=CCH=CH), 5.82 (broad s, 1H, NH), 4.87 (ddd, *J* = 6.7, 5.4, 1.6 Hz, 1H, H-4), 4.85 (d, *J* = 7.9 Hz, 1H, CHOAc), 4.66 (dd, *J* = 7.9, 6.5 Hz, 1H, H-5), 4.19 (q, *J* = 7.1 Hz, 2H, CH_2_CH_3_), 2.10 (s, 3H, CH_3_CO), 1.51, 1.38 (2 s, 2 × 3H, C(CH_3_)_2_), 1.32 (s, 9H, C(CH_3_)_3_), 1.27 (t, *J* = 7.1 Hz, 3H, CH_2_CH_3_). ^13^C-NMR (CDCl_3_): δ 169.4, 166.0, 165.8 (C=O), 141.3 (CHCH=C), 122.5 (CHCH=CH), 109.6 (C-2), 76.4 (C-5), 76.1 (C-4), 71.3 (CHOAc), 60.6 (CH_2_CH_3_), 51.7 (C(CH_3_)_3_), 28.6 (C(CH_3_)_3_), 27.6, 25.0 (CH_3_CCH_3_), 20.6 (CH_3_CO), 14.2 (CH_3_CH_2_). ^1^H-NMR (CDCl_3_): δ 8.09–8.02 (m, 2 H), 7.62 (tt, *J* = 7.5, 1.4 Hz, 1H), 7.53–7.44 (m, 2H), 7.05–6.95 (m, 1H, CH=CHCO), 6.16 (dd, *J* = 15.6, 1.0 Hz, 1H, CH=CHCO), 5.90 (broad s, 1H, NH), 5.55–5.48 (m, 1H, CHOBz), 4.99–4.91 (m, 2H, H-4, H-5), 4.16–4.04 (m, 2H, CH_2_CH_3_), 1.39 (s, 6H, (CH_3_)_2_C), 1.34 (s, 9H, C(CH_3_)_3_), 1.19 (t, *J* = 7.1 Hz, CH_3_CH_2_). ^13^C-NMR (CDCl_3_): δ 166.0, 165.6, 165.1 (C=O), 142.2 (CHCH=C), 133.7, 129.9 (×2), 128.6 (×2) (aromatic CH), 128.8 (aromatic quat.), 123.0 (CHCH=CH), 109.5 (C-2), 77.0 (covered by CDCl_3_), 76.0 (C-5, C-4), 71.8 (CHOBz), 60.5 (CH_2_CH_3_), 51.7 (C(CH_3_)_3_), 28.5 (C(CH_3_)_3_), 27.2, 24.8 (CH_3_CCH_3_), 14.1 (CH_3_CH_2_). HRMS (ESI+): *m/z* 456.1995 (M + Na^+^). C_23_H_31_NNaO_7_ requires: 456.1993. ***Syn*-20b**: white solid. M.p. 99.6–103.8 (CH_2_Cl_2_). R_f_ = 0.59 (PE:Et_2_O 4:6). [α]^24^_D_= −69.80 (c =1.00, CHCl_3_). IR (ATR): ν_max_ 3362, 2981, 2965, 2937, 1730, 1713, 1688, 1603, 1586, 1543, 1495, 1454, 1381, 1364, 1317, 1296, 1256, 1213, 1181, 1161, 1134, 1099, 1060, 1039, 987, 947, 914, 885, 851, 798, 765, 716, 688, 676, 633, 610. GC-MS: R_t_ 12.23 min: *m/z* 418 (M^+^ − 15, 0.9), 171 (9.4), 130 (5.9), 112 (8.8), 106 (8.8), 105 (100), 84 (12), 77 (15), 58 (6.6), 57 (14), 43 (6.2), 41 (5.3). ^1^H-NMR (CDCl_3_): δ 8.14–8.04 (m, 2H), 7.66–7.56 (m, 1H), 7.53–7.43 (m, 2H), 6.83 (dd, *J* = 15.7, 4.9 Hz, 1H, CH=CHCO), 6.06 (dd, *J* = 15.6, 1.4 Hz, 1H, CH=CHCO), 5.96 (broad s, 1H, NH), 5.22 (d, *J* = 4.7 Hz, 1H, CHOBz), 5.01–4.88 (m, 2H, H-4, H-5), 4.03 (q, *J* = 7.1 Hz, 2H, CH_2_CH_3_), 1.59, 1.42 (2 s, 2 × 3H, (CH_3_)_2_C), 1.31 (s, 9H, C(CH_3_)_3_), 1.14 (t, *J* = 7.1 Hz, CH_3_CH_2_). ^13^C-NMR (CDCl_3_): δ 166.3, 165.6, 165.3 (C=O), 141.6 (CHCH=C), 133.7, 129.9 (×2), 128.6 (×2) (aromatic CH), 129.0 (aromatic quat.), 123.4 (CHCH=CH), 109.6 (C-2), 77.0 (partially covered by CDCl_3_), 75.8 (C-5, C-4), 73.3 (CHOBz), 60.4 (CH_2_CH_3_), 51.6 (C(CH_3_)_3_), 28.5 (C(CH_3_)_3_), 27.2, 25.0 (CH_3_CCH_3_), 14.0 (CH_3_CH_2_). HRMS (ESI+): *m/z* 456.1995 (M + Na^+^). C_23_H_31_NNaO_7_ requires: 456.1993.

*Ethyl (E)-3-((4R,5R)-5-((R)-2-(tert-butylamino)-2-oxoethyl-1-(4-pentenoyloxy))-2,2-dimethyl-1,3-dioxolan-4-yl)acrylate* (**20c**). It was prepared by method A in THF (overall yield *anti + syn*: 66% from ***ent*-14**, d.r. 47:53), and by method B (overall yield *anti* + *syn*: 53% from ***ent*-14**, d.r. 78:22). Chromatography was performed with PE:CH_2_Cl_2_:Et_2_O 3:1:1. ***Anti*-20c**: colorless oil. R_f_ = 0.70 (PE:Et_2_O 4:6). R_f_ = 0.25 (PE/Et_2_O 3:7), [α]^24^_D_= −22.22 (c = 0.99, CHCl_3_). IR (ATR): ν_max_ 3385, 2987, 2966, 2940, 1732, 1714, 1687, 1534, 1454, 1380, 1367, 1300, 1255, 1222, 1160, 1128, 1108, 1070, 1043, 1003, 984, 957, 931, 914, 881, 810, 790, 762, 744, 691, 666, 631, 603. GC-MS: R_t_ 10.75 min: m/z 396 (M^+^ − 15, 0.1), 170 (7.4), 154 (6.1), 152 (5.4), 151 (5.9), 130 (11), 129 (5.8), 126 (7.4), 125 (10), 112 (25), 97 (15), 85 (7.9), 84 (40), 83 (58), 81 (8.8), 69 (5.3), 59 (13), 58 (33), 57 (51), 56 (8.9), 55 (100), 54 (6.6), 53 (7.1), 43 (25), 42 (6.2), 41 (27), 39 (15). ^1^H-NMR (CDCl_3_): δ 6.90 (dd, *J* = 15.6, 5.6 Hz, 1H, CH=CHCO), 6.11 (dd, *J* = 15.6, 1.5 Hz, 1H, CH=CHCO), 5.81 (ddt, *J* = 17.1, 10.2, 6.1 Hz, 1H, CH=CH_2_), 5.79 (broad s, 1H, NH), 5.13–4.98 (m, 2H, CH=CH_2_), 5.03 (d, *J* = 7.2 Hz, 1H, CHOCO), 4.87 (ddd, *J* = 7.1, 5.6, 1.5 Hz, H-4), 4.72 (t, *J* = 7.1 Hz, H-5), 4.20 (q, *J* = 7.1 Hz, 2H, CH_2_CH_3_), 2.55–2.33 (m, 2H, CH_2_CH_2_), 1.51, 1.39 (2 s, 2 × 3H, (CH_3_)_2_C), 1.32 (s, 9H, C(CH_3_)_3_), 1.29 (t, *J* = 7.1 Hz, CH_3_CH_2_). ^13^C-NMR (CDCl_3_): δ 171.4, 165.9, 165.8 (C=O), 141.8 (CHCH=C), 136.3 (CH=CH_2_), 122.6 (CHCH=CH), 115.8 (CH=CH_2_), 109.5 (C-2), 76.5, 76.0 (C-5, C-4), 71.3 (CHOCO), 60.5 (CH_2_CH_3_), 51.7 (C(CH_3_)_3_), 33.0, 28.3 (CH_2_CH_2_C=C), 28.5 (C(CH_3_)_3_), 27.5, 24.9 (CH_3_CCH_3_), 14.2 (CH_3_CH_2_). HRMS (ESI+): m/z 434.2154 (M + Na^+^). C_23_H_31_NNaO_7_ requires: 434.2149. **Syn-20c**: colorless oil. R_f_ = 0.67 (PE:Et_2_O 4:6). [α]^20^_D_ = −62.20 (c = 0.54, CHCl_3_). IR (ATR): ν_max_ 3376, 2980, 2936, 1752, 1720, 1684, 1524, 1455, 1367, 1303, 1254, 1215, 1160, 1116, 1060, 1033, 984, 916, 883, 862, 796. GC-MS: R_t_ 10.66 min: m/z 396 (M^+^-15, 0.8), 311 (5.7), 254 (5.2), 198 (9.3), 197 (6.4), 181 (5.5), 172 (6.8), 171 (9.1), 170 (11), 154 (8.9), 153 (5.1), 152 (9.7), 151 (7.5), 143 (5.5), 131 (9.7), 130 (21), 129 (12), 126 (11), 125 (15), 113 (6.1), 112 (29), 108 (6.0), 101 (7.0), 97 (18), 85 (8.6), 84 (41), 83 (81), 82 (5.4), 81 (11), 69 (6.0), 59 (14), 58 (36), 57 (54), 56 (9.6), 55 (100), 54 (6.8), 53 (6.0), 43 (26), 42 (6.3), 41 (26), 39 (13). ^1^H-NMR (CDCl_3_): δ 6.81 (dd, *J* = 15.6, 5.5 Hz, 1H, CH=CHCO), 6.09 (dd, *J* = 15.6, 1.6 Hz, 1H, CH=CHCO), 5.84 (broad s, 1H, NH), 5.81 (ddt, *J* = 17.1, 10.2, 6.1 Hz, 1H, CH=CH_2_), 5.10 (dq, *J* = 17.1, 1.6 Hz, 1H, CHCHH), 5.04 (dq, *J* = 10.2, 1.5 Hz, 1H, CHCHH), 4.95 (d, *J* = 5.8 Hz, 1H, CHOCO), 4.89 (ddd, *J* = 7.1, 5.6, 1.6 Hz, H-4), 4.75 (dd, *J* = 7.0, 5.8 Hz, H-5), 4.19 (q, *J* = 7.2 Hz, 2H, CH_2_CH_3_), 2.55–2.35 (m, 2H, CH_2_CH_2_), 1.52, 1.38 (2 s, 2 × 3H, (CH_3_)_2_C), 1.31 (s, 9H, C(CH_3_)_3_), 1.28 (t, *J* = 7.2 Hz, CH_3_CH_2_). ^13^C-NMR (CDCl_3_): δ 172.2, 166.2, 165.6 (C=O), 141.9 (CHCH=C), 136.2 (CH=CH_2_), 123.5 (CHCH=CH), 116.0 (CH=CH_2_), 109.7 (C-2), 76.7, 75.9 (C-5, C-4), 72.8 (CHOCO), 60.6 (CH_2_CH_3_), 51.6 (C(CH_3_)_3_), 33.4, 28.5 (CH_2_CH_2_C=C), 28.5 (C(CH_3_)_3_), 27.2, 25.2 (CH_3_CCH_3_), 14.2 (CH_3_CH_2_). HRMS (ESI+): m/z 434.2150 (M + Na^+^). C_23_H_31_NNaO_7_ requires: 434.2149.

*Ethyl (E)-3-((4R,5R)-5-((R)-1-acetoxy-2-((2,6-dimethylphenyl)amino)-2-oxoethyl)-2,2-dimethyl-1,3-dioxolan-4-yl)acrylate* (**20d**). It was prepared by method B in THF (overall yield *anti + syn*: 51% from ***ent*-14**, d.r. 93:7). Chromatography was performed with PE:CH_2_Cl_2_:Et_2_O 3:1:1. ***Anti*-20d**: white solid. M.p. 124.8–127.1 °C (CH_2_Cl_2_). R_f_ = 0.37 (PE:CH_2_Cl_2_:Et_2_O 3:2:2). [α]^20^_D_= −48.78 (c = 0.98, CHCl_3_). IR (ATR): ν_max_ 3239, 2985, 2928, 2855, 1747, 1717, 1660, 1540, 1472, 1371, 1304, 1259, 1227, 1161, 1120, 1069, 1041, 982, 928, 880, 800, 766, 708, 685. GC-MS: R_t_ 12.64 min: m/z 419 (M^+^, 8.7), 419 (8.7), 404 (8.3), 361 (11), 219 (30), 199 (5.8), 190 (6.3), 179 (6.7), 178 (15), 177 (100), 176 (33), 172 (5.7), 160 (7.7), 148 (47), 147 (12), 143 (6.1), 126 (15), 125 (6.0), 122 (6.7), 121 (27), 120 (13), 112 (12), 109 (6.2), 106 (5.8), 105 (11), 101 (6.7), 97 (18), 91 (5.0), 85 (9.1), 84 (20), 81 (5.0), 77 (6.6), 59 (8.2), 55 (6.6), 43 (66), 39 (5.6). ^1^H-NMR (CDCl_3_): δ 7.27 (broad s, 1H, NH), 7.14–7.02 (m, 3H), 6.93 (dd, *J* = 15.6, 5.1 Hz, 1H, CHCH=CH), 6.19 (dd, *J* = 15.6, 1.2 Hz, 1H, O=CCH=CH), 4.96 (ddd, *J* = 6.3, 5.1, 1.2 Hz, 1H, H-4), 4.87 (d, *J* = 9.6 Hz, 1H, CHOAc), 4.70 (dd, *J* = 9.6, 6.3 Hz, 1H, H-5), 4.19 (q, *J* = 7.2 Hz, 2H, CH_2_CH_3_), 2.21 (s, 6H, CH_3_Ar), 2.13 (s, 3H, CH_3_CO), 1.59, 1.43 (2 s, 2 × 3H, C(CH_3_)_2_), 1.29 (t, *J* = 7.2 Hz, 3H, CH_2_CH_3_). ^13^C-NMR (CDCl_3_): δ 169.6, 165.7, 165.5 (C=O), 140.8 (CHCH=C), 135.4 (×2), 133.0 (aromatic quat.), 128.1 (×2), 127.4 (aromatic CH), 122.5 (CHCH=CH), 110.1(C-2), 76.2 (C-4), 75.9 (C-5), 71.2 (CHOAc), 60.6 (CH_2_CH_3_), 27.8, 25.1 (CH_3_CCH_3_), 20.3 (CH_3_CO), 18.3 (CH_3_Ar), 14.2 (CH_3_CH_2_). HRMS (ESI+): m/z 442.1846 (M + Na^+^). CH_29_NNaO_7_ requires: 442.1836.

*Ethyl (E)-3-((4R,5R)-5-((R)-1-acetoxy-2-(1,1-bis-(ethoxycarbonyl)-3-butenylamino)-2-oxoethyl)-2,2-dimethyl-1,3-dioxolan-4-yl)acrylate* (**20e**). It was prepared by method B in THF (overall yield *anti + syn*: 64% from ***ent*-14**, d.r. 98:2). Chromatography was performed with PE:CH_2_Cl_2_:Et_2_O 3:1:1. ***Anti*-20e**: pale yellow oil. R_f_ = 0.45 (PE:CH_2_Cl_2_:Et_2_O 3:1:1). [α]^20^_D_= −39.19 (c = 1.04, CHCl_3_). IR (ATR): ν_max_ 3410, 2984, 2925, 2855, 1740, 1720, 1695, 1505, 1466, 1370, 1305, 1259, 1213, 1178, 1160, 1096, 1060, 1014, 987, 928, 882, 857, 795, 756, 665. GC-MS: R_t_ 12.29 min: m/z 498 (M^+^ − 15, 11), 498 (11), 455 (9.6), 440 (11), 410 (5.8), 396 (5.9), 383 (6.7), 382 (35), 344 (15), 313 (7.5), 272 (5.5), 271 (19), 241 (10), 239 (8.9), 229 (7.7), 216 (7.5), 214 (10), 213 (6.3), 199 (34), 198 (13), 181 (12), 174 (13), 172 (6.0), 171 (25), 170 (27), 168 (7.1), 153 (12), 143 (20), 142 (51), 141 (9.1), 126 (7.3), 125 (17), 124 (10), 114 (5.4), 113 (8.5), 112 (58), 111 (6.0), 109 (6.6), 101 (14), 97 (37), 96 (16), 87 (6.7), 85 (17), 84 (41), 83 (7.1), 81 (7.5), 73 (5.2), 71 (5.8), 69 (11), 68 (26), 59 (12), 55 (9.4), 44 (8.6), 43 (100), 41 (14), 39 (6.9). ^1^H-NMR (CDCl_3_): δ 7.44 (broad s, 1 H, NH), 6.89 (dd, *J* = 15.6, 4.9 Hz, 1 H, CH=CHCO), 6.15 (dd, *J* = 15.6, 1.4 Hz, 1H, CH=CHCO), 5.58 (ddt, *J* = 17.8, 10.2, 7.4 Hz, 1H, CH=CH_2_), 5.17–5.05 (m, 2H, CH=CH_2_), 4.29–4.14 (m, 6H, CH_2_CH_3_), 3,07, 3.03 (AB part of an ABX syst., J_AB_ = 14.2 Hz, J_AX_ = 7.9 Hz, J_BX_ = 7.1 Hz, 2H, CH_2_CH=CH_2_), 2.09 (CH_3_CO), 1.60, 1.41 (2 s, 2 × 3H, (CH_3_)_2_C), 1.28 (t, *J* = 7.2 Hz, CH_3_CH_2_), 1.25 (t, *J* = 7.2 Hz, CH_3_CH_2_), 1.24 (t, *J* = 7.2 Hz, CH_3_CH_2_). ^13^C-NMR (CDCl_3_): δ 168.9, 167.13, 167.08, 165.8, 165.7 (C=O), 140.5 (CHCH=C), 131.0 (CH=CH_2_), 122.2 (CHCH=CH), 119.9 (CH=CH_2_), 110.1 (C-2), 76.2 (C-4), 75.8 (C-5), 70.1 (CHOAc), 66.2 (C(CO_2_Et)_2_), 62.6 (×2), 60.6 (CH_2_CH_3_), 36.8 (CH_2_CH=CH_2_), 27.7, 25.2 (CH_3_CCH_3_), 20.3 (CH_3_CO), 14.2, 13.9 (×2) (CH_3_CH_2_). HRMS (ESI+): m/z 536.2091 (M + Na^+^). C_24_H_35_NNaO_11_ requires: 536.2102.

*Ethyl (E)-3-((4R,5R)-5-((R)-1-benzoyloxy-2-((2,6-dimethylphenyl)amino)-2-oxoethyl)-2,2-dimethyl-1,3-dioxolan-4-yl)acrylate* (**20f**). It was prepared by method B in THF (overall yield *anti + syn*: 57% from ***ent*-14**, d.r. 90:10). Chromatography was performed with PE:CH_2_Cl_2_:Et_2_O 3:1:1. ***Anti*-20f**: white solid. M.p. 152.7–153.2 °C (CH_2_Cl_2_). R_f_ = 0.31 (PE:Et_2_O 4:6). [α]^20^_D_= +25.16 (c = 1.03, CHCl_34_). IR (ATR): ν_max_ 3247, 2987, 2935, 1726, 1671, 1602, 1515, 1475, 1452, 1371, 1263, 1223, 1176, 1161, 1110, 1071, 1030, 983, 880, 864, 766, 708, 682, 654, 619. ^1^H-NMR (CDCl_3_): δ 8.08–8.02 (m, 2H), 7.60 (tt, *J* = 7.5, 1.4 Hz, 1H), 7.46 (t, *J* = 7.5 Hz, 2H), 7.37 (broad s, 1H, NH), 7.14–7.02 (m, 3H), 6.96 (dd, *J* = 15.6, 5.4 Hz, 1H, CHCH=CH), 6.16 (dd, *J* = 15.6, 1.6 Hz, 1H, O=CCH=CH), 5.36 (d, *J* = 8.5 Hz, 1H, CHOBz), 5.03 (ddd, *J* = 6.4, 5.4, 1.5 Hz, 1H, H-4), 4.94 (dd, *J* = 8.5, 6.4 Hz, 1H, H-5), 3.99, 3.96 (AB part of an ABX_3_ syst., J_AB_ = 11.0 Hz, J_AX_ = J_BX_ = 7.2 Hz, 2H, CH_2_CH_3_), 2.23 (s, 6H, CH_3_Ar), 1.58, 1.46 (2 s, 2 × 3H, C(CH_3_)_2_), 1.08 (t, *J* = 7.2 Hz, 3H, CH_2_CH_3_). ^13^C-NMR (CDCl_3_): δ 165.4 (×2), 165.3 (C=O), 141.2 (CHCH=C), 135.4 (×2), 133.0, 128.6 (aromatic quat.), 133.7 (×2), 130.0 (×2), 128.5, 128.2 (×2), 127.5 (aromatic CH), 123.3 (CHCH=CH), 110.2 (C-2), 76.4 (C-4, C-5), 71.7 (CHOBz), 60.4 (CH_2_CH_3_), 27.8, 25.2 (CH_3_CCH_3_), 18.4 (CH_3_Ar), 14.0 (CH_3_CH_2_). HRMS (ESI+): m/z 504.2008 (M + Na^+^). C_27_H_31_NNaO_7_ requires: 504.1993.

*Ethyl (E)-3-((4R,5R)-5-((R)-1-benzoyloxy-2-(2-(4-benzyloxyphenyl)ethylamino)-2-oxoethyl)-2,2-dimethyl-1,3-dioxolan-4-yl)acrylate* (**20g**). It was prepared by method B in THF (overall yield *anti + syn*: 63% from ***ent*-14**, d.r. 85:15). Chromatography was performed with PE:CH_2_Cl_2_:Et_2_O 3:1:1. ***Anti*-20g**: pale yellow solid. M.p. 119.5–122.3 °C (CH_2_Cl_2_). R_f_ = 0.64 (PE:Et_2_O 4:6). [α]^24^_D_= −43.85 (c = 1.00, CHCl_3_). IR (ATR): ν_max_ 3409, 2983, 2937, 1714, 1683, 1611, 1583, 1530, 1511, 1453, 1379, 1241, 1177, 1162, 1111, 1070, 1026, 991, 878, 858, 806, 736, 711, 695. ^1^H-NMR (CDCl_3_): δ 8.02 (d, *J* = 8.2 Hz, 2H), 7.60 (t, *J* = 7.3 Hz, 1H), 7.46 (t, *J* = 7.8 Hz, 2H), 7.43–7.28 (m, 5H), 7.04 (d, *J* = 8.2 Hz, 2H), 6.96 (dd, *J* = 15.6, 5.4 Hz, 1H, CHCH=CH), 6.76 (d, *J* = 8.2 Hz, 2H), 6.16 (t, *J* = 6.0 Hz, 1H, NH), 6.12 (d, *J* = 15.6 Hz, 1H, O=CCH=CH), 5.49 (d, *J* = 5.2 Hz, 1H, CHOBz), 4.93 (s, 4H, CH_2_Ph, H-4, H-5), 4.04, 4.01 (AB part of an ABX_3_ syst., J_AB_ = 11.0 Hz, J_AX_ = J_BX_ = 7.2 Hz, 2H, CH_2_CH_3_), 3.58–3.36 (m, 2H, NHCH_2_), 2.72 (t, *J* = 6.9 Hz, 2H, NHCH_2_CH_2_), 1.37 (s, 6 H, C(CH_3_)_2_), 1.13 (t, *J* = 7.1 Hz, 3H, CH_2_CH_3_). ^13^C-NMR (CDCl_3_): δ 166.7, 165.5, 164.9 (C=O), 157.4, 137.0, 130.6, 128.6 (aromatic quat.), 141.9 (CHCH=C), 133.7, 129.9 (×2), 129.6 (×2), 128.54 (×2), 128.51 (×2), 127.9, 127.3 (×2), 114.9 (×2) (aromatic CH), 123.0 (CHCH=CH), 109.7 (C-2), 76.8, 76.0 (C-4, C-5), 71.5 (CHOBz), 69.8 (PhCH_2_O), 60.4 (CH_2_CH_3_), 40.5 (NHCH_2_), 34.3 (NHCH_2_CH_2_), 27.2, 24.8 (CH_3_CCH_3_), 14.0 (CH_3_CH_2_). HRMS (ESI+): m/z 610,2427 (M + Na^+^). C_34_H_37_NNaO_8_ requires: 610.2411.

*Ethyl (E)-3-((4R,5R)-5-(2-benzylamino-(R)-1-((2-methylbenzoyl)oxy)-2-oxoethyl)-2,2-dimethyl-1,3-dioxolan-4-yl)acrylate* (**20h**). It was prepared by method B in THF (overall yield *anti + syn*: 56% from ***ent*-14**, d.r. 88:12). Chromatography was performed with PE:CH_2_Cl_2_:Et_2_O 3:1:1. ***Anti*-20h**: white solid. M.p. 83.8–85.9 °C (CH_2_Cl_2_). R_f_ = 0.64 (PE:Et_2_O 4:6). [α]^24^_D_= −33.33 (c = 1.01, CHCl_3_). IR (ATR): ν_max_ 3331, 2984, 2931, 1723, 1664, 1601, 1551, 1494, 1455, 1434, 1387, 1367, 1307, 1235, 1208, 1173, 1157, 1141, 1071, 1029, 972, 922, 880, 858, 802, 737, 696, 668, 639. ^1^H-NMR (CDCl_3_): δ 7.96 (dd, *J* = 8.1, 1.5 Hz, 1H), 7.43 (td, *J* = 7.5, 1.2 Hz, 1H), 7.35–7.21 (m, 7H), 6.93 (dd, *J* = 15.6, 5.4 Hz, 1H, CHCH=CH), 6.46 (t, *J* = 6.0 Hz, 1 H, NH), 6.12 (dd, *J* = 15.6, 1.3 Hz, 1 H, O=CCH=CH), 5.38 (d, *J* = 7.0 Hz, 1H, CHOCO), 4.98–4.87 (m, 2H, H-4, H-5), 4.61 (dd, *J* = 15.1, 6.5 Hz, 1H, CHHPh), 4.33 (dd, *J* = 15.1, 5.1 Hz, 1H, CHHPh), 4.05, 4.02 (AB part of an ABX_3_ syst., J_AB_ = 11.0 Hz, J_AX_ = J_BX_ = 7.1 Hz, 2H, CH_2_CH_3_), 2.57 (s, 3H, CH_3_Ar), 1.46, 1.41 (2 s, 2 × 3H, C(CH_3_)_2_), 1.13 (t, *J* = 7.1 Hz, 3H, CH_2_CH_3_). ^13^C-NMR (CDCl_3_): δ 167.2, 165.41, 165.38 (C=O), 141.5 (CHCH=C), 141.3, 137.5, 127.6 (aromatic quat.), 132.7, 131.8, 130.8, 128.6 (×2), 127.5 (×2), 127.4, 125.8 (aromatic CH), 123.2 (CHCH=CH), 109.9 (C-2), 76.7, 76.1 (C-4, C-5), 71.3 (CHOCO), 60.4 (CH_2_CH_3_), 43.4 (NHCH_2_), 27.3, 24.9 (CH_3_CCH_3_), 21.7 (CH_3_Ar), 14.0 (CH_3_CH_2_). HRMS (ESI+): m/z 504.1990 (M + Na^+^). C_27_H_31_NNaO_7_ requires: 504.1993.

*Ethyl (E)-3-((4R,5R)-5-(2-cyclohexylamino-(R)-1-((2-methoxyacetoxy)-2-oxoethyl)-2,2-dimethyl-1,3-dioxolan-4-yl)acrylate* (**20i**). It was prepared by method B in THF (overall yield *anti + syn*: 64% from ***ent*-14**, d.r. 84:16). Chromatography was performed with PE:CH_2_Cl_2_:Et_2_O 3:2:2. ***Anti*-20i**: white solid. M.p. 146.2–148.7 °C (CH_2_Cl_2_). R_f_ = 0.20 (PE:CH_2_Cl_2_:Et_2_O 3:2:2). [α]^24^_D_= −43.13 (c = 1.03, CHCl_3_). IR (ATR): ν_max_ 3314, 2989, 2938, 2856, 1758, 1716, 1661, 1550, 1452, 1420, 1384, 1356, 1303, 1259, 1245, 1225, 1183, 1161, 1122, 1096, 1077, 1031, 978, 929, 889, 879, 853, 804, 760, 734, 720, 694, 665, 645. ^1^H-NMR (CDCl_3_): δ 6.87 (dd, *J* = 15.6, 5.4 Hz, 1H, CHCH=CH), 6.12 (dd, *J* = 15.6, 1.6 Hz, 1H, O=CCH=CH), 5.92 (t, *J* = 8.4 Hz, 1H, NH), 4.98 (d, *J* = 8.1 Hz, 1H, CHOCO), 4.89 (ddd, *J* = 6.6, 5.4, 1.6 Hz, H-4), 4.69 (dd, *J* = 8.1, 6.6 Hz, H-5), 4.18 (q, *J* = 7.1 Hz, 2H, CH_2_CH_3_), 4.13, 4.07 (AB syst., *J* = 16.8 Hz, CH_2_OMe), 3.84–3.69 (m, 1H, CHNH), 3.44 (s, 3H, CH_3_O), 1.94–1.82 (m, 2H), 1.75–1.54 (m, 4H), 1.52, 1.39 (2 s, 2 × 3H, C(CH_3_)_2_), 1.40–1.27 (m, 1H), 1.28 (t, *J* = 7.2 Hz, 3H, CH_2_CH_3_), 1.25–1.05 (m, 3H). ^13^C-NMR (CDCl_3_): δ 168.9, 165.7, 165.4 (C=O), 141.0 (CHCH=C), 122.6 (CHCH=CH), 109.7 (C-2), 76.1, 76.0 (C-4, C-5), 71.0 (CHOCO), 69.3 (CH_3_OCH_2_), 60.6 (CH_2_CH_3_), 59.4 (OCH_3_), 48.3 (NHCH), 32.7, 32.5, 25.4, 24.5 (×2) (CH_2_ of cyclohexyl), 27.5, 24.9 (CH_3_CCH_3_), 14.1 (CH_3_CH_2_). HRMS (ESI+): m/z 450.2101 (M + Na^+^). C_21_H_33_NNaO_8_ requires: 450.2098.

*(R)-2-(tert-Butylamino)-1-((4R,5R)-5-((E)-3-(4-methoxyphenoxy)prop-1-en-1-yl)-2,2-dimethyl-1,3-dioxolan-4-yl)-2-oxoethyl pent-4-enoate* (**38**). It was prepared by method A in THF (overall yield *anti + syn*: 74% from **37**, d.r. 62:38) and by method B (overall yield *anti + syn*: 45% from **37**, d.r. 77:23). Chromatography was performed with PE:CH_2_Cl_2_:Et_2_O 3:1:1. ***Anti*-38**: colorless oil6. R_f_ = 0.47 (PE:Et_2_O 1:1). [α]^20^_D_= −22.70 (c = 0.45, CHCl_3_). IR (ATR): ν_max_ 3391, 2977, 2935, 1745, 1689, 1507, 1455, 1367, 1216, 1163, 1107, 1063, 1035, 973, 916, 877, 824, 798, 744, 711. GC-MS: R_t_ 13.03 min: m/z 475 (M^+^, 5.1), 475 (5.1), 353 (21), 352 (100), 270 (13), 252 (11), 238 (5.8), 214 (14), 196 (9.2), 165 (5.1), 156 (7.9), 144 (8.8), 138 (14), 125 (8.1), 124 (54), 123 (20), 113 (5.5), 111 (33), 109 (11), 95 (13), 88 (11), 83 (31), 81 (7.1), 69 (8.9), 59 (7.9), 58 (17), 57 (32), 55 (54), 53 (13), 43 (13), 41 (15). ^1^H-NMR (CDCl_3_): δ 6.82 (s, 4 H, aromatic H), 6.02 (dtd, *J* = 15.6, 4.8, 0.6 Hz, 1H, CH-CH_2_OPMP), 5.91 (ddt, *J* = 15.6, 6.6, 1.3 Hz, 1H, CH=CHCH_2_OPMP), 5.86 (s, 1H, NH), 5.77 (ddt, *J* = 17.1, 10.2, 6.1 Hz, 1H, CH=CH_2_), 5.06–4.95 (m, 2H, CH=CH_2_), 4.99 (d, *J* = 7.8 Hz, 1H, CHOCO), 4.77 (t, *J* = 6.6 Hz, 1H, H-5), 4.56 (dd, *J* = 7.6, 6.4 Hz, 1H, H-4), 4.45 (d, *J* = 4.7 Hz, 2H, CH_2_OPMP), 3.77 (OCH_3_), 2.56–2.30 (m, 4H, CH_2_CH_2_CO), 1.50, 1.38 (2 s, 2 × 3H, (CH_3_)_2_C), 1.33 (s, 9H, (CH_3_)_3_C). ^13^C-NMR (CDCl_3_): δ 171.5, 166.2 (C=O), 154.0, 152.6 (aromatic quat.), 136.4 (CH=CH_2_), 129.3, 127.0 (CH=CH), 115.8 (CH=CH_2_), 115.5, 114.7 (aromatic CH), 109.1 (C-2), 77.5 (C-5), 76.4 (C-4), 71.5 (CHOCO), 68.0 (CH_2_OPMP), 55.7 (OCH_3_), 51.6 (C(CH_3_)_3_), 33.2 (CH_2_), 28.6 (C(CH_3_)_3_), 28.4 (CH_2_), 27.6, 25.1 ((CH_3_)_2_C). HRMS (ESI+): m/z 498,2443 (M + Na^+^). C_26_H_37_NNaO_7_ requires: 498,2462.

*Ethyl 2-((3aR,4S,6R,6aR)-6-(tert-butylcarbamoyl)-2,2-dimethyltetrahydrofuro[3,4-d][1,3]dioxol-4-yl)acetate* (**24**) and *Ethyl 2-((3aR,4R,6R,6aR)-6-(tert-butylcarbamoyl)-2,2-dimethyltetrahydrofuro[3,4-d][1,3]dioxol-4-yl)acetate* (**25**). A solution of sodium ethoxide (1.6 mL, 0.1 M, prepared in situ by addition of sodium to EtOH) was added to a solution of **anti-20a** (100 mg, 0,23 mmol) and the reaction was stirred at room temperature for 5 h. The mixture was poured into saturated aq. NH_4_Cl (10 mL) and extracted with CH_2_Cl_2_. The organic layers were washed with brine, dried, and concentrated. Chromatography with PE:Et_2_O afforded **24** (45 mg, 41%) and a mixture of **24** and **25** (18 mg, 17% yield), which was purified again to afford an analytical sample of **25**. The d.r. of the reaction is 90:10). The same reaction was performed under the same conditions starting from **anti-20b** and afforded the same product with an overall 73% and a 89:11 d.r. **24**: white solid. M.p. 61.1–62.4 °C (PE/Et_2_O)). R_f_ = 0.48 (PE:Et_2_O 1:1). [α]^21^_D_= −2.94 (c = 1.00, CHCl_3_). IR (ATR): ν_max_ 3389, 2988, 2964, 2936, 1729, 1683, 1524, 1480, 1457, 1403, 1395, 1375, 1364, 1308, 1280, 1268, 1251, 1237, 1205, 1179, 1159, 1106, 1072, 1057, 1038, 1024, 992, 973, 927, 897, 866, 839, 825, 809, 763, 698, 648, 616. GC-MS: R_t_ 9.14 min: m/z 314 (M^+^ − 15, 0.8), 226 (14), 186 (18), 185 (12), 184 (100), 172 (17), 171 (30), 170 (16), 155 (20), 154 (5.4), 143 (8.5), 129 (9.3), 128 (26), 127 (14), 126 (27), 125 (11), 109 (5.1), 101 (27), 100 (6.3), 99 (11), 98 (14), 97 (18), 88 (18), 87 (30), 85 (41), 84 (17), 83 (7.8), 82 (7.0), 81 (37), 74 (7.0), 73 (7.7), 71 (11), 70 (8.2), 69 (8.6), 59 (18), 58 (33), 57 (79), 56 (7.1), 55 (15), 43 (39), 42 (9.0), 41 (32), 39 (7.0). ^1^H-NMR (CDCl_3_): δ 6.72 (s, 1H, NH), 5.18 (dd, *J* = 6.0, 0.3 Hz, 1H, H-6a), 4.63 (dd, *J* = 6.0, 3.6 Hz, 1H, H-3a), 4.32 (s, 1H, H-6), 4.21, 4.18 (AB part of an ABX_3_ syst., J_AB_ = 10.5 Hz, J_AX_ = J_BX_ = 7.2 Hz, 2H, CH_2_CH_3_), 4.08 (dtd, *J* = 9.0, 3.8, 0.3 Hz, 1H, H-4), 2.81, 2.70 (AB part of an ABX syst., J_AB_ = 17.4 Hz, J_AX_ = 3.8 Hz, J_BX_ = 9.0 Hz., 2 H, CH_2_CO_2_Et), 1.47, 1.32 (2 s, 2 × 3H, C(CH_3_)_2_), 1.37 (s, 9H, C(CH_3_)_3_), 1.29 (t, *J* = 7.2 Hz, 3H, CH_2_CH_3_). ^13^C-NMR (CDCl_3_): δ 171.1, 167.9 (C=O), 112.7 (C-2), 83.9 (C-6), 83.6 (C-6a), 80.7 (C-3a), 77.6 (C-4), 60.8 (CH_2_CH_3_), 51.0 (C(CH_3_)_3_), 33.8 (CH_2_CO_2_Et), 28.7 (C(CH_3_)_3_), 26.1, 25.0 (CH_3_CCH_3_), 14.2 (CH_3_CH_2_). HRMS (ESI+): m/z 352.1728 (M + Na^+^). C_16_H_27_NNaO_6_ requires: 352.1736. **25**: colorless oil. R_f_ = 0.36 (PE:Et_2_O 1:1). GC-MS: R_t_ 9.40 min: m/z 314 (M^+^-15, 0.7), 271 (16), 254 (6.3), 229 (17), 215 (32), 198 (7.7), 184 (16), 173 (5.2), 172 (46), 171 (21), 170 (9.3), 155 (11), 152 (13), 143 (11), 135 (5.4), 129 (7.2), 128 (6.4), 127 (14), 126 (19), 125 (14), 109 (9.9), 101 (18), 100 (6.0), 99 (8.8), 98 (10), 97 (32), 88 (33), 86 (6.7), 85 (100), 84 (17), 83 (7.7), 81 (18), 74 (6.5), 73 (7.8), 71 (12), 70 (9.9), 69 (10), 61 (6.8), 60 (6.2), 59 (24), 58 (35), 57 (82), 56 (8.3), 55 (15), 43 (52), 42 (13), 41 (40), 39 (9.5). ^1^H-NMR (CDCl_3_): δ 6.57 (s, 1H, NH), 4.94 (dd, *J* = 6.4, 2.8 Hz, 1H, H-6a), 4.52 (dd, *J* = 6.4, 3.8 Hz, 1H, H-3a), 4.22 (dt, *J* = 8.2, 4.1 Hz, 1H, H-4), 4.36 (d, *J* = 2.8 Hz, 1H, H-6), 4.19 (q, *J* = 7.2 Hz, 2H, CH_2_CH_3_), 2.66, 2.54 (AB part of an ABX syst., J_AB_ = 15.5 Hz, J_AX_ = 8.1 Hz, J_BX_ = 4.5 Hz., 2H, CH_2_CO_2_Et), 1.54, 1.34 (2 s, 2 × 3H, C(CH_3_)_2_), 1.36 (s, 9H, C(CH_3_)_3_), 1.29 (t, *J* = 7.2 Hz, 3H, CH_2_CH_3_). ^13^C-NMR (CDCl_3_): δ 170.2, 169.1 (C=O), 114.1 (C-2), 84.5 (C-6), 83.8 (C-6a), 83.4 (C-3a), 82.3 (C-4), 61.0 (CH_2_CH_3_), 51.1 (C(CH_3_)_3_), 38.0 (CH_2_CO_2_Et), 28.6 (C(CH_3_)_3_), 27.2, 25.3 (CH_3_CCH_3_), 14.2 (CH_3_CH_2_). HRMS (ESI+): m/z 352.1728 (M + Na^+^). C_16_H_27_NNaO_6_ requires: 352.1730.

*Ethyl 2-((3aR,4S,6S,6aR)-6-(tert-butylcarbamoyl)-2,2-dimethyltetrahydrofuro[3,4-d][1,3]dioxol-4-yl)acetate* (**26**) and *Ethyl 2-((3aR,4R,6S,6aR)-6-(tert-butylcarbamoyl)-2,2-dimethyltetrahydrofuro[3,4-d][1,3]dioxol-4-yl)acetate* (**27**). Compounds **26** and **27** were obtained under the same conditions described for compounds **24** and **25**, starting either from ***syn*-20a** or ***syn*-20b** in 73% and 65% yield respectively, and a 82:18 or 83:17 d.r. As the two diastereomers could not be separated the 82:18 mixture of them was characterized. **26,27**: colorless oil. R_f_ = 0.34 (PE:Et_2_O 1:1). IR (ATR): ν_max_ 3417, 2976, 2936, 2908, 2876, 1734, 1681, 1525, 1456, 1366, 1332, 1288, 1266, 1229, 1209, 1181, 1163, 1101, 1045, 1028, 983, 951, 921, 895, 861, 800, 723. GC-MS: R_t1_ 9.33 min: *m*/*z* 314 (M^+^ − 15, 21), 314 (21), 284 (15), 229 (6.1), 228 (5.5), 184 (9.3), 173 (8.4), 172 (100), 171 (14), 170 (9.3), 155 (16), 154 (11), 152 (10), 143 (6.8), 128 (10), 127 (11), 126 (55), 125 (7.8), 101 (15), 100 (6.0), 99 (11), 98 (22), 97 (11), 88 (12), 87 (22), 85 (26), 84 (13), 83 (6.4), 82 (5.7), 81 (29), 71 (7.2), 70 (5.2), 59 (12), 58 (22), 57 (30), 55 (7.8), 43 (18), 41 (14); R_t2_ 9.42 min: *m*/*z* 314 (M^+^ − 15, 7.1), 314 (7.1), 284 (5.2), 242 (5.8), 229 (15), 215 (16), 212 (8.0), 186 (6.6), 184 (14), 173 (5.7), 172 (47), 171 (69), 170 (18), 155 (15), 154 (16), 146 (8.0), 143 (11), 129 (6.1), 128 (8.1), 127 (11), 126 (29), 125 (29), 115 (5.8), 101 (17), 100 (6.1), 99 (11), 98 (16), 97 (31), 89 (6.7), 88 (47), 87 (5.2), 86 (7.2), 85 (100), 84 (28), 83 (8.2), 82 (6.6), 81 (34), 73 (8.2), 72 (6.0), 71 (14), 70 (13), 69 (11), 61 (7.6), 60 (6.3), 59 (31), 58 (41), 57 (63), 56 (8.4), 55 (15), 44 (5.3), 43 (47), 42 (13), 41 (35), 39 (7.7). From NMR spectra of an 82:18 mixture we could extrapolate the spectra of **26** and **27**. ^1^H-NMR (**26**) (CDCl_3_): δ 6.23 (s, 1H, NH), 4.98 (dd, *J* = 6.0, 4.2 Hz, 1H, H-6a), 4.72 (dd, *J* = 6.0, 3.6 Hz, 1H, H-3a), 4.25–4.12 (m, 2H, CH_2_CH_3_), 4.04 (td, *J* = 6.6, 3.6 Hz, 1H, H-4), 4.01 (d, *J* = 4.2 Hz, 1H, H-6), 2.83, 2.78 (AB part of an ABX syst., *J*_AB_ = 15.2 Hz, J_AX_ = 5.4 Hz, *J*_BX_ = 4.9 Hz., 2H, CH_2_CO_2_Et), 1.43, 1.30 (2 s, 2 × 3H, C(CH_3_)_2_), 1.38 (s, 9H, C(CH_3_)_3_), 1.29 (t, *J* = 7.2 Hz, 3H, CH_2_CH_3_). ^1^H-NMR (**27**) (CDCl_3_): δ 6.35 (s, 1H, NH), 5.02 (dd, *J* = 5.4, 4.5 Hz, 1H, H-6a), 4.66–4.59 (m, 2H, H-3a, H-4), 4.28 (d, *J* = 4.5 Hz, 1H, H-6), 4.25–4.12 (m, 2H, CH_2_CH_3_), 2.51, 2.46 (AB part of an ABX syst., *J*_AB_ = 14.3 Hz, *J*_AX_ = 5.5 Hz, *J*_BX_ = 6.3 Hz., 2H, CH_2_CO_2_Et), 1.47, 1.31 (2 s, 2 × 3 H, (CCH_3_)_2_), 1.38 (s, 9H, C(CH_3_)_3_), 1.27 (t, *J* = 7.2 Hz, 3H, CH_2_CH_3_). ^13^C-NMR (**27**) (CDCl_3_): δ 170.7, 166.3 (C=O), 112.6 (C-2), 81.8 (C-6), 81.6 (C-6a), 80.3 (C-3a), 77.7 (C-4), 60.8 (CH_2_CH_3_), 51.0 (C(CH_3_)_3_), 33.8 (CH_2_CO_2_Et), 28.7 (C(CH_3_)_3_), 26.1, 25.0 (CH_3_CCH_3_), 14.2 (CH_3_CH_2_). ^13^C-NMR (**27**) (CDCl_3_): δ 170.0, 166.6 (C=O), 112.9 (C-2), 83.9 (C-3a), 81.2 (C-6), (the peaks of C-6a and C-4 are covered by the signals of **26**), 60.4 (CH_2_CH_3_), 51.1 (C(CH_3_)_3_), 36.3 (CH_2_CO_2_Et), 28.8 (C(CH_3_)_3_), 26.2, 24.4 (CH_3_CCH_3_), 14.2 (CH_3_CH_2_). HRMS (ESI+): *m/z* 352.1720 (M + Na^+^). C_16_H_27_NNaO_6_ requires: 352.1730.

*(2R,3R,3aS,6aS)-N-(tert-Butyl)-3-hydroxy-5-oxohexahydrofuro[3,2-b]furan-2-carboxamide* (**31**). A solution of **24** (25 mg, 13.2 μmol) in THF (759 μL) and water (379 μL) was treated with CF_3_COOH (379 μL). The reaction was stirred at r.t. for 48 h and then refluxed for 6 days. The crude product was evaporated and chromatographed (AcOEt) to afford **31** (17 mg, 92%) as a white solid. M.p. 153.3–156.1 °C (ACOET). R_f_ = 0.31 (AcOEt). [α]^23^_D_= −20.88 (c = 1.08, CHCl_3_). IR (ATR): ν_max_ 3379, 1812, 1665, 1526,1362, 1310, 1256, 1230, 1199, 1188, 1155, 1141, 1084,1066, 1043 1025, 1005, 939, 908, 861, 845,707, 675, 613. ^1^H-NMR (CDCl_3_): δ 6.19 (s, 1H, N*H*), 5.01 (t, *J* = 4.4, 1H, *H*-3a), 4.92 (ddd, *J* = 6.0, 4.4, 1.5 Hz, 1H, *H*-6a), 4.30 (dt, *J* = 8.1, 4.4 Hz, 1H, *H*-3), 4.11 (d, *J* = 8.1 Hz, 1H, *H*-2), 3.24 (d, *J* = 4.8 Hz, 1H, O*H*), 2.82, 2.74 (AB part of an ABX syst., *J*_AB_ = 18.8 Hz., *J*_AX_ = 1.5 Hz, J_BX_ = 6.0 Hz, 2H, *H*-6), 1.38 (s, 9H, C(C*H*_3_)_3_). ^13^C-NMR (CDCl_3_): δ 174.4, 169.2 (*C*=O), 82.3 (*C*-3a), 79.3 (*C*-2), 76.8 (*C*-6a), 75.3 (*C*-3), 51.4 (*C*(CH_3_)_3_), 36.6 (*C*-6), 28.7 (C(*C*H_3_)_3_). HRMS (ESI+): *m/z* 244.1165 (M + H^+^). C_11_H_18_NO_5_ requires: 244.1177.

*(E)-3-((4R,5S)-5-(((tert-butyldimethylsilyl)oxy)methyl)-2,2-dimethyl-1,3-dioxolan-4-yl)prop-2-en-1-ol* (**35**). It was prepared by reduction of **18**, as previously described, to afford a colorless oil in 96% overall yield [37]. R_f_ = 0.36 (PE:Et_2_O 75:25). The analytical data agree with the reported ones.

*(E)-((4R,5S)-5-(((tert-Butyldimethylsilyl)oxy)methyl)-2,2-dimethyl-4-(3-((4-methoxyphenyl)oxy)prop-2-en-1-yl)-1,3-dioxolane* (**36**). A solution of **35** (116 mg, 0.38 mmol) in dry CH_2_Cl_2_ (3.8 mL) was treated with *p*-methoxyphenol (143 mg, 1.15 mmol), and triphenylphosphine (151 mg, 0.58 mmol). Then diethyl azodicarboxylate (97%, 93 µL, 0.58 mmol) was slowly added at 0 °C. The reaction mixture was then stirred for 4 days at room temperature. The solvent was evaporated under reduced pressure and the residue was purified by column chromatography on silica gel (PE:Et_2_O, 8:2) to afford **36** (138 mg, 88%) as a colorless oil. R_f_ = 0.89 (PE:AcOEt 6:4). [α]^34^_D_= +2.46 (c = 0.95, CHCl_3_). IR (ATR): ν_max_ 2930, 2857, 1507, 1463, 1379, 1228, 1212, 1168, 1096, 1040, 972, 939, 834, 824, 775, 745, 714, 666. GC-MS: R_t_ 11.58 min: *m/z* 408 (M^+^, 2.7), 293 (20), 285 (6.0), 227 (5.4), 211 (12), 201 (11), 181 (19), 169 (17), 165 (9.6), 131 (9.3), 125 (16), 124 (37), 123 (37), 117 (8.4), 116 (9.9), 115 (11), 112 (8.0), 111 (100), 109 (8.0), 101 (7.2), 95 (25), 93 (6.0), 89 (29), 83 (5.9), 81 (6.0), 77 (8.8), 75 (39), 74 (8.7), 73 (90), 69 (52), 67 (8.6), 59 (25), 57 (7.8), 55 (8.4), 53 (18), 45 (6.1), 43 (31), 41 (34), 39 (5.8). ^1^H-NMR (CDCl_3_): δ 6.83 (s, 4H, aromatic H), 5.99 (dt, *J* = 15.6, 4.8 Hz, 1H, C*H*-CH_2_OPMP), 5.91 (dd, *J* = 15.6, 6.5 Hz, 1H, C*H*=CHCH_2_OPMP), 4.69 (t, *J* = 6.5 Hz, 1H, *H*-4), 4.49 (d, *J* = 4.8 Hz, 2H, C*H*_2_OPMP), 4.20 (q, *J* = 6.1 Hz, 1H, *H*-5), 3.76 (OC*H*_3_), 1.47, 1.37 (2 s, 2 × 3H, (C*H*_3_)_2_C), 0.88 (s, 9 H, (C*H*_3_)_3_C), 0.05, 0.04 (2 s, 2 × 3H, C*H*_3_Si). ^13^C-NMR (CDCl_3_): δ 153.9, 152.7 (aromatic quat.), 128.9, 128.3 (C*H*=C*H*), 115.6, 114.6 (aromatic C*H*), 108.6 (*C*-2), 78.5 (*C*-5), 77.7 (*C*-4), 68.4 (*C*H_2_OPMP), 62.2 (*C*H_2_OSi), 55.7 (O*C*H_3_), 27.8, 25.4 ((*C*H_3_)_2_C), 25.9 (C(*C*H_3_)_3_), 18.2 (Si*C*(CH_3_)_2_), −5.4 (Si(*C*H_3_)_2_. HRMS (ESI+): *m/z* 431.2221 (M + Na^+^). C_22_H_36_NaO_5_Si requires: 431.2230.

*((4S,5R)-5-((E)-3-(4-Methoxyphenoxy)prop-1-en-1-yl)-2,2-dimethyl-1,3-dioxolan-4-yl)methanol* (**37**). It was synthesized following the same procedure used for ***ent*-14**, starting from **36** (138 mg, 0.34 mmol). Chromatography with PE:Et_2_O 3:7 afforded **37** as a colorless oil (98 mg, 99%). R_f_ = 0.605(PE:Et_2_O 7:3).

[α]^23^_D_= −33.74 (c = 0.74, CHCl_3_). IR (ATR): ν_max_ 3544, 3496, 2983, 2927, 2872, 1592, 1504, 1463, 1408, 1385, 1339, 1309, 1292, 1235, 1211, 1184, 1162, 1136, 1109, 1075, 1061, 1028, 1014, 977, 945, 897, 870, 851, 828, 800, 744, 649. GC-MS: R_t_ 9.89 min: *m/z* 294 (M^+^, 2.6), 125 (9.1), 124 (100), 123 (15), 111 (5.3), 109 (14), 95 (5.7), 81 (8.4), 69 (6.6), 59 (21), 57 (5.7), 55 (6.6), 53 (8.8), 44 (7.2), 43 (21), 41 (9.9). ^1^H-NMR (CDCl_3_): δ 6.83 (s, 4H, aromatic H), 6.04 (dt, *J* = 15.6, 5.1 Hz, 1H, C*H*-CH_2_OPMP), 5.86 (dd, *J* = 15.6, 7.2 Hz, 1H, C*H*=CHCH_2_OPMP), 4.70 (t, *J* = 7.0 Hz, 1H, H-5), 4.51 (d, *J* = 5.0 Hz, 2H, CH_2_OPMP), 4.26 (q, *J* = 6.0 Hz, 1H, *H*-4), 3.77 (OCH_3_), 3.55 (t, *J* = 5.6 Hz, 1H, O*H*), 1.51, 1.39 (2 s, 2 × 3H, (CH_3_)_2_C). ^13^C-NMR (CDCl_3_): δ 154.0, 152.5 (aromatic quat.), 130.1, 127.6 (CH=CH), 115.8, 114.6 (aromatic CH), 108.9 (*C*-2), 78.3 (*C*-4), 77.2 (*C*-5), 68.1 (CH_2_OPMP), 62.0 (*C*H_2_OH), 55.7 (O*C*H_3_), 27.8, 25.2 ((CH_3_)_2_C). HRMS (ESI+): *m/z* 317.2473 (M + Na^+^). C_16_H_22_ NaO_5_ requires: 317.2462.

## 4. Conclusions

In conclusion, we have presented another insight into diastereoselective Passerini reaction were a bio-based chiral aldehyde is involved. Moreover, we exploited the additional functional groups for expanding scaffold diversity, leading to the formation of different oxygenated heterocycles.

## Figures and Tables

**Table 1:** Preliminary experiments using ***ent*-15**, obtained by TEMPO/PhI(OAc)_2_ oxidation of ***ent*-14**.

Entry ^1^	Solvent	Additive (equiv.)	Temperature (°C)	Yield (%)	*anti*-20a:*syn*-20a ^2^
1 ^3^	CH_2_Cl_2_	none	r.t.	67	61:39
2 ^3^	CH_2_Cl_2_/THF	ZnBr_2_ (0.4)	r.t.	51	76:24
3 ^4^	CH_2_Cl_2_/THF	ZnBr_2_ (0.4)	r.t.	55	71:29

Notes: ^1^ 1.1 equivalents of isocyanide. ^2^ Determined by ^1^H-NMR; the relative configuration of compounds **20a** was determined as reported in the Supplementary Materials; ^3^ no drying procedure of the aldehyde. ^4^ In the presence of 3 Å MS.

**Table 2:** Optimization of the stereoselective Passerini reaction.

Entry ^1^	Product	R ^1^	Solvent	Additive (equiv.)	Temperature (°C)	Time	Yield (%) ^2^	*anti*-20:*syn*-20 ^3^
1	**20a**	Me	CH_2_Cl_2_	none	20	1.5 h	92	59:41
2	**20a**	Me	THF	none	20	42 h	97	51:49 ^4^
3	**20a**	Me	THF	ZnBr_2_ (0.4)	20	4 h	78	80:20
4	**20a**	Me	THF	Zn(OAc)_2_ (1.1) ^5^	20	19 h	79	56:44
5	**20b**	Ph	THF	none	20	23 h	97	45:55
6	**20b**	Ph	THF	ZnBr_2_ (0.4)	20	17 h	59	76:24
7	**20b**	Ph	THF	ZnBr_2_ (0.4)	0	16 h	54	74:26
8	**20b**	Ph	THF	ZnBr_2_ (0.4)	−30	6 d	39	65:35
9	**20b**	Ph	THF	Zn(OBz)_2_ (1.1) ^5^	20	5 h	36	64:36

Notes: ^1^ The crude aldehyde was thoroughly dried by azeotropic water removal with toluene and 1.1 equivalents of isocyanide and carboxylic acid were used. ^2^ Overall yield of all stereoisomers over two steps. ^3^ Determined by ^1^H-NMR in all cases and by HPLC as well (entries 5–9); the relative configuration of compounds **20** was determined as reported in the Supplementary Materials. ^4^ Also minor amounts of *trans* isomers were detected. ^5^ In these cases, no carboxylic acid was added in the MCR.

**Table 3:** Scope of the ZnBr_2_ promoted Passerini.

Entry ^1^	Compd	R ^1^	R ^2^	Yield (%) ^2^	*anti*-20: *syn*-20
1	**20a**	Me	*t*-Bu	78	80:20 ^3^
2	**20b**	Ph	*t*-Bu	59	75:25 ^4,5^
3	**20c**	CH_2_ = CH(CH_2_)_2_	*t*-Bu	53	78:22 ^5^
4	**20d**	Me	2,3-(Me)_2_C_6_H_3_	51	93:7 ^3,4^
5	**20e**	Me	−C(CH_2_CH=CH_2_)(CO_2_Et)_2_	64	98:2 ^3,4^
6	**20f**	Ph	2,3-(Me)_2_C_6_H_3_	57	90:10 ^3^
7	**20g**	Ph	4-OBn-C_6_H_4_(CH_2_)_2_	63	85:15 ^3^
8	**20h**	2-Me-C_6_H_4_	Bn	56	88:12 ^3^
9	**20i**	MeOCH_2_	*c*-Hex	64	84:16 ^3^

Notes: ^1^ The crude aldehyde, obtained by Swern oxidation of ***ent*-14**, was thoroughly dried by azeotropic water removal with toluene and all reactions are performed at 20 °C in THF, using 1.1 equivalents of carboxylic acid and isocyanide and 0.4 equivalents of ZnBr_2;_ the average duration is around 20 h. ^2^ Yields over two steps. ^3^ By ^1^H-NMR. ^4^ Only in these cases we observed the formation of a considerable amount of truncated Passerini products which were acylated in situ. ^5^ By HPLC.

**Table 4:** Michael cyclization of compounds **20a** and **20b**.

Entry ^1^	Compd	Conditions ^1^	Time (h)	Temperature	Yield (%)	24:25 (from *anti*) ^3,4^ 26:27 (from *syn*) ^3,4^
1	***anti*-20a**	KOH (1 M)	1	r.t.	33	91:9
2	***anti*-20a**	EtONa (0.1 M) ^2^	4	r.t.	58	90:10
3	***anti*-20b**	EtONa (0.1 M) ^2^	4	r.t.	73	92:8
4	***anti*-20b**	EtONa (0.1 M) ^2^	22	0 °C	83	89:11
5	***anti*-20b**	EtOH:Et_3_N 5:1:1	94	r.t. (67 h), 60 °C (24 h)	74	82:18
6	***syn*-20a**	KOH (1 M)	1	r.t.	16	84:16
7	***syn*-20a**	EtONa (0.1 M) ^2^	5.5	r.t.	73	82:18
8	***syn*-20b**	EtONa (0.1 M) ^2^	6	r.t.	65	83:17

Notes: ^1^ All reactions were performed in EtOH as solvent. ^2^ Prepared in situ from EtOH and Na. ^3^ By ^1^H-NMR. ^4^ The reported relative configurations have been determined as reported in the Supplementary Materials.

## Supplementary Materials

The following are available online: Determination of the relative configuration of **16**, **20a–i**, and **31**. Copies of ^1^H-NMR and ^13^C-NMR spectra. Determination of diasteromeric ratios by ^1^H-NMR and HPLC.
