# Supplementary material for: Synthesis of Polyoxygenated Heterocycles by Diastereoselective Functionalization of a Bio-Based Chiral Aldehyde Exploiting the Passerini Reaction
Source: Molecules. 2020 Jul 15;25(14):3227. doi: 10.3390/molecules25143227 (PMC7397154; doi:10.3390/molecules25143227)
Supplement: Supplementary file 1 [file molecules-25-03227-s001.pdf]

SUPPORTING INFORMATION

Synthesis of polyoxygenated heterocycles by diastereoselective functionalization of a bio-based chiral aldehyde exploiting the Passerini reaction

Gabriella Vitali Forconesi [1], Luca Banfi [1], Andrea Basso [1], Chiara Lambruschini [1], Lisa Moni [1], and Renata Riva [2,*]

[1] Dipartimento di Chimica e Chimica Industriale, Università di Genova, via Dodecaneso 31, 16146 Genova, Italy
[2] Dipartimento di Farmacia, Università di Genova, viale Cembrano 4, 16147 Genova, Italy
* Correspondence: renata.riva@unige.it; Tel.: +39-010-3536106

CONTENTS

1. **Determination of the relative configuration of compounds 16, 20a-i, 24-27, and 31 ____ 2**
2. **References ____ 4**
3. **Copies of ^{1}H-NMR and ^{13}C-NMR spectra ____ 5**
4. **Determination of diastereomeric ratios by ^{1}H-NMR ____ 47**
5. **Determination of diastereomeric ratios by HPLC ____ 52**

1. Determination of the relative configuration of compounds 16, 20a-i, 24-27, and 31

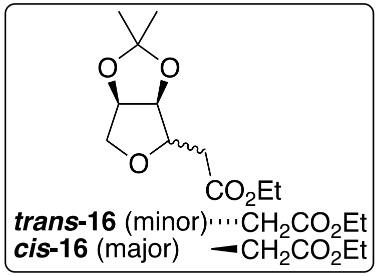

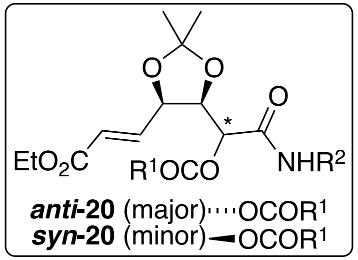

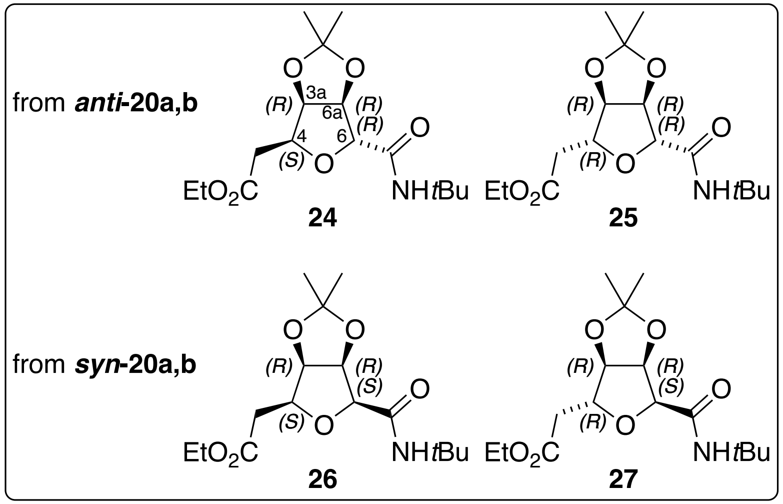

Since bicyclic compounds **24-27** are pretty rigid the examination of ^{1}H-NMR spectra and their comparison with those of similar compounds allowed us to establish the relative configuration of all stereogenic centers. In particular the *trans* relationship between H_6 and H_{6a} in **24** is demonstrated by $J_{6,6a} \sim 0$ Hz, as the result of a dihedral angle close to 90°, while if a *cis* correlation is present $J_{6,6a} \sim 4$ Hz. The *cis* or *trans* connection between H_{3a} and H_4 has been initially established by similar reasoning, because $J_{3a,4} \sim 4$ Hz. Moreover proton and ^{13}C δ values for the methylene bonded to C_4 agree with those of **16**, which was already described [1], and a further evidence has been found after the additional experiments reported below.

In agreement with our previous results [2], establishing the *R* configuration for C_6 (**24** and **25**) allows to assign the relative configuration *R* to the stereogenic center generated during the Passerini reaction as well, which means that the major ***anti*-20** derivatives, all displaying NMR similarities, have the same *R* configuration. Therefore ***syn*-20** compounds must have the opposite *S* configuration.

Additional experimental evidence on the stereochemistry of the above mentioned compounds has been found analyzing bicyclic compound **31**. On this compound we performed MM2 calculations and we were able to establish that the structure of the lactone moiety is pretty rigid, while the tetrahydrofuran ring has various envelope or twist conformations. Among them the twist conformation shown in Figure 1, which allows a hydrogen bond between the OH group and the amidic oxygen and another hydrogen bond between the NH and an oxygen of the ring, seems to be the most stable one. Furthermore, it perfectly agrees with the coupling constants measured in the proton spectrum. These data are also in agreement with the spectra of compounds **29a** and **29b**, which were previously reported by Singh [3]. **31** shares with **29a,b** the same bicyclic system. As far as it concerns stereochemistry, **29a** is the epimer at C-3 of *ent*-**31**, whereas **29b** is the epimer at C-2 of **31**.

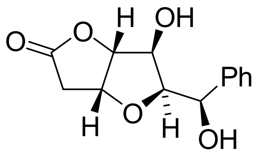

29a

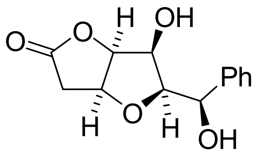

29b

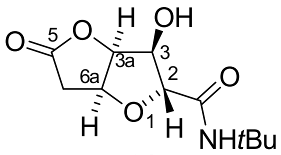

31

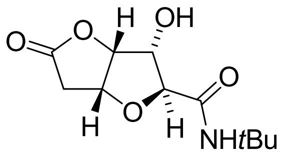

ent-**31**

The twist conformation shown below in Figure 1 has these dihedral angles between the hydrogens:
H_2-C_2-C_3-H_3: 168.1°
H_3-C_3-C_{3a}-H_{3a}: -39.7°
H_{3a}-C_{3a}-C_{6a}-H_{6a}: 20.6°
H_6-C_6-C_{6a}-H_{6a}: -25.8°, 97.5°

These dihedral angles fit well with the observed J:
$J_{2,3}$ = 8.3 Hz
$J_{3\text{-}3a}$ = 4.4 Hz.
$J_{3a\text{-}6a}$ = 4.4 Hz
$J_{6\text{-}6a}$ = 1.5, 5.8 Hz.

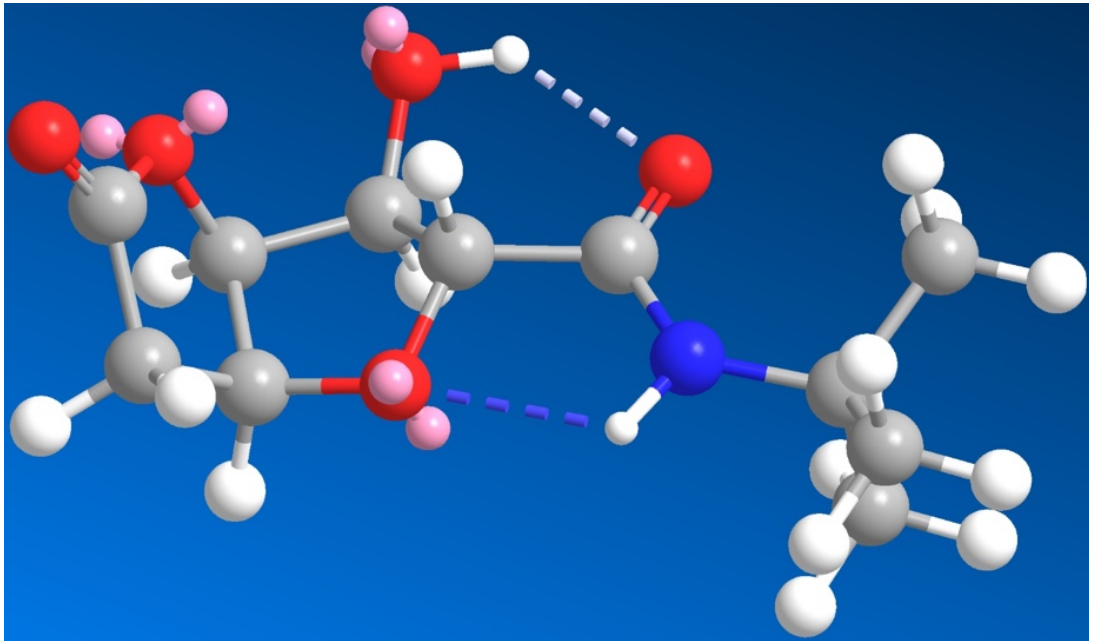

Figure 1. Model of compound **31**.

2. References

1. Buchanan, J. G.; Edgar, A. R.; Hewitt, B. D. A new route to chiral hydroxypyrrolidines from D-erythrose via intramolecular 1,3-cycloaddition. *J. Chem. Soc., Perkin Trans. 1* **1987**, 2371-2376. 10.1039/P19870002371.
2. Moni, L.; Banfi, L.; Basso, A.; Martino, E.; Riva, R. Diastereoselective Passerini Reaction of Biobased Chiral Aldehydes: Divergent Synthesis of Various Polyfunctionalized Heterocycles. *Org. Lett.* **2016**, *18*, 1638-1641. 10.1021/acs.orglett.6b00487.
3. Shing, T. K. M.; Tsui, H.-c. Goniofufurone: synthesis and absolute configuration. *J. Chem. Soc., Chem. Commun.* **1992**, 432-432. 10.1039/C39920000432. Shing, T. K. M.; Tsui, H.-C. Enantiospecific syntheses of (3S,4R)- and (3S,4R,7S)-diastereoisomers of goniofufurone. *Tetrahedron: Asymmetry* **1994**, *5*, 1269-1274. 10.1016/0957-4166(94)80168-1.

3. Copies of ^{1}H-NMR and ^{13}C-NMR spectra

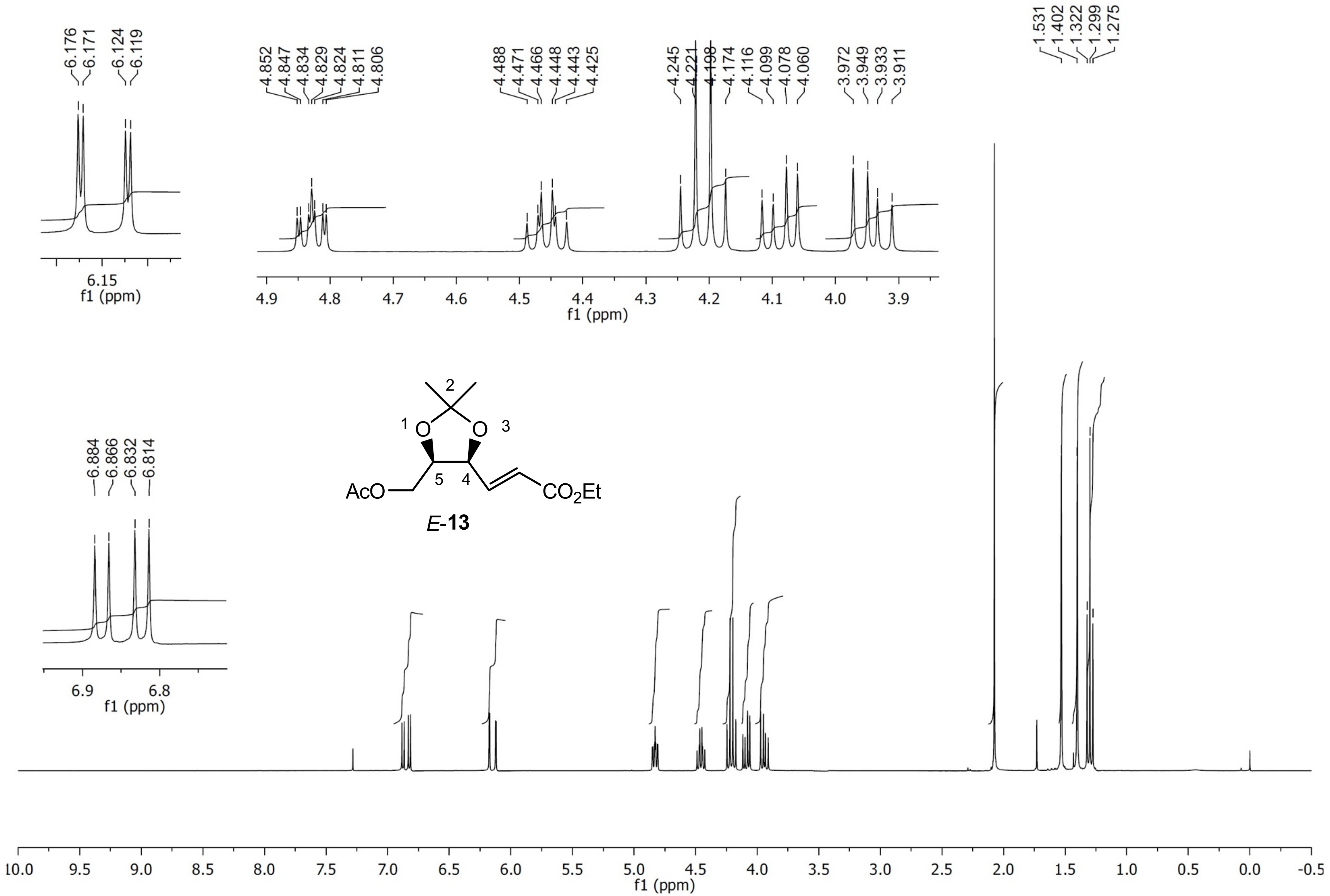

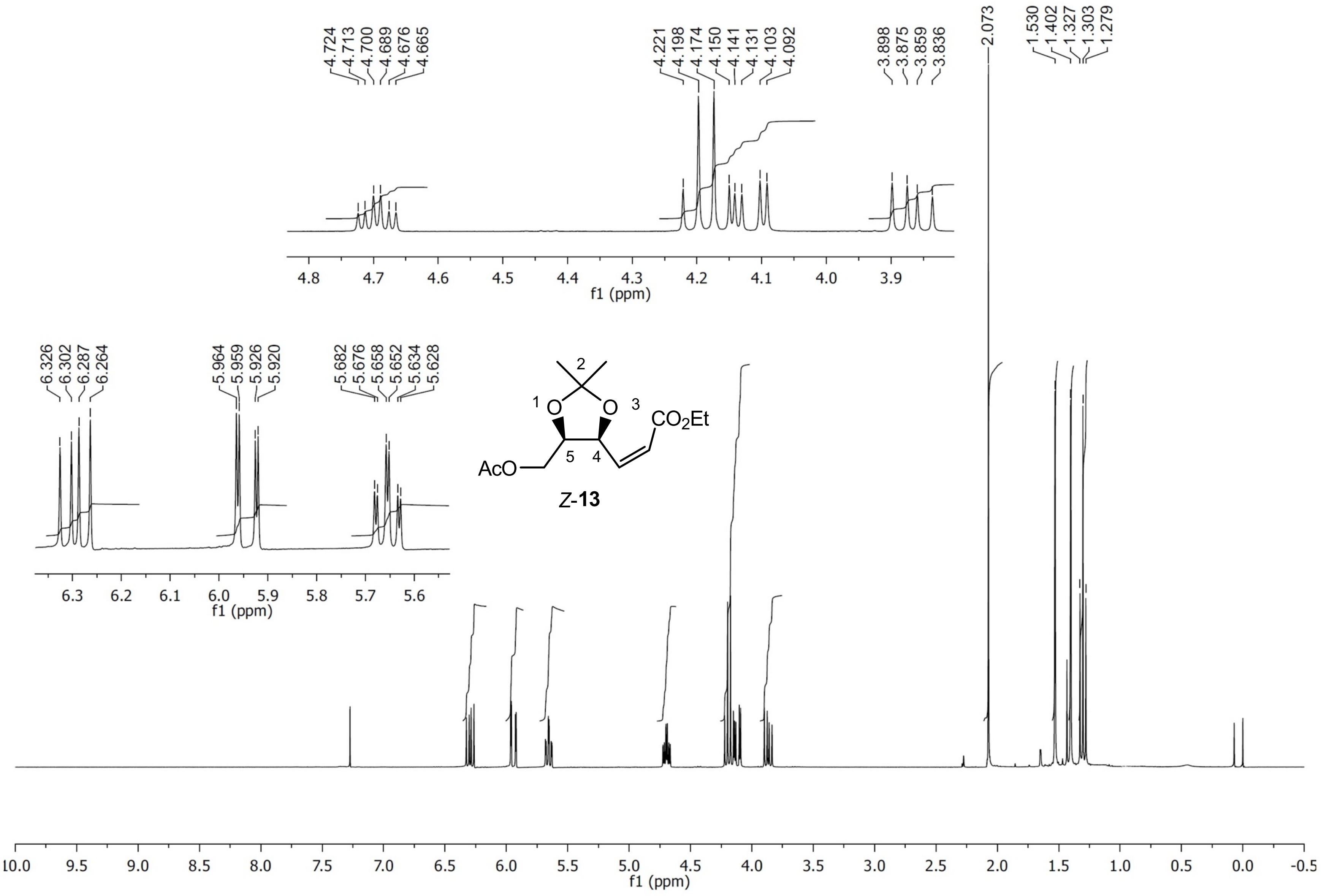

4.724 4.713 4.700 4.689 4.676 4.665
4.221 4.198 4.174 4.150 4.141 4.131 4.103 4.092
3.898 3.875 3.859 3.836
2.073
1.530 1.402 1.327 1.303 1.279
6.326 6.302 6.287 6.264
5.964 5.959 5.926 5.920
5.682 5.676 5.658 5.652 5.634 5.628
f1 (ppm)
1 O 2 O 3
CO_2Et
AcO
5 4
Z-**13**

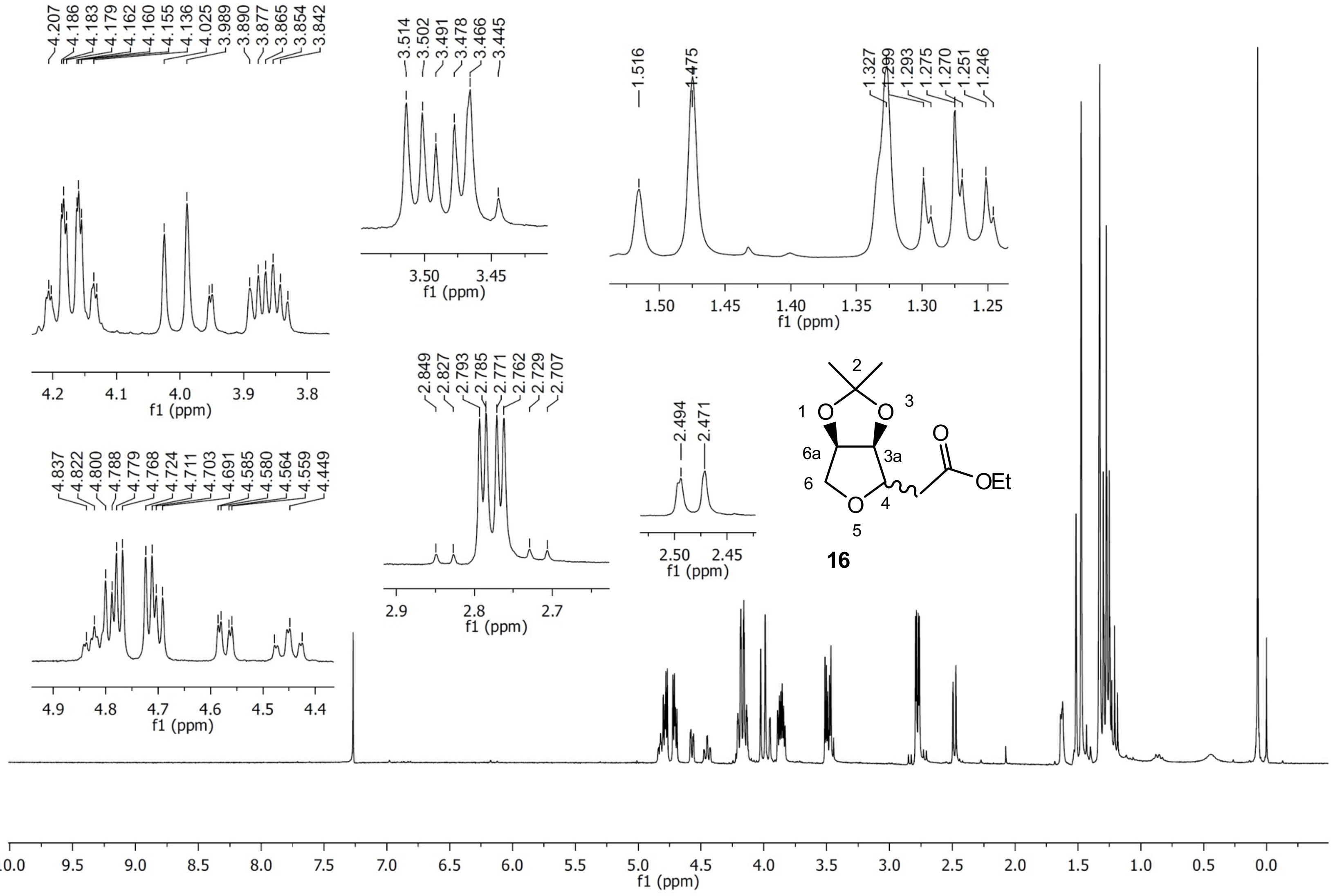

4.207
4.186
4.183
4.179
4.162
4.160
4.155
4.136
4.025
3.989
3.890
3.877
3.865
3.854
3.842
3.514
3.502
3.491
3.478
3.466
3.445
1.516
1.475
1.327
1.299
1.293
1.275
1.270
1.251
1.246
2.849
2.827
2.793
2.785
2.771
2.762
2.729
2.707
2.494
2.471
4.837
4.822
4.800
4.788
4.779
4.768
4.724
4.711
4.703
4.691
4.585
4.580
4.564
4.559
4.449
f1 (ppm)
1
2
3
6a
3a
6
4
5
O
OEt
16

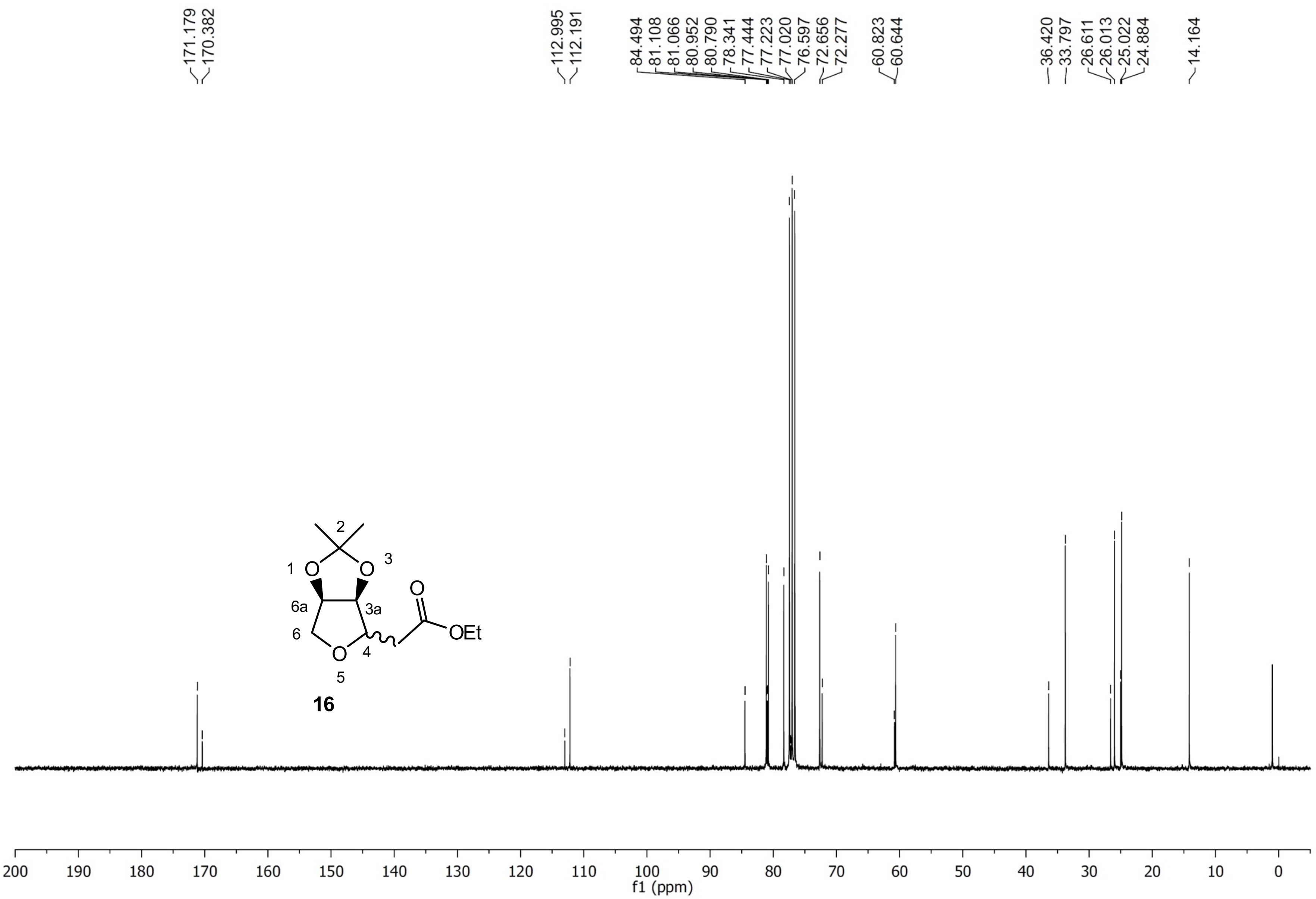
171.179
170.382
112.995
112.191
84.494
81.108
81.066
80.952
80.790
78.341
77.444
77.223
77.020
76.597
72.656
72.277
60.823
60.644
36.420
33.797
26.611
26.013
25.022
24.884
14.164
1
2
3
O
O
6a
3a
O
OEt
6
O
4
5
16
200 190 180 170 160 150 140 130 120 110 100 90 80 70 60 50 40 30 20 10 0
f1 (ppm)

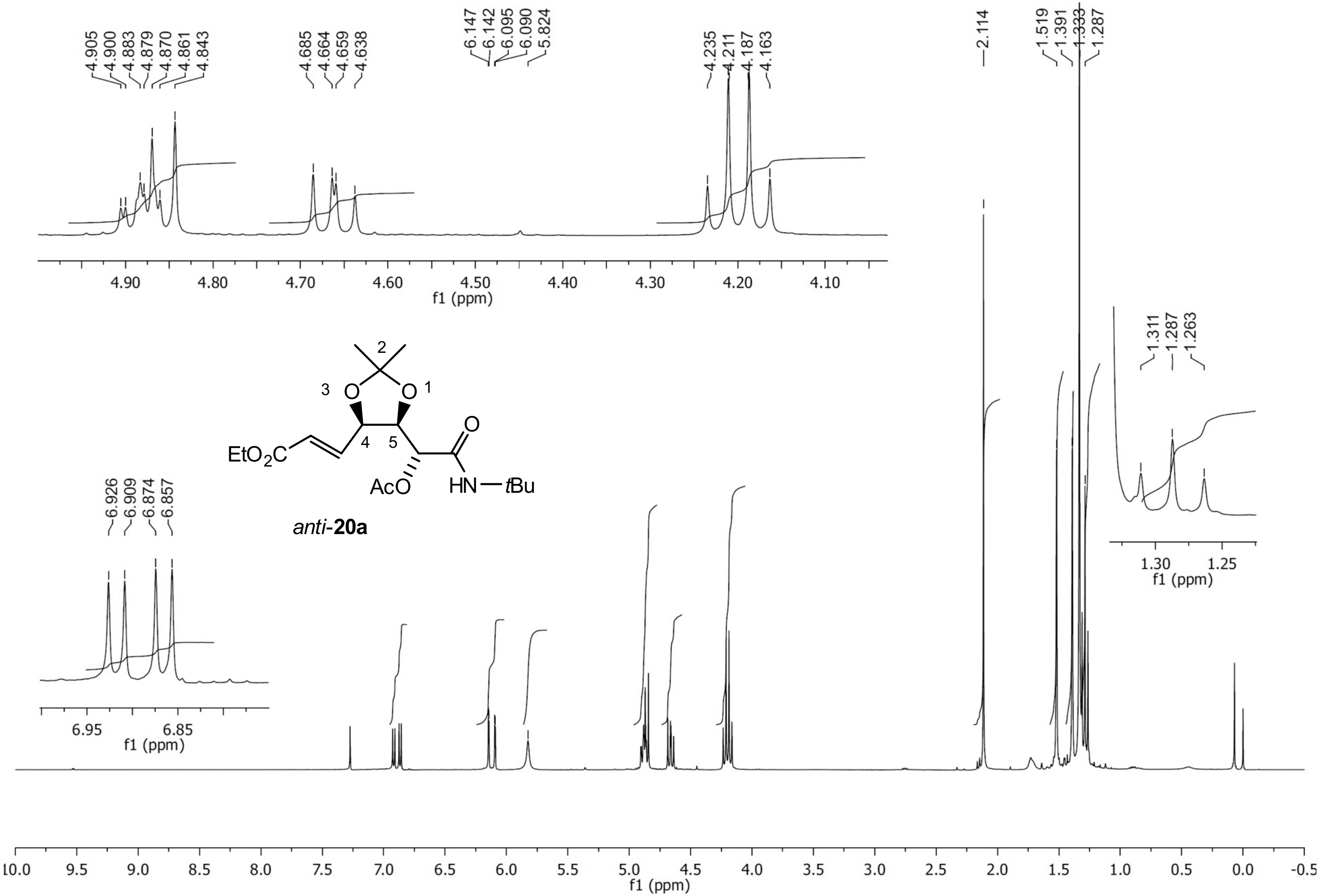
4.905
4.900
4.883
4.879
4.870
4.861
4.843
4.685
4.664
4.659
4.638
6.147
6.142
6.095
6.090
5.824
4.235
4.211
4.187
4.163
2.114
1.519
1.391
1.333
1.287
f1 (ppm)
1.311
1.287
1.263
6.926
6.909
6.874
6.857
EtO2C
AcO
HN
tBu
anti-20a

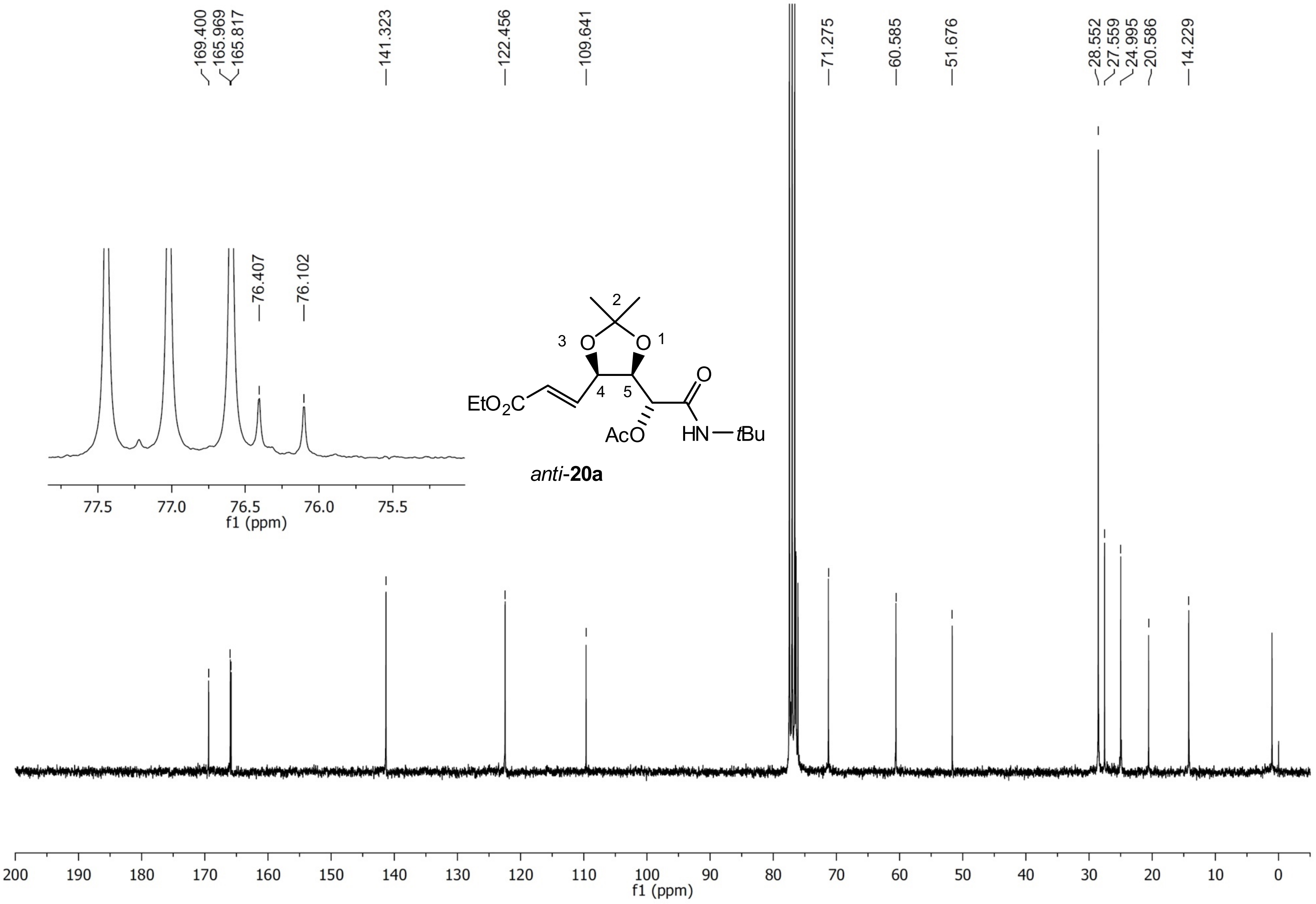
169.400
165.969
165.817
141.323
122.456
109.641
71.275
60.585
51.676
28.552
27.559
24.995
20.586
14.229
76.407
76.102
77.5
77.0
76.5
76.0
75.5
f1 (ppm)
2
3
1
O
O
4
5
EtO2C
AcO
HN
tBu
anti-20a
200
190
180
170
160
150
140
130
120
110
100
90
80
70
60
50
40
30
20
10
0
f1 (ppm)

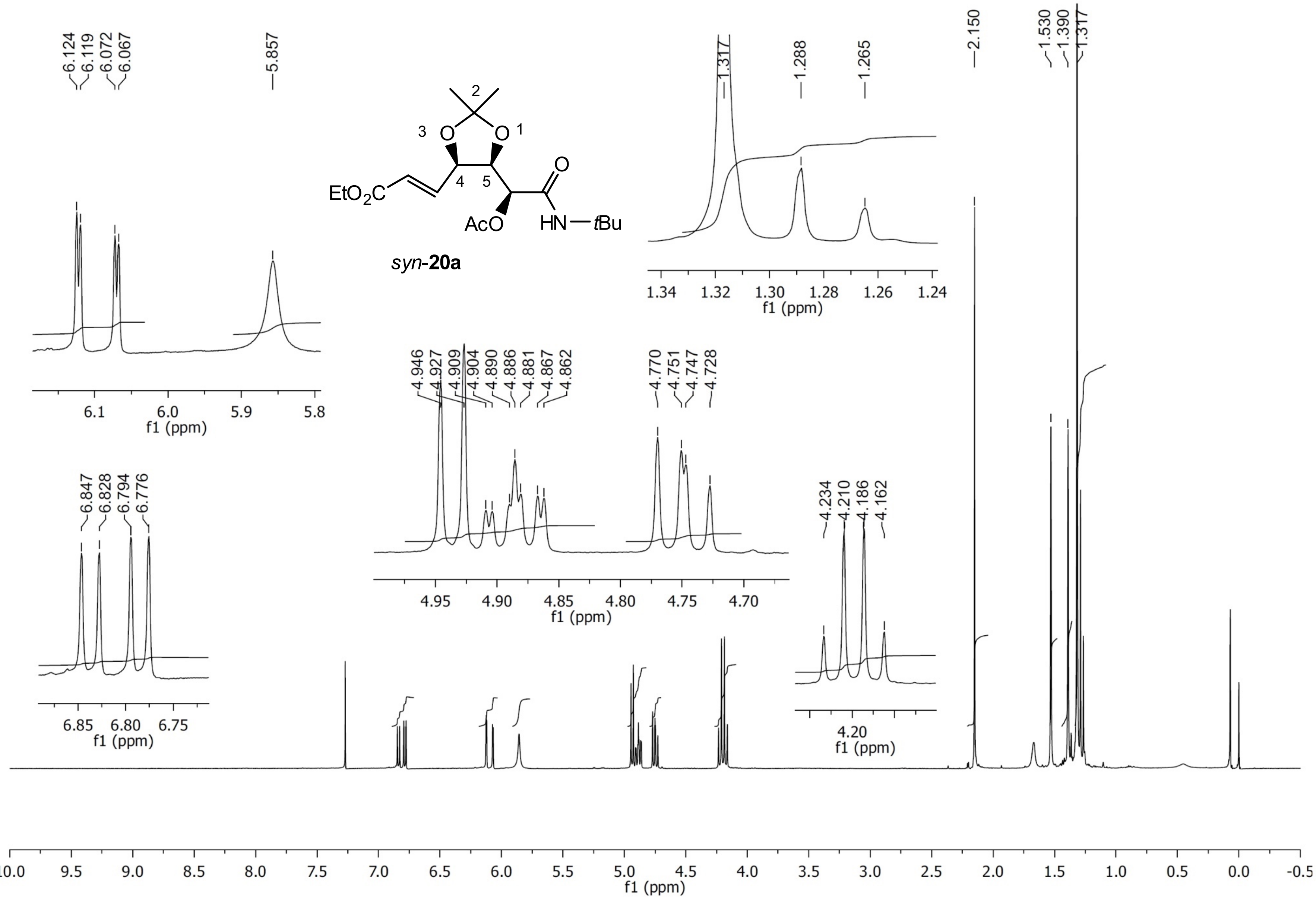
syn-20a
EtO2C
AcO
HN—tBu
6.124
6.119
6.072
6.067
5.857
1.317
1.288
1.265
2.150
1.530
1.390
1.317
4.946
4.927
4.909
4.904
4.890
4.886
4.881
4.867
4.862
4.770
4.751
4.747
4.728
6.847
6.828
6.794
6.776
4.234
4.210
4.186
4.162
f1 (ppm)

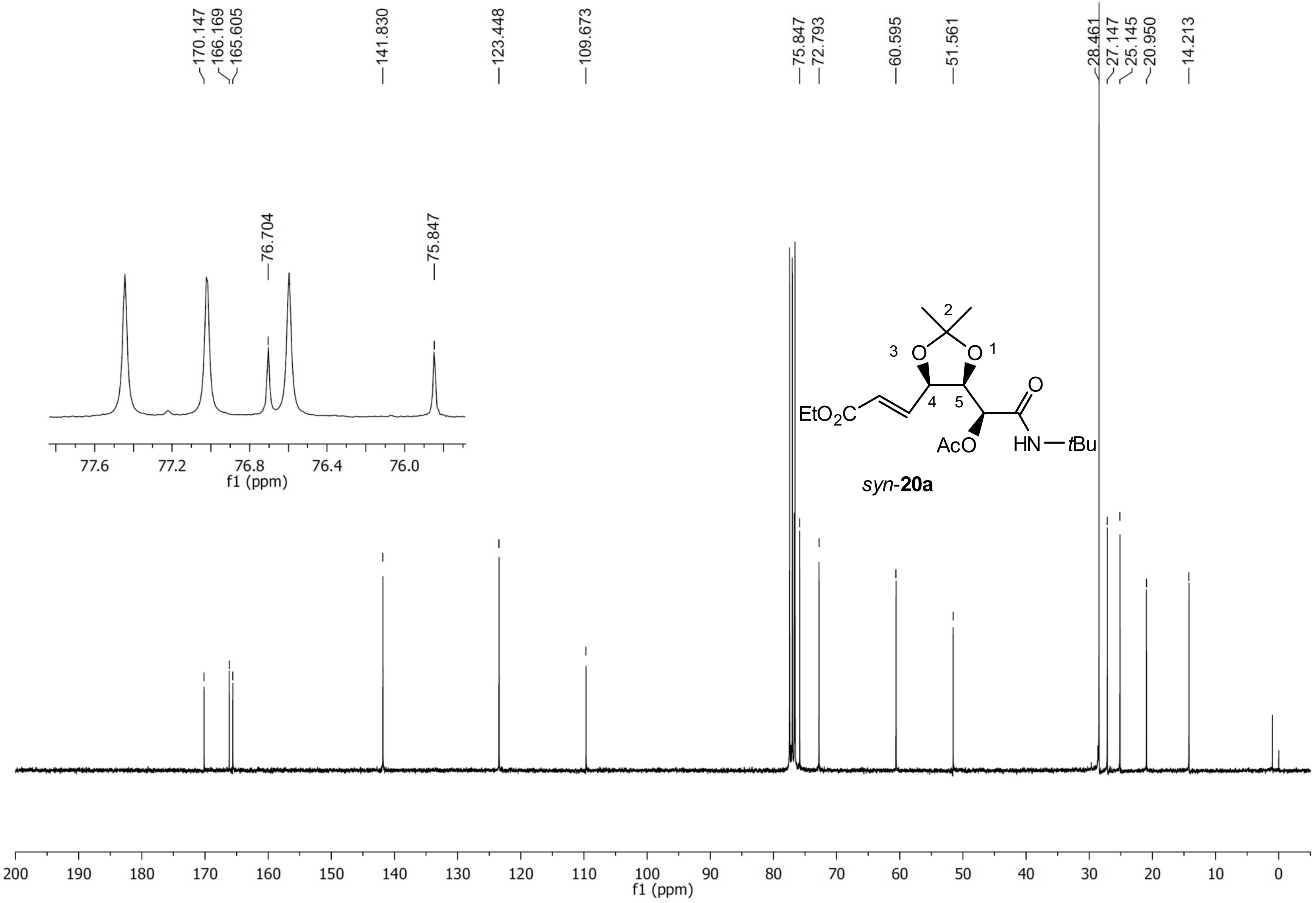

170.147
166.169
165.605
141.830
123.448
109.673
75.847
72.793
60.595
51.561
28.461
27.147
25.145
20.950
14.213
76.704
75.847
77.6
77.2
76.8
76.4
76.0
f1 (ppm)
2
3
1
O
O
4
5
O
EtO2C
AcO
HN
tBu
syn-20a
200
190
180
170
160
150
140
130
120
110
100
90
80
70
60
50
40
30
20
10
0
f1 (ppm)

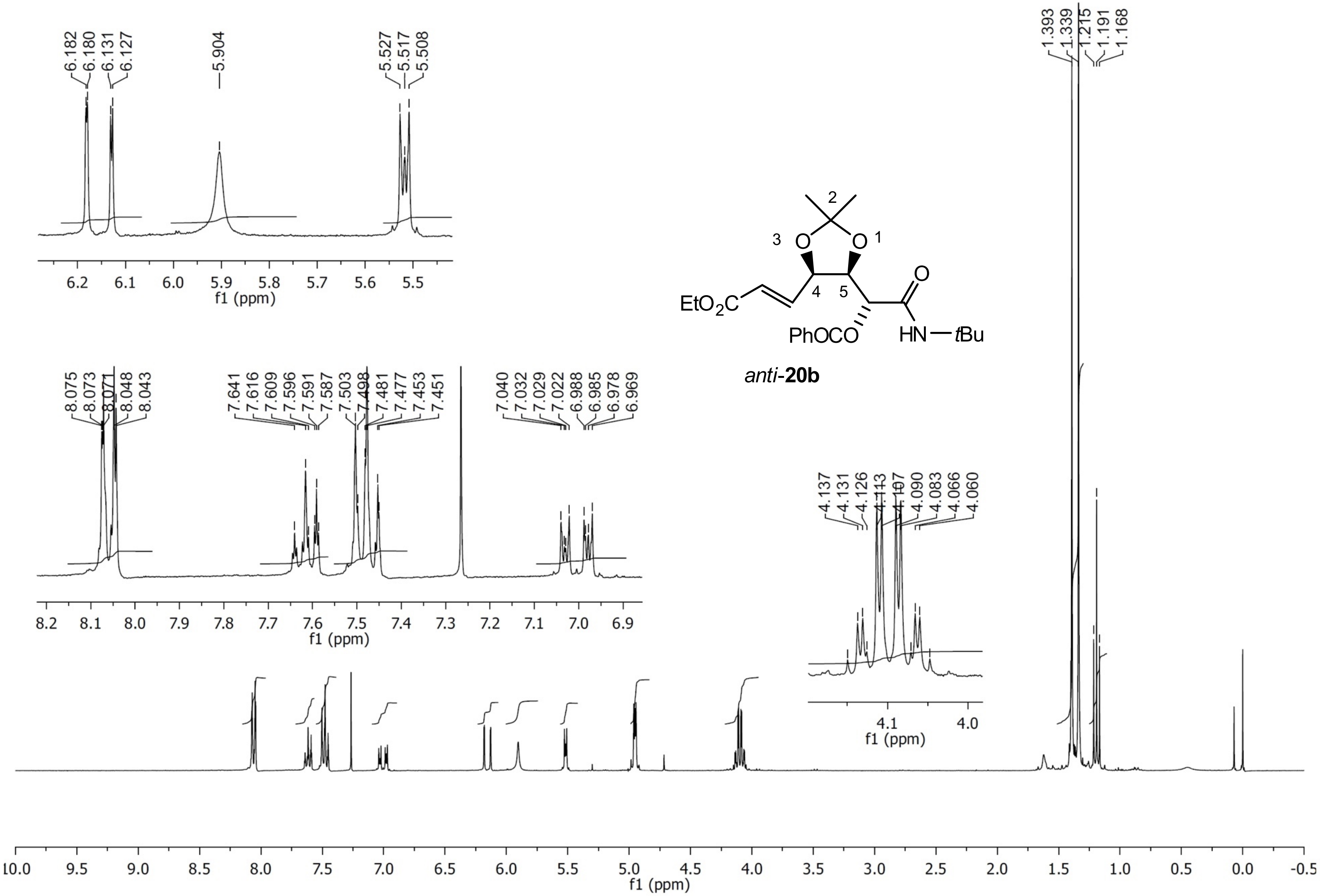
anti-20b
EtO2C
PhOCO
HN—tBu
f1 (ppm)

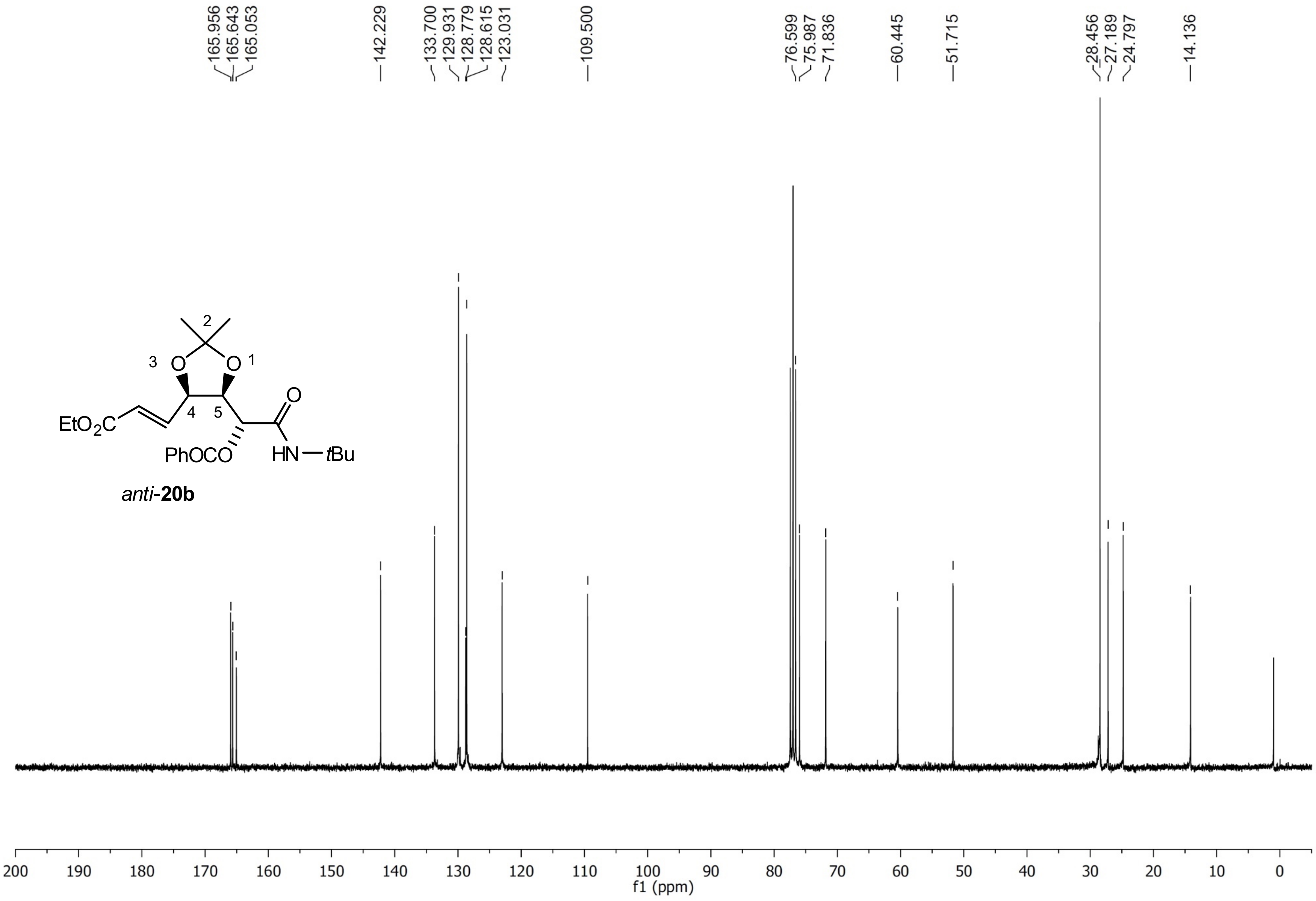

165.956
165.643
165.053
142.229
133.700
129.931
128.779
128.615
123.031
109.500
76.599
75.987
71.836
60.445
51.715
28.456
27.189
24.797
14.136
2
3
O
O
1
O
EtO2C
4
5
PhOCO
HN
tBu
anti-20b
200
190
180
170
160
150
140
130
120
110
100
90
80
70
60
50
40
30
20
10
0
f1 (ppm)

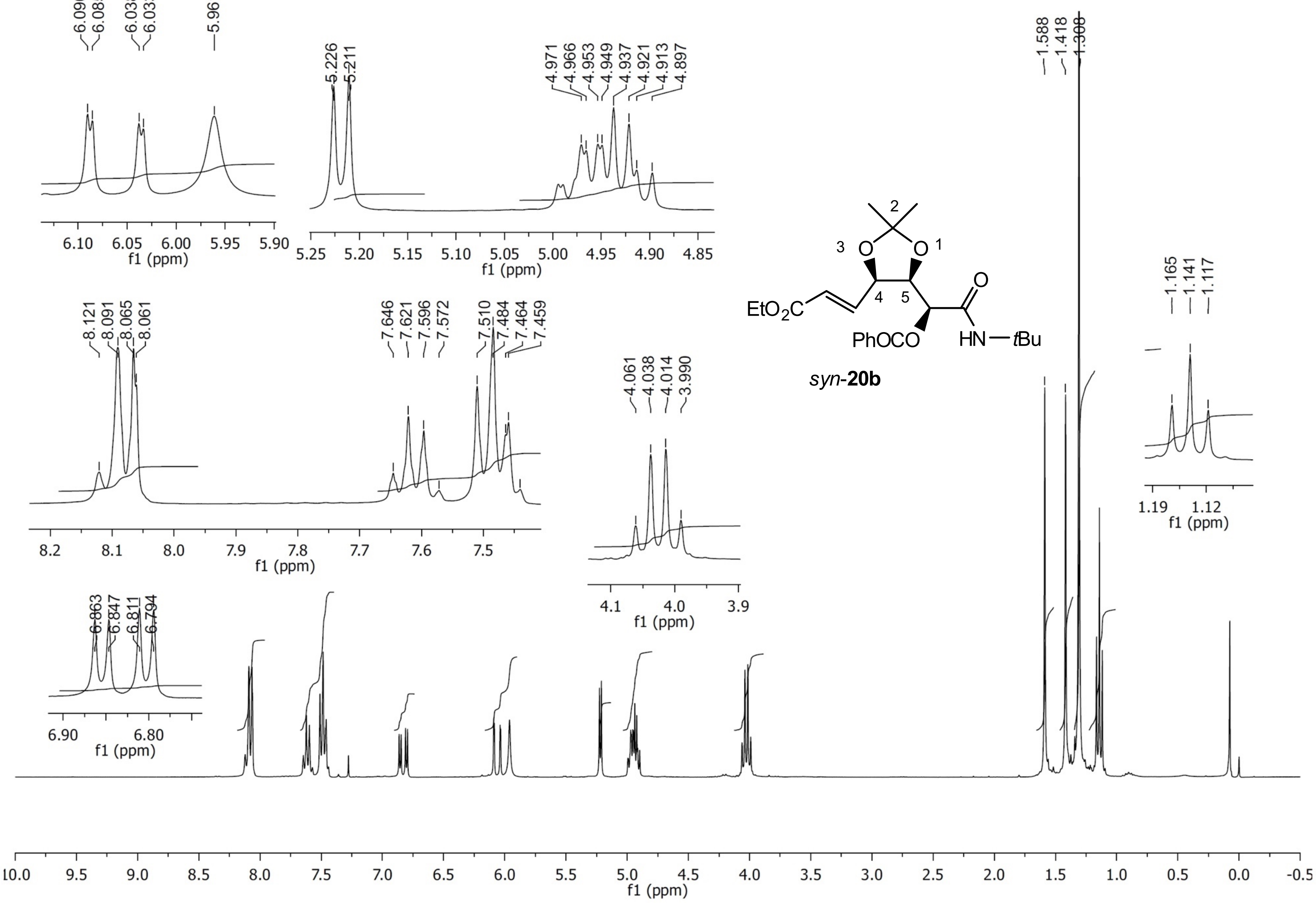

f1 (ppm)
syn-20b
EtO2C
PhOCO
HN
tBu

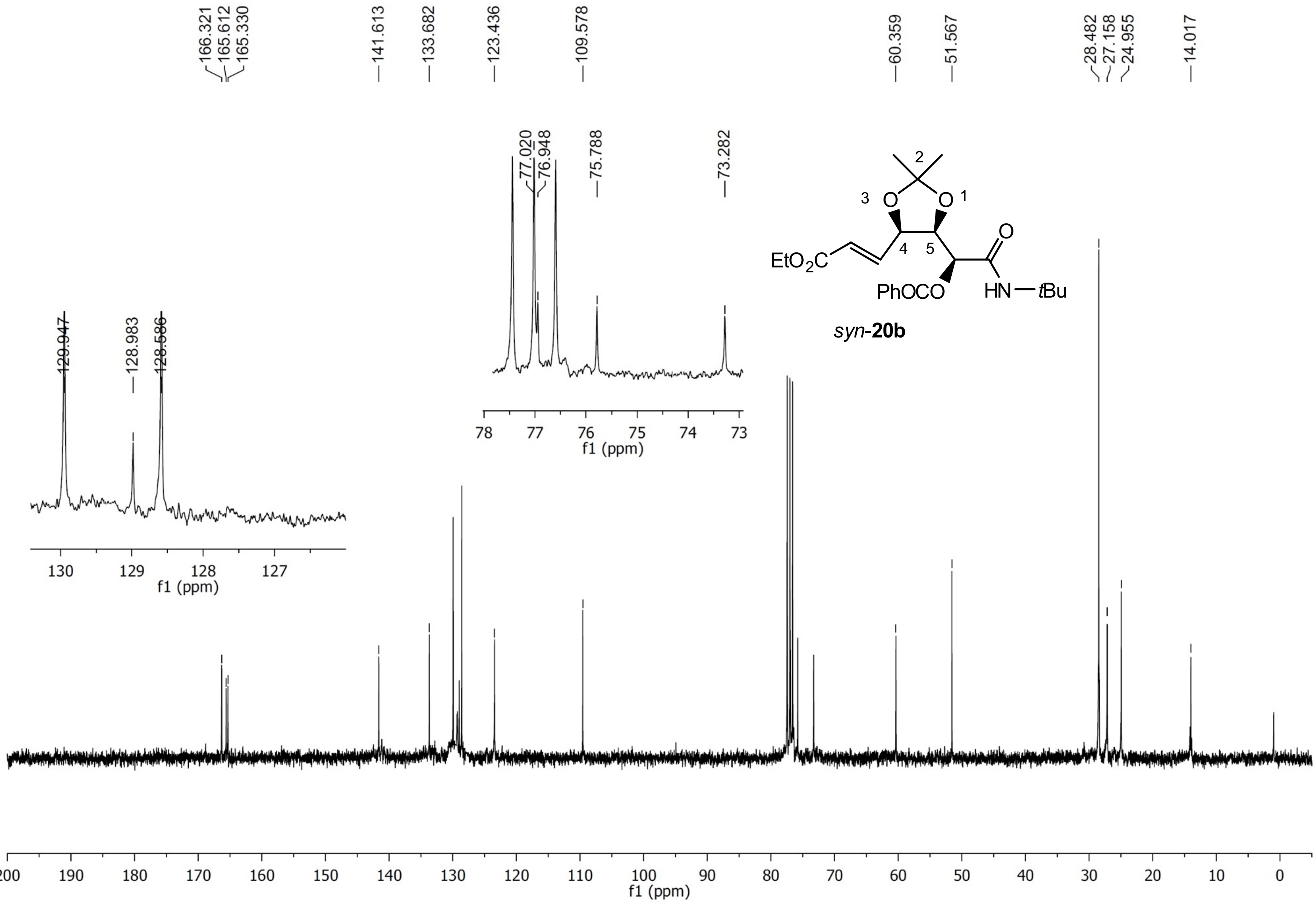
166.321
165.612
165.330
141.613
133.682
123.436
109.578
60.359
51.567
28.482
27.158
24.955
14.017
77.020
76.948
75.788
73.282
129.947
128.983
128.586
f1 (ppm)
EtO2C
PhOCO
HN—tBu
syn-20b

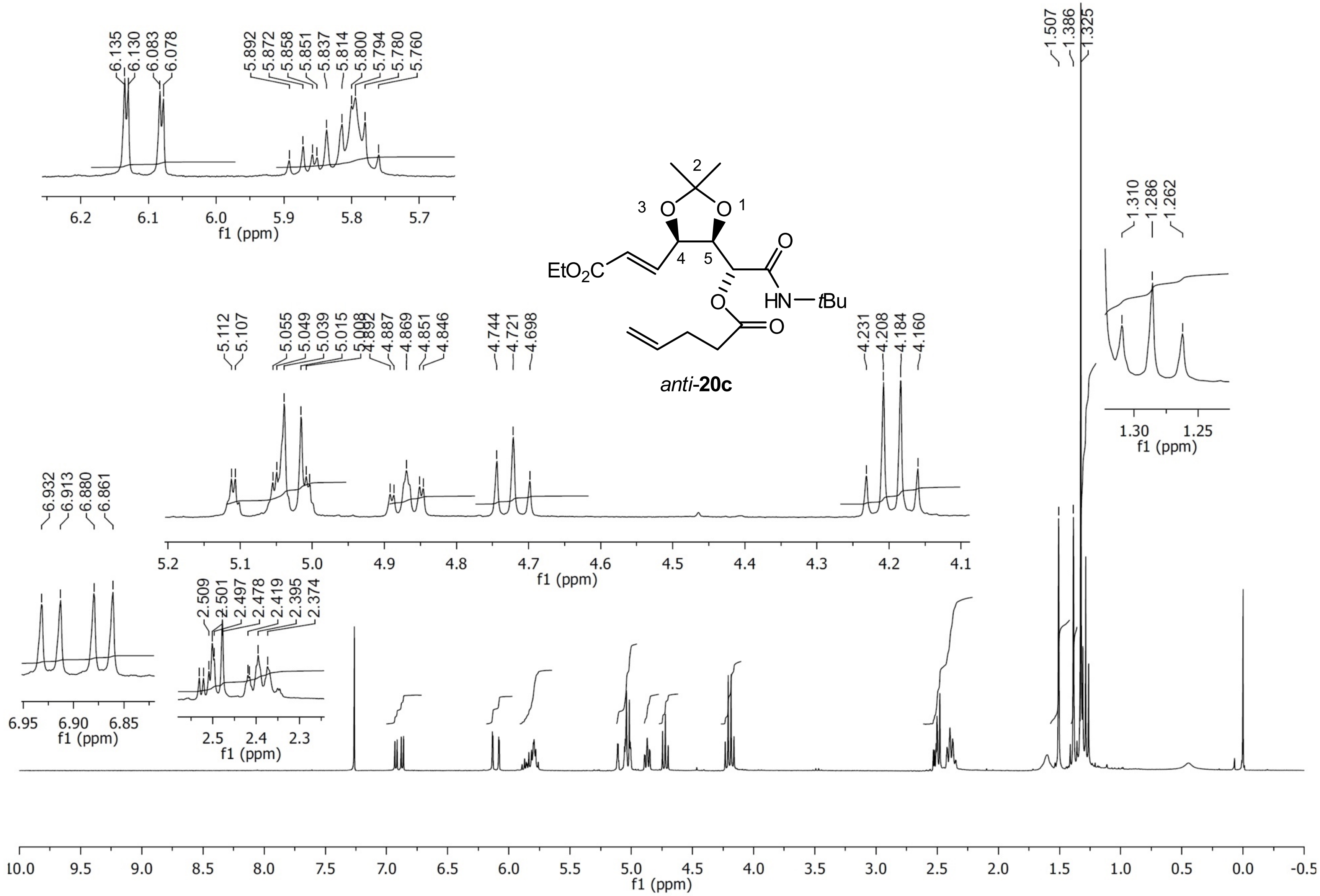

EtO2C
HN—tBu
anti-20c
f1 (ppm)

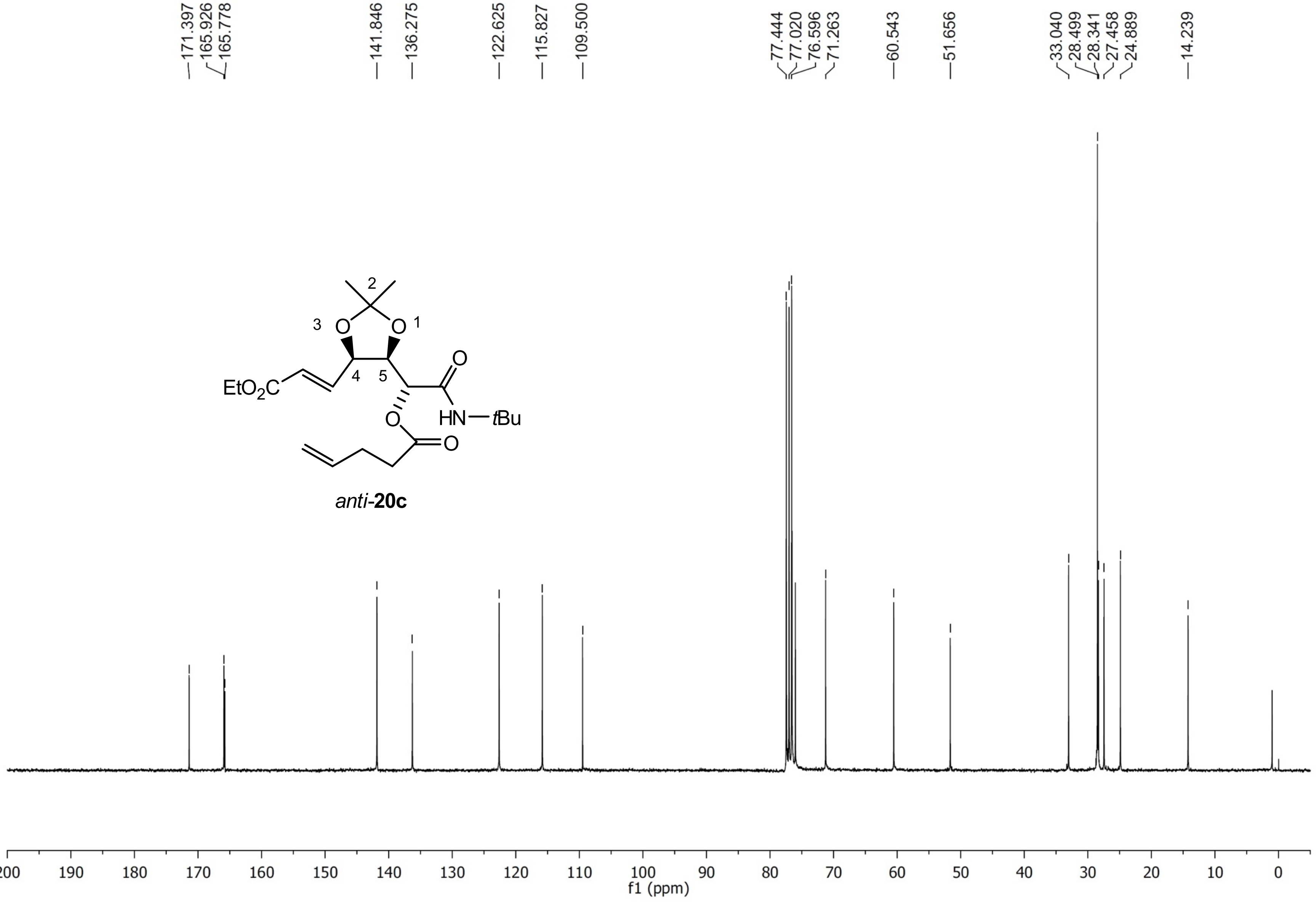
171.397
165.926
165.778
141.846
136.275
122.625
115.827
109.500
77.444
77.020
76.596
71.263
60.543
51.656
33.040
28.499
28.341
27.458
24.889
14.239
2
3
1
4
5
O
O
O
O
O
EtO2C
HN—tBu
anti-20c
200
190
180
170
160
150
140
130
120
110
100
90
80
70
60
50
40
30
20
10
0
f1 (ppm)

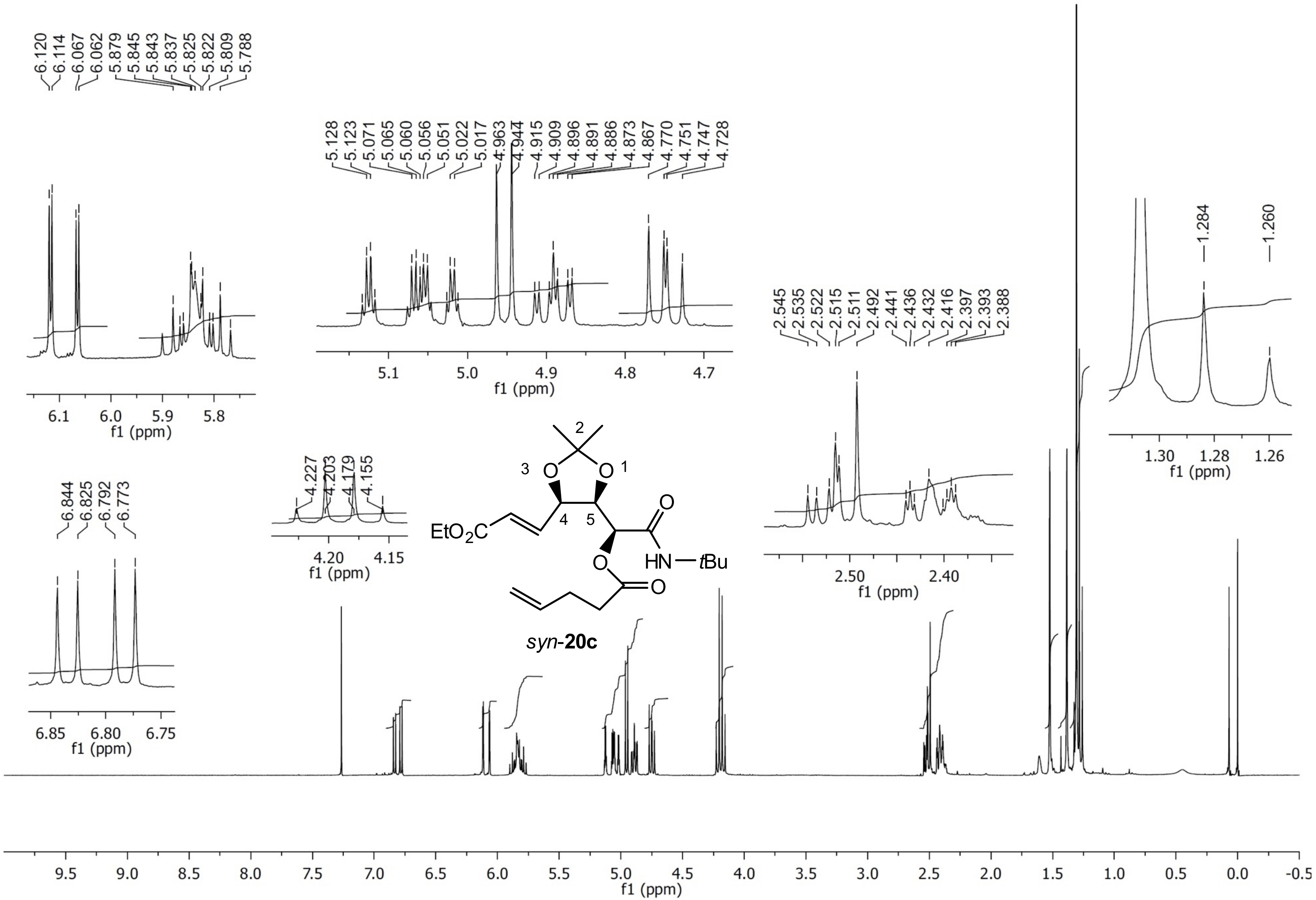
6.120
6.114
6.067
6.062
5.879
5.845
5.843
5.837
5.825
5.822
5.809
5.788
5.128
5.123
5.071
5.065
5.060
5.056
5.051
5.022
5.017
4.963
4.944
4.915
4.909
4.896
4.891
4.886
4.873
4.867
4.770
4.751
4.747
4.728
1.284
1.260
2.545
2.535
2.522
2.515
2.511
2.492
2.441
2.436
2.432
2.416
2.397
2.393
2.388
6.844
6.825
6.792
6.773
4.227
4.203
4.179
4.155
f1 (ppm)
EtO2C
HN—tBu
syn-20c

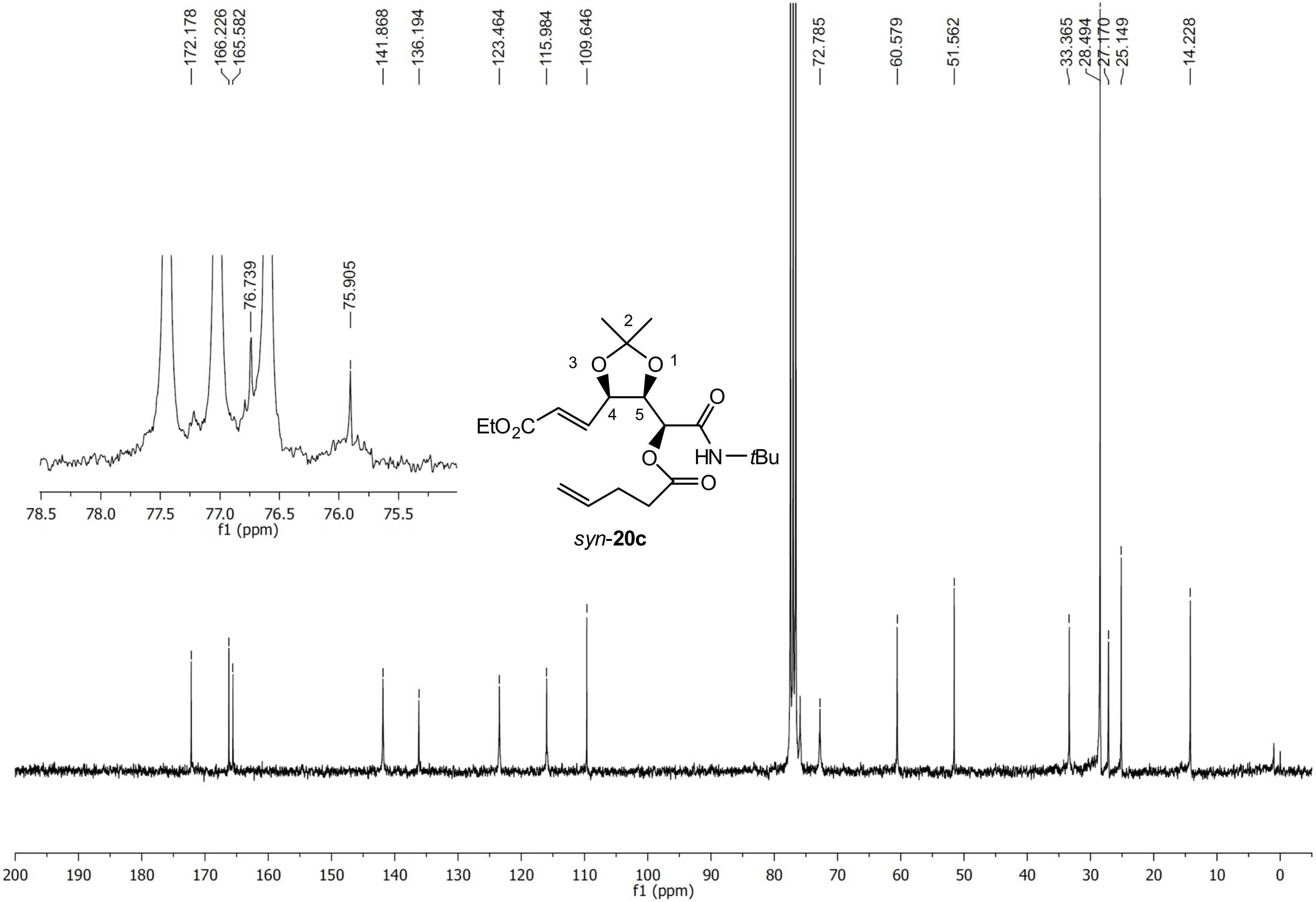

172.178
166.226
165.582
141.868
136.194
123.464
115.984
109.646
72.785
60.579
51.562
33.365
28.494
27.170
25.149
14.228
76.739
75.905
78.5 78.0 77.5 77.0 76.5 76.0 75.5
f1 (ppm)
2
3
1
O
O
O
EtO2C
4
5
HN—tBu
O
O
200 190 180 170 160 150 140 130 120 110 100 90 80 70 60 50 40 30 20 10 0
f1 (ppm)

syn-**20c**

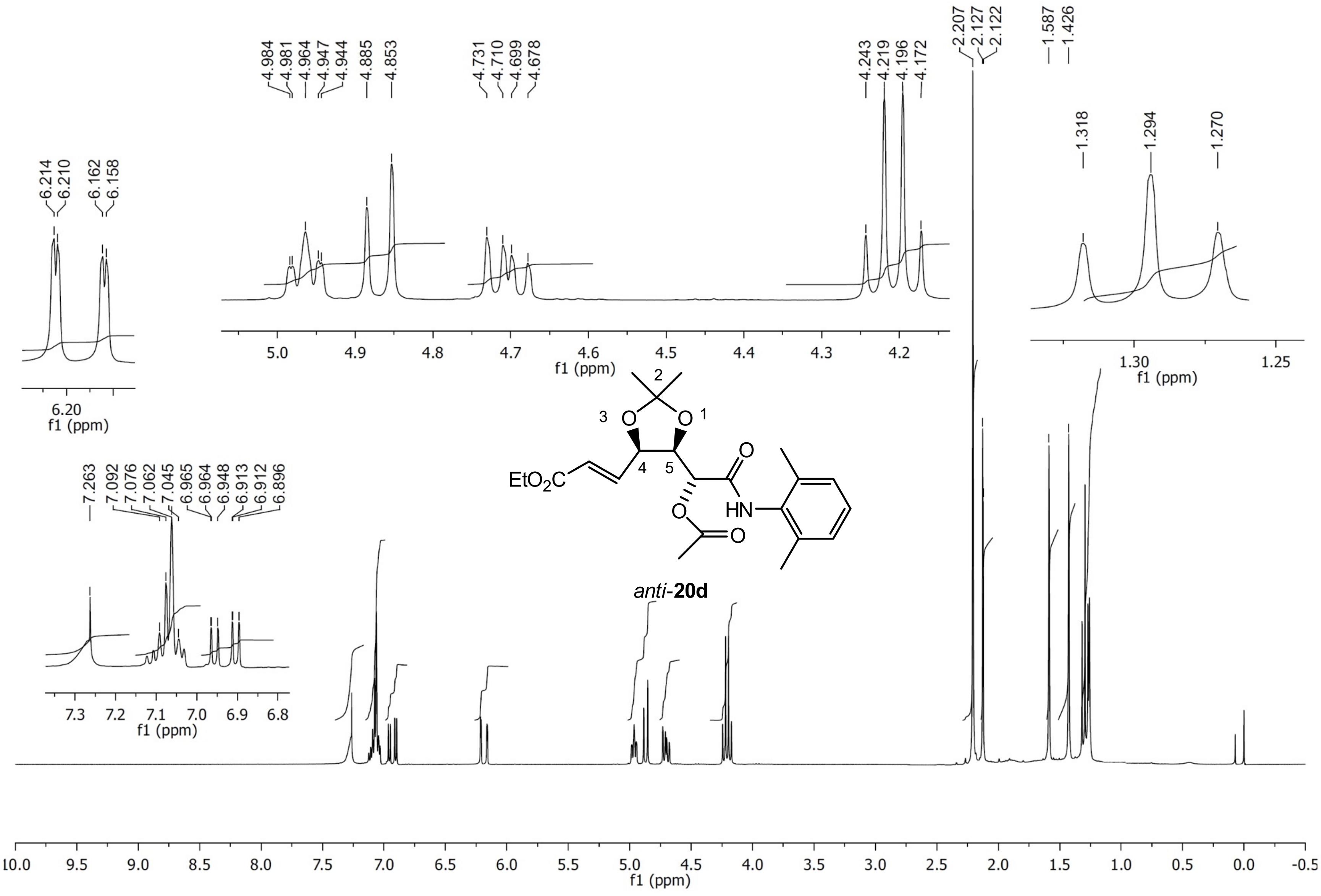
anti-20d
EtO2C
HN
f1 (ppm)

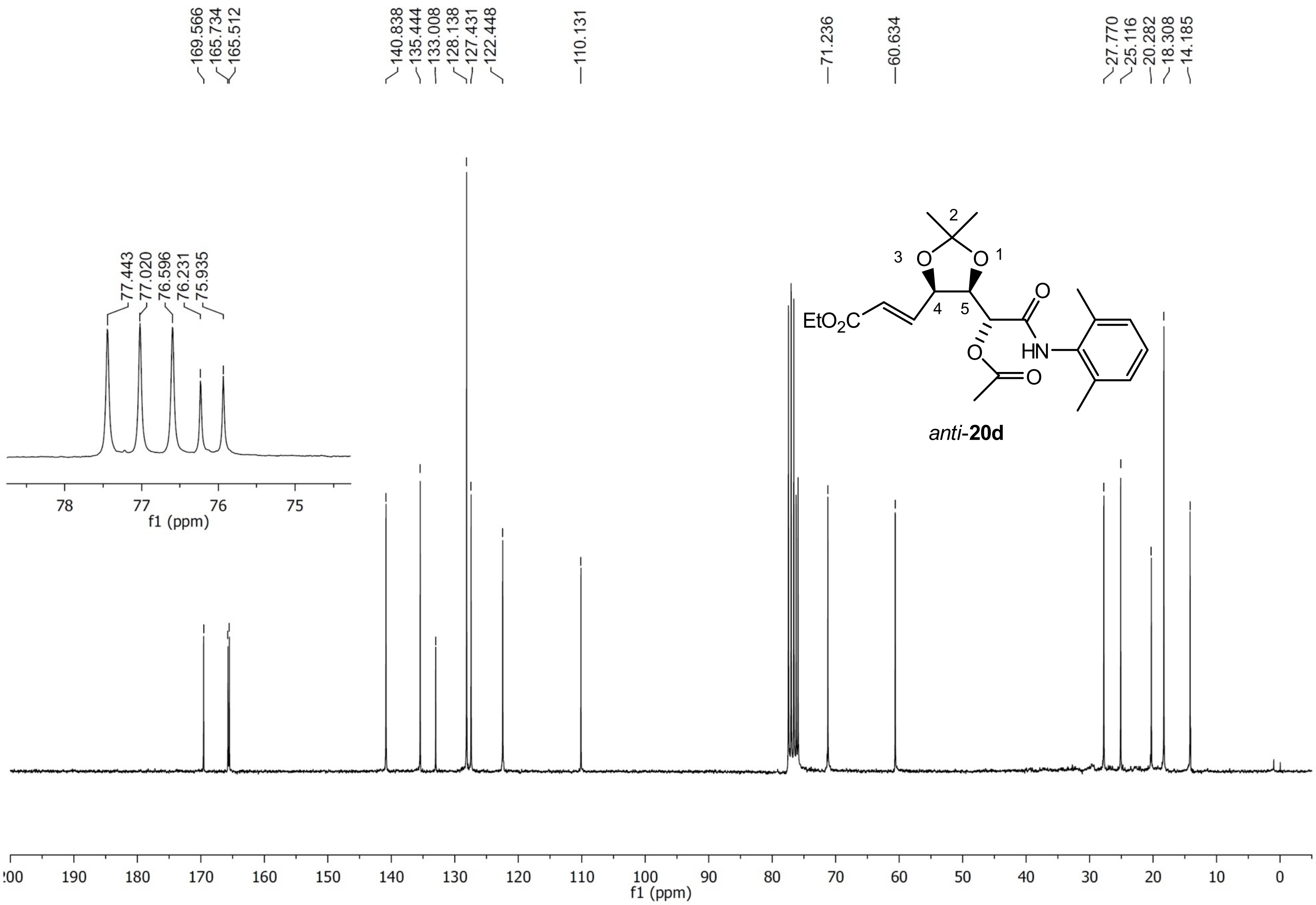
169.566
165.734
165.512
140.838
135.444
133.008
128.138
127.431
122.448
110.131
71.236
60.634
27.770
25.116
20.282
18.308
14.185
77.443
77.020
76.596
76.231
75.935
78
77
76
75
f1 (ppm)
EtO2C
HN
O
2
3
1
4
5
anti-20d
200
190
180
170
160
150
140
130
120
110
100
90
80
70
60
50
40
30
20
10
0
f1 (ppm)

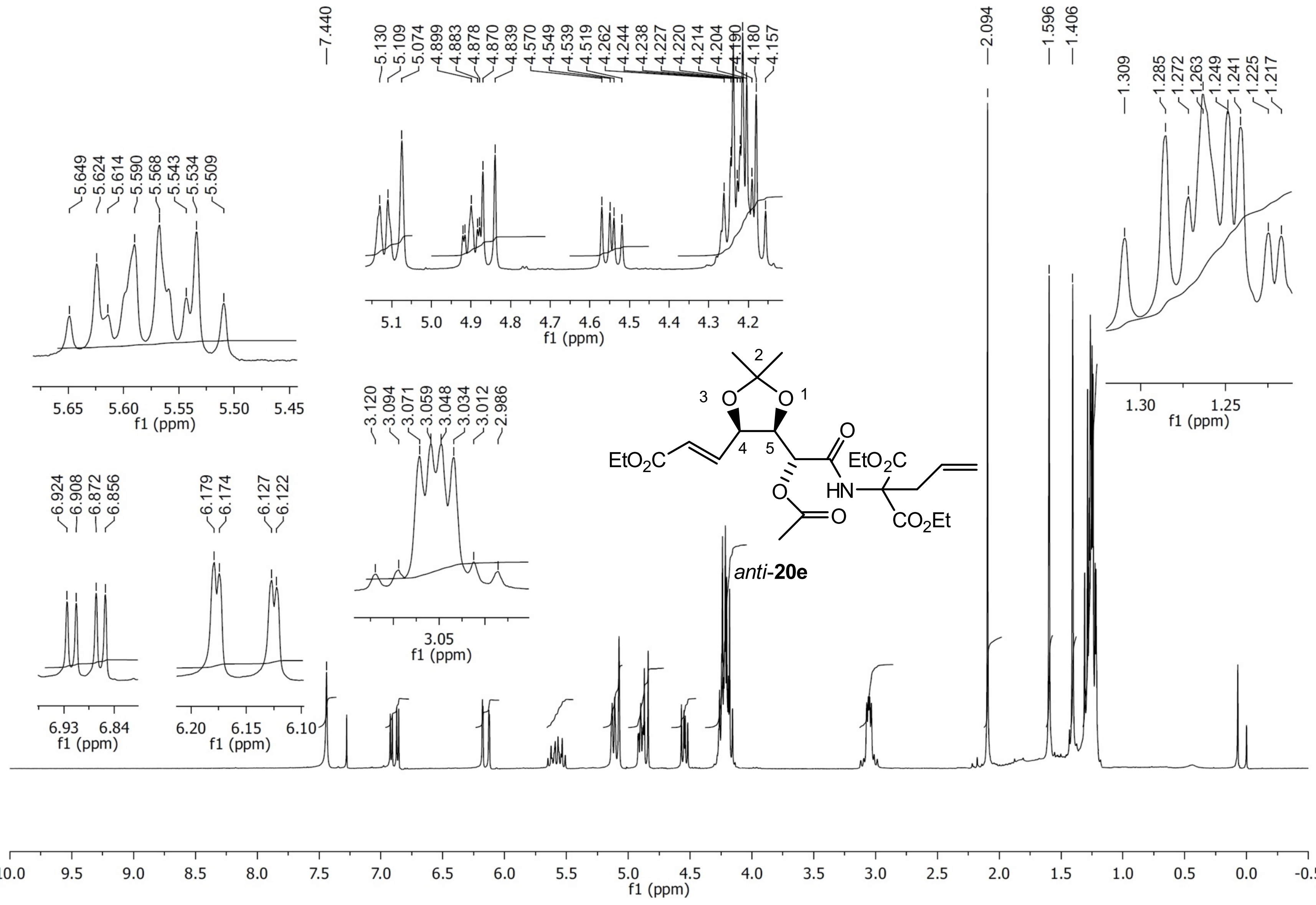
anti-20e
EtO2C
EtO2C
HN
CO2Et
f1 (ppm)

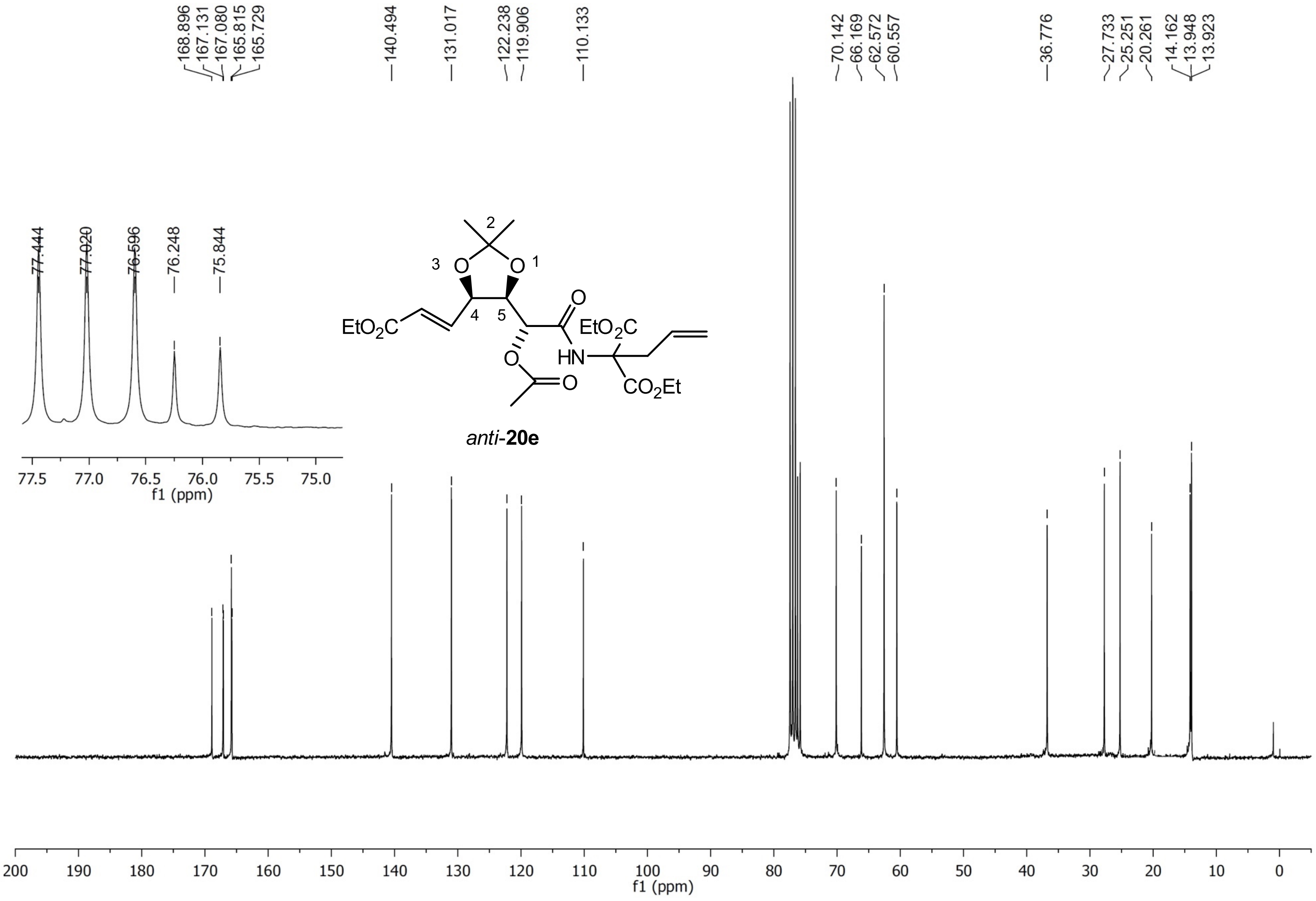
168.896
167.131
167.080
165.815
165.729
140.494
131.017
122.238
119.906
110.133
70.142
66.169
62.572
60.557
36.776
27.733
25.251
20.261
14.162
13.948
13.923
77.444
77.020
76.596
76.248
75.844
f1 (ppm)
EtO2C
EtO2C
HN
CO2Et
anti-20e

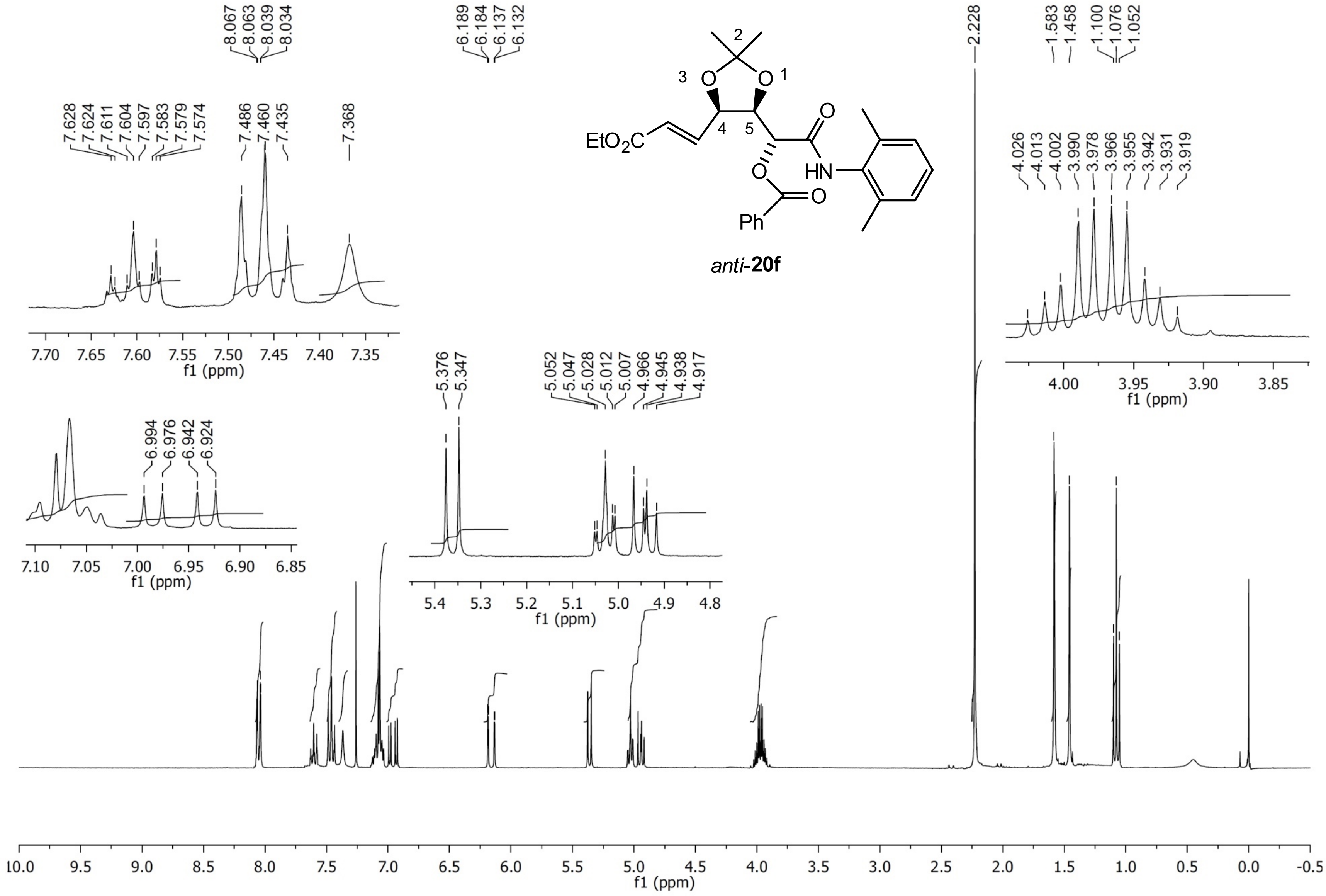

anti-20f
EtO2C
Ph
HN
f1 (ppm)

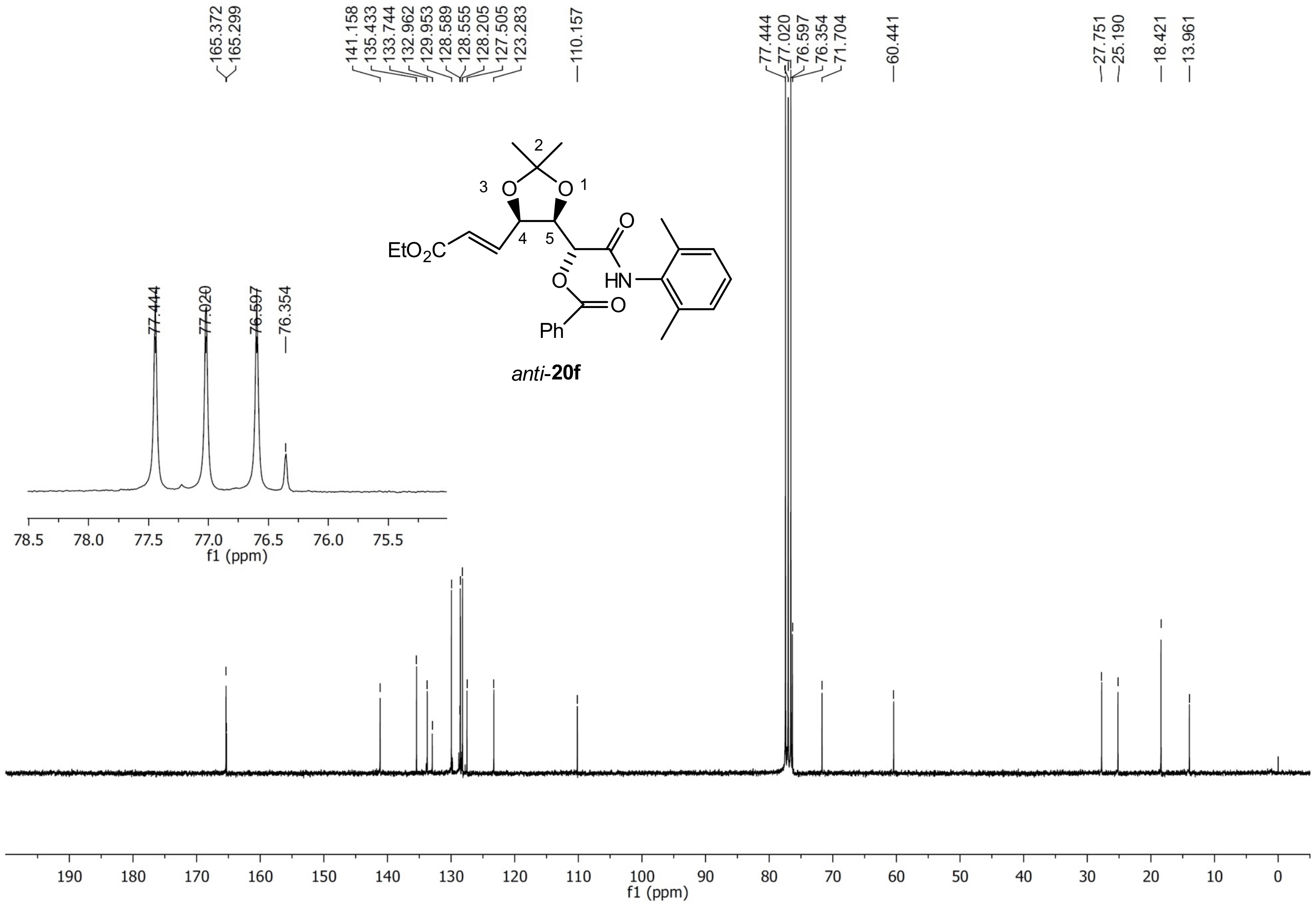

165.372
165.299
141.158
135.433
133.744
132.962
129.953
128.589
128.555
128.205
127.505
123.283
110.157
77.444
77.020
76.597
76.354
71.704
60.441
27.751
25.190
18.421
13.961
EtO2C
O
O
2
3
1
4
5
HN
O
Ph
anti-20f
78.5 78.0 77.5 77.0 76.5 76.0 75.5
f1 (ppm)
190 180 170 160 150 140 130 120 110 100 90 80 70 60 50 40 30 20 10 0
f1 (ppm)

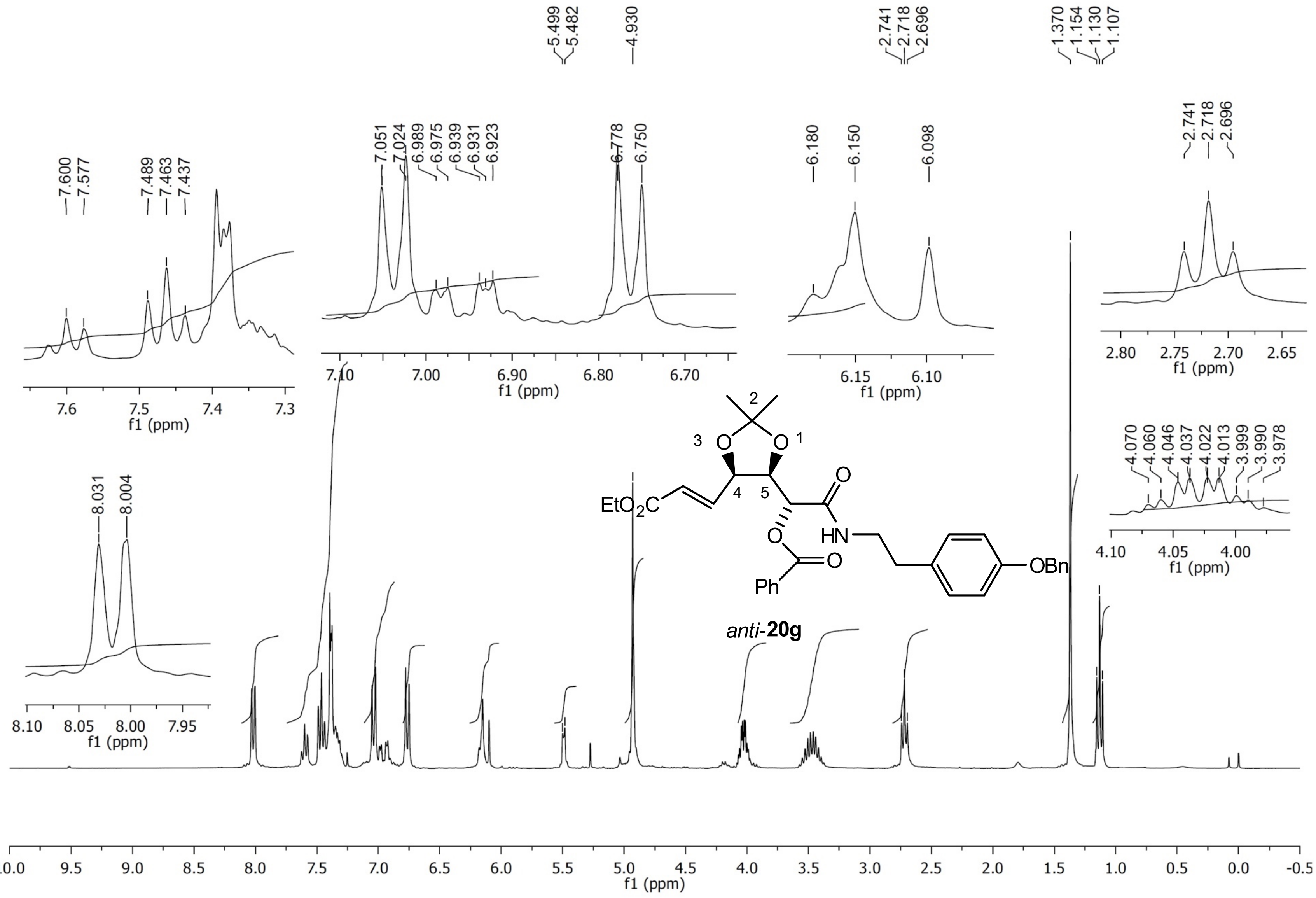

anti-20g
EtO2C
O
HN
OBn
Ph
f1 (ppm)

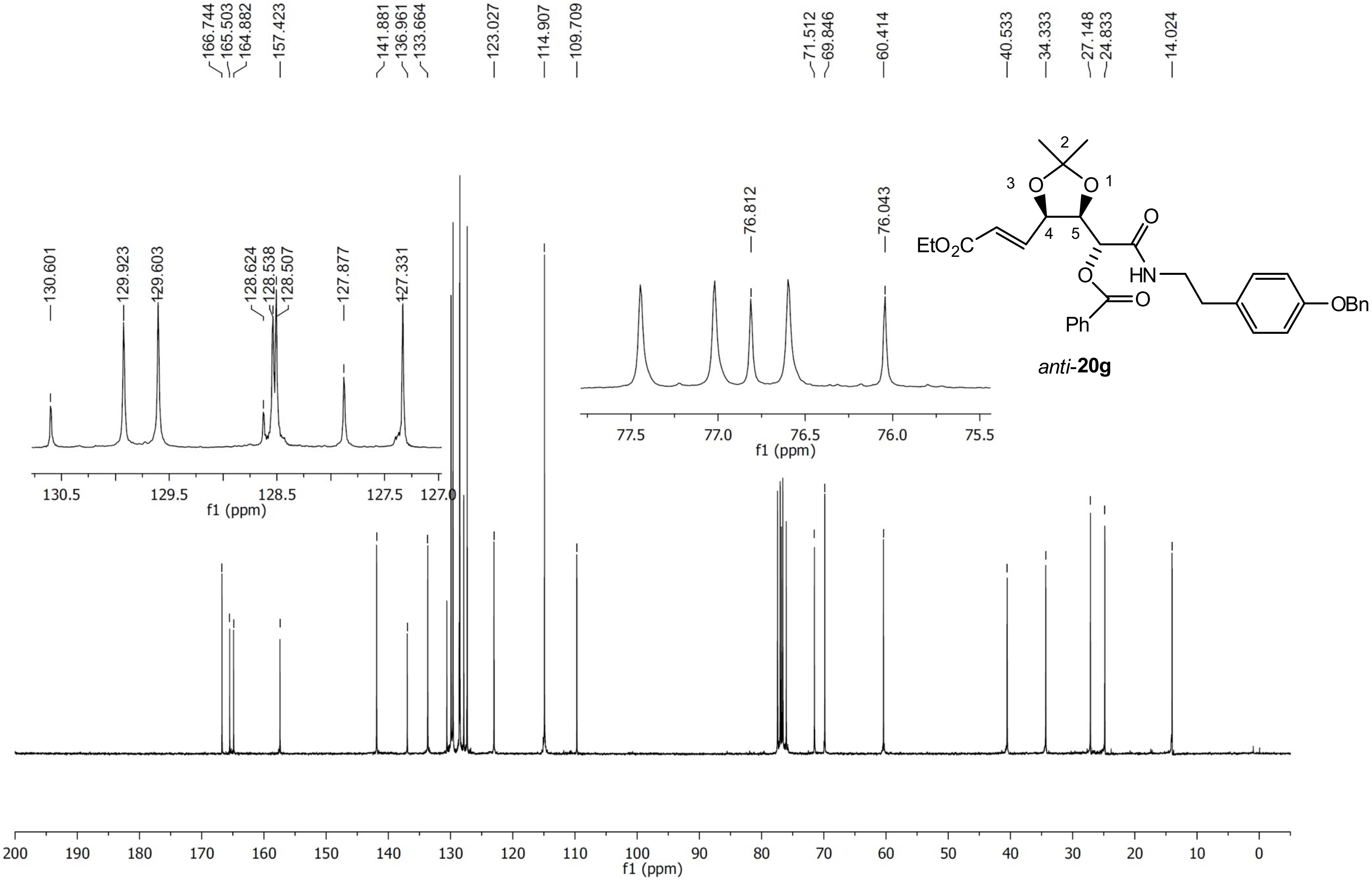
166.744
165.503
164.882
157.423
141.881
136.961
133.664
123.027
114.907
109.709
71.512
69.846
60.414
40.533
34.333
27.148
24.833
14.024
130.601
129.923
129.603
128.624
128.538
128.507
127.877
127.331
76.812
76.043
EtO2C
HN
OBn
Ph
f1 (ppm)

anti-**20g**

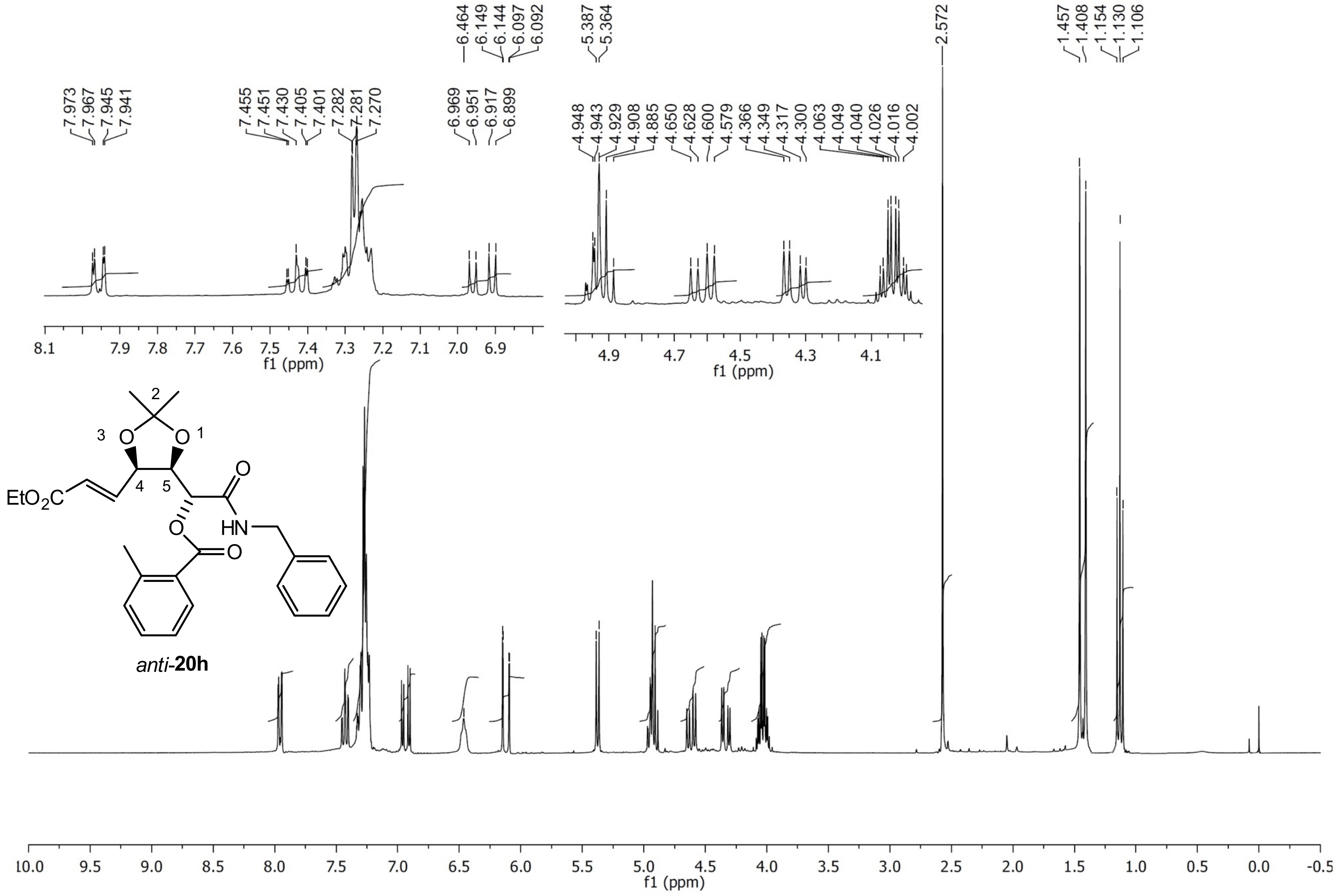
7.973
7.967
7.945
7.941
7.455
7.451
7.430
7.405
7.401
7.282
7.281
7.270
6.969
6.951
6.917
6.899
6.464
6.149
6.144
6.097
6.092
5.387
5.364
4.948
4.943
4.929
4.908
4.885
4.650
4.628
4.600
4.579
4.366
4.349
4.317
4.300
4.063
4.049
4.040
4.026
4.016
4.002
2.572
1.457
1.408
1.154
1.130
1.106
f1 (ppm)
f1 (ppm)
f1 (ppm)
EtO2C
HN
anti-20h

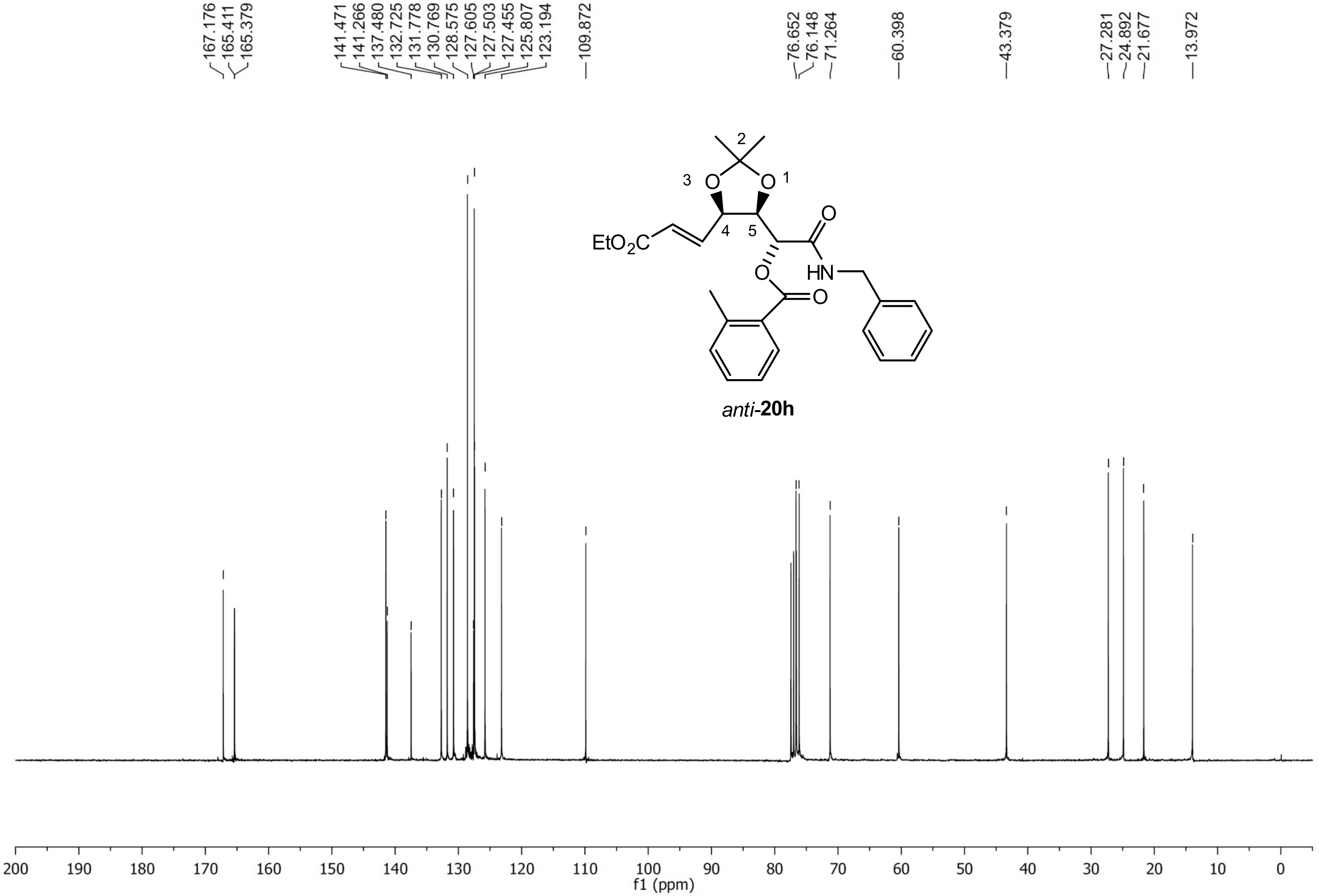
167.176
165.411
165.379
141.471
141.266
137.480
132.725
131.778
130.769
128.575
127.605
127.503
127.455
125.807
123.194
109.872
76.652
76.148
71.264
60.398
43.379
27.281
24.892
21.677
13.972
EtO2C
HN
O
2
3
1
4
5
200
190
180
170
160
150
140
130
120
110
100
90
80
70
60
50
40
30
20
10
0
f1 (ppm)

anti-**20h**

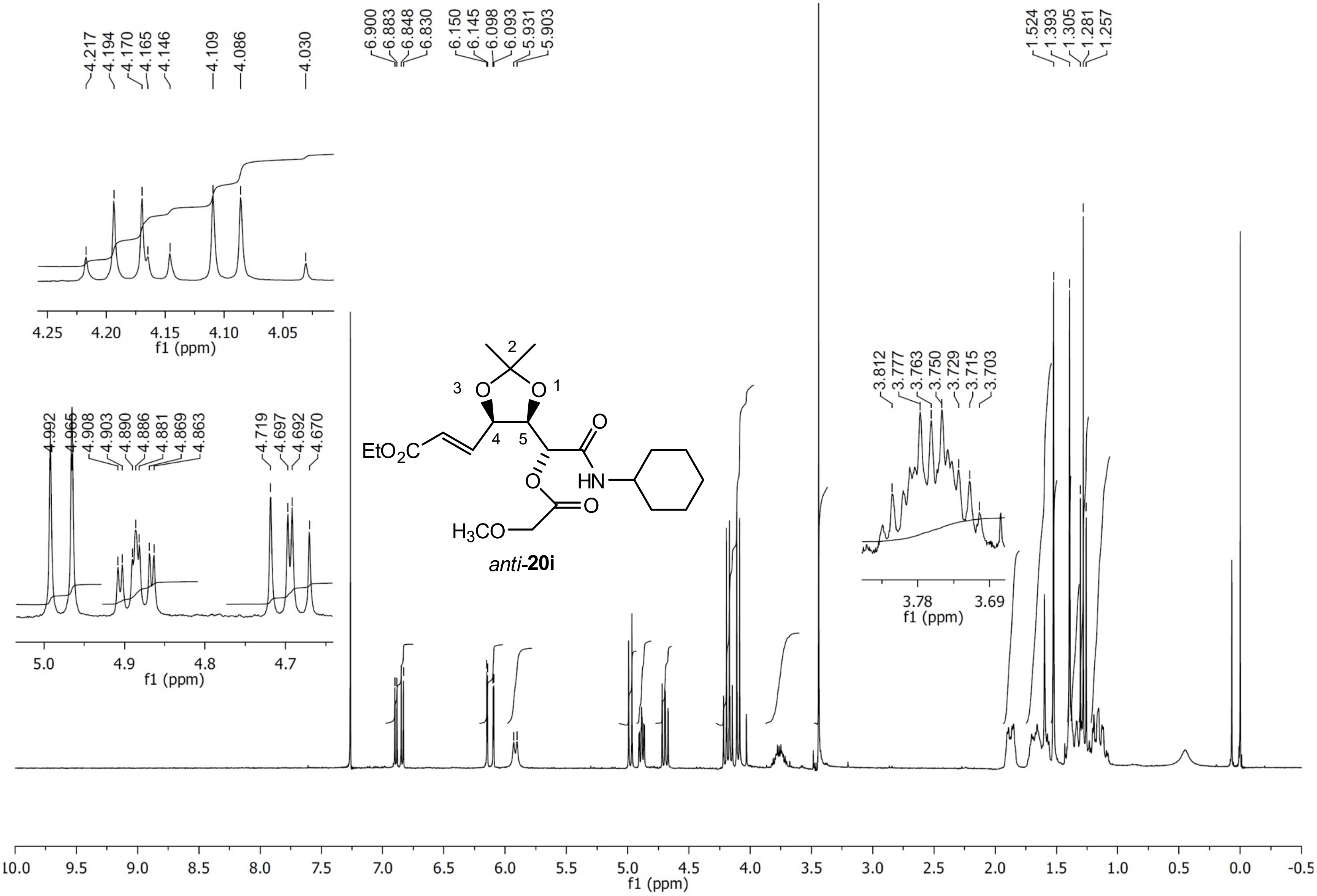
4.217
4.194
4.170
4.165
4.146
4.109
4.086
4.030
6.900
6.883
6.848
6.830
6.150
6.145
6.098
6.093
5.931
5.903
1.524
1.393
1.305
1.281
1.257
f1 (ppm)
4.992
4.965
4.908
4.903
4.890
4.886
4.881
4.869
4.863
4.719
4.697
4.692
4.670
3.812
3.777
3.763
3.750
3.729
3.715
3.703
EtO2C
HN
H3CO

anti-**20i**

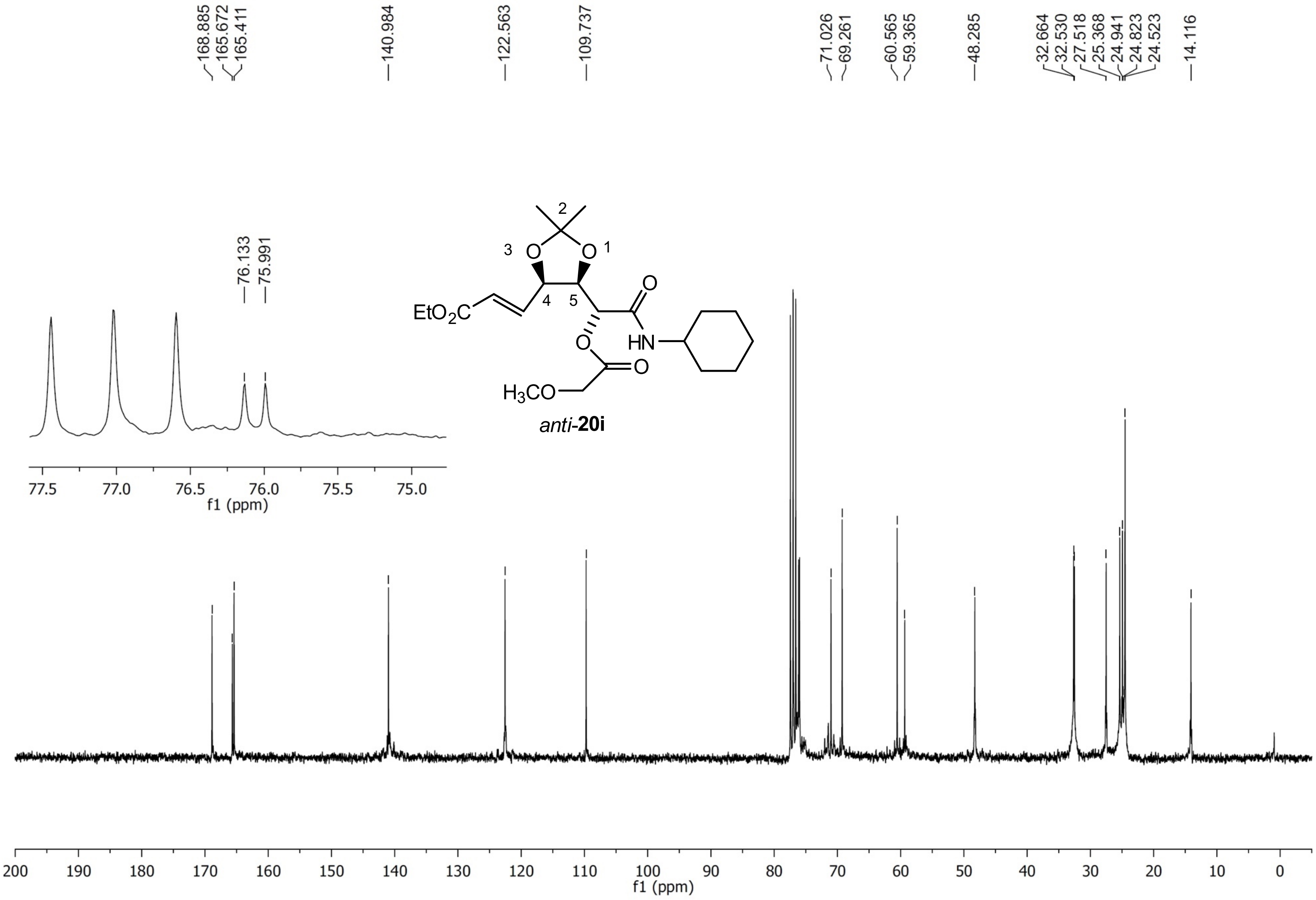
168.885
165.672
165.411
140.984
122.563
109.737
71.026
69.261
60.565
59.365
48.285
32.664
32.530
27.518
25.368
24.941
24.823
24.523
14.116
76.133
75.991
77.5 77.0 76.5 76.0 75.5 75.0
f1 (ppm)
EtO2C
O
HN
H3CO
anti-20i
200 190 180 170 160 150 140 130 120 110 100 90 80 70 60 50 40 30 20 10 0
f1 (ppm)

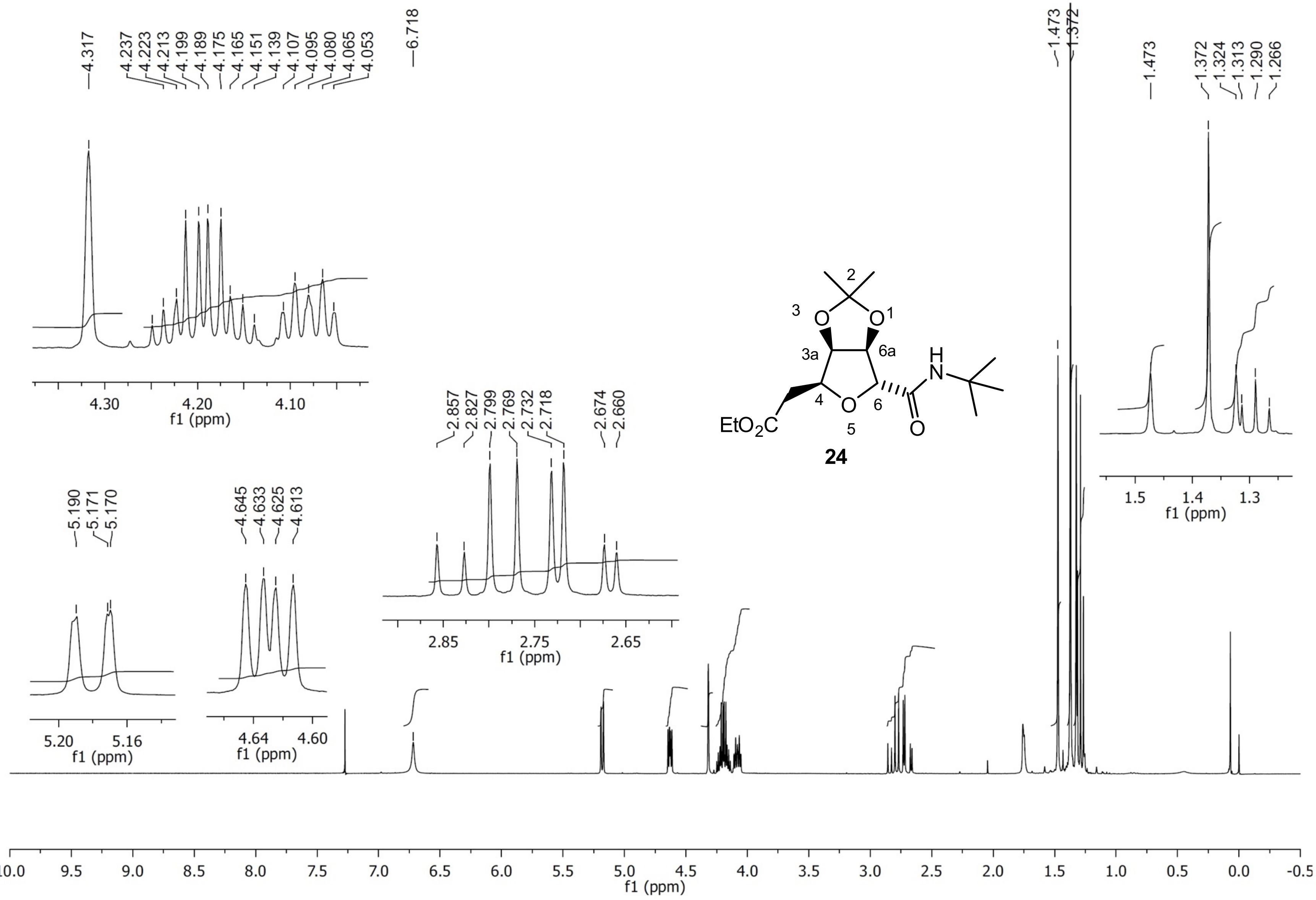

4.317
4.237
4.223
4.213
4.199
4.189
4.175
4.165
4.151
4.139
4.107
4.095
4.080
4.065
4.053
6.718
1.473
1.372
1.324
1.313
1.290
1.266
2.857
2.827
2.799
2.769
2.732
2.718
2.674
2.660
5.190
5.171
5.170
4.645
4.633
4.625
4.613
f1 (ppm)
EtO2C
24

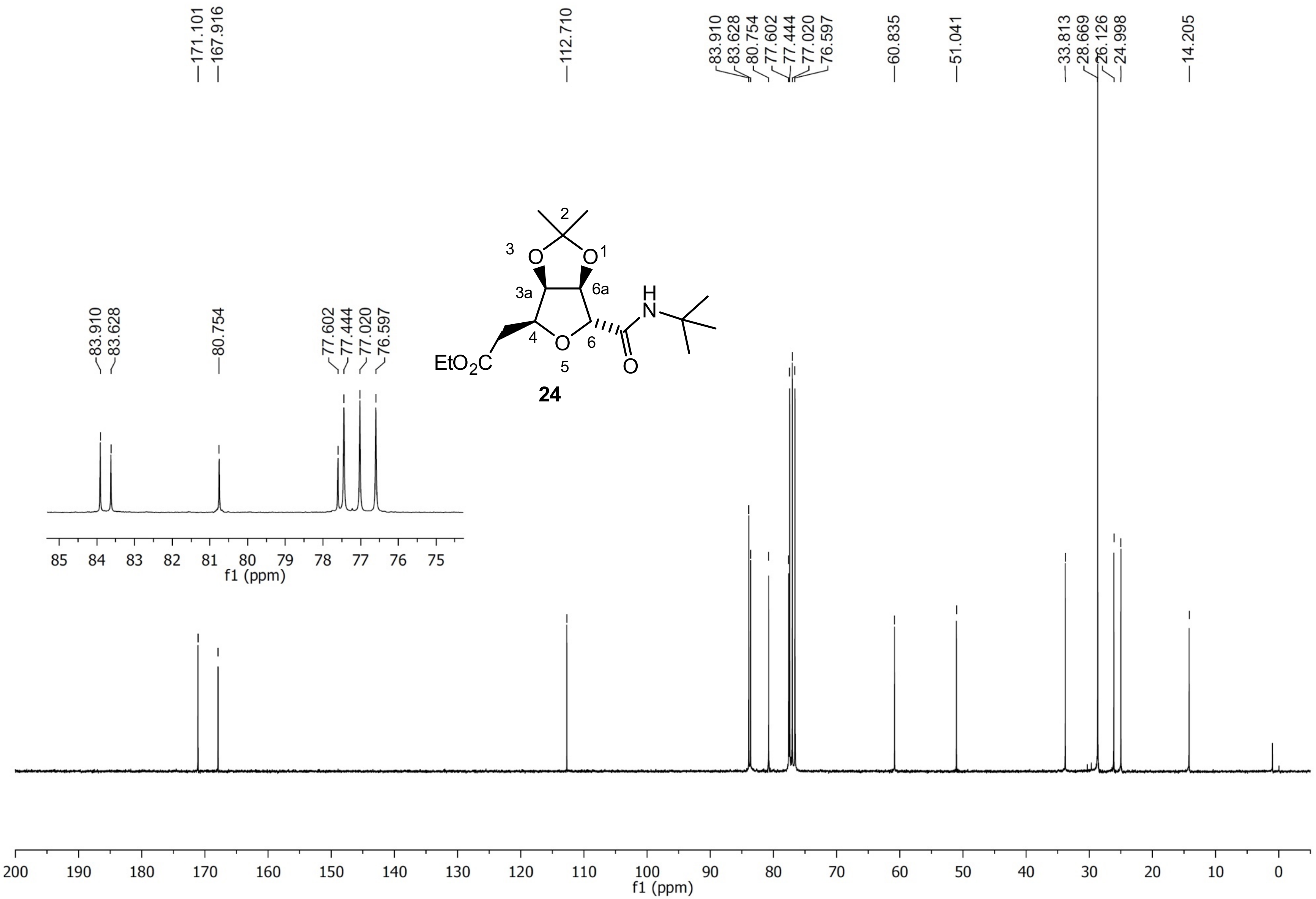
171.101
167.916
112.710
83.910
83.628
80.754
77.602
77.444
77.020
76.597
60.835
51.041
33.813
28.669
26.126
24.998
14.205
EtO2C
24
f1 (ppm)

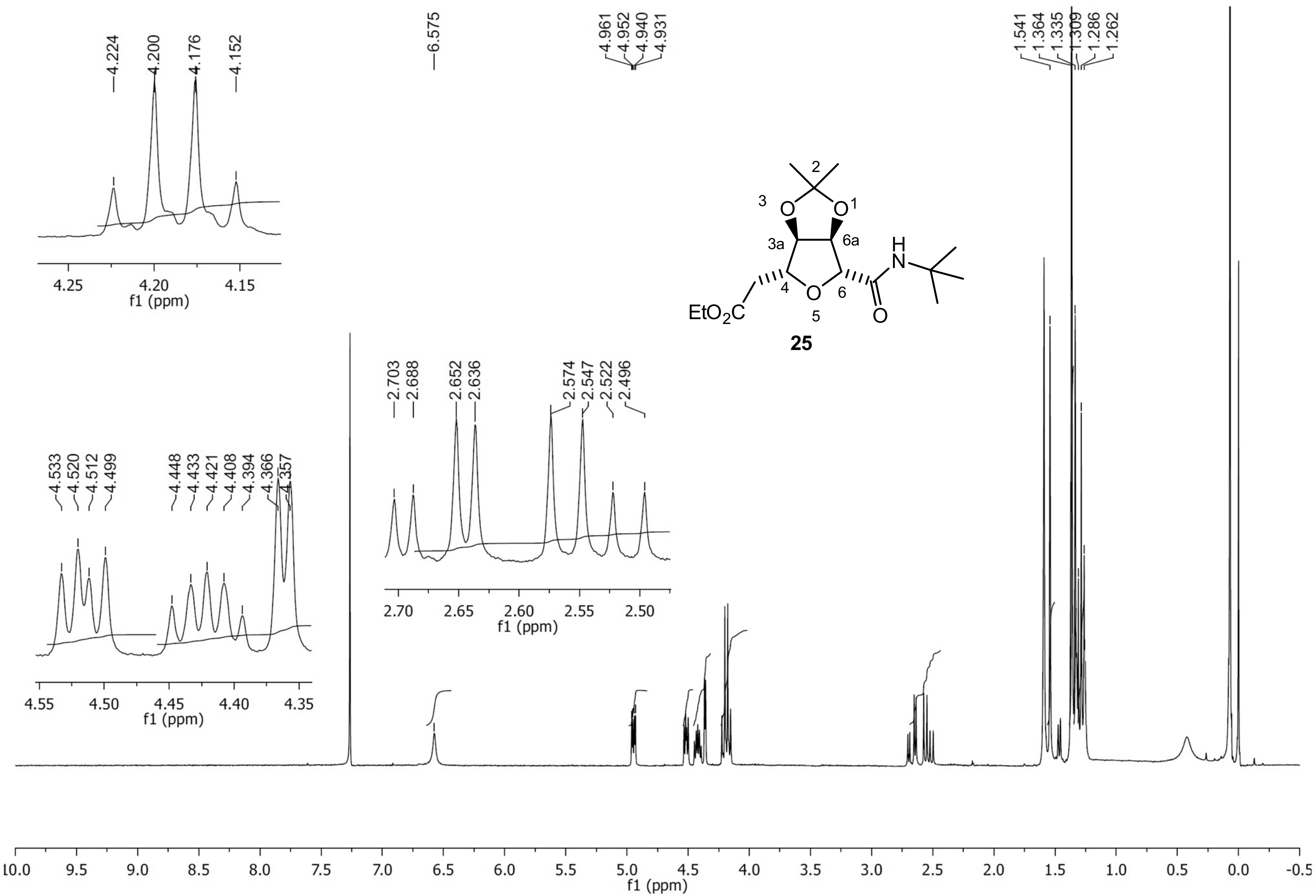
4.224
4.200
4.176
4.152
4.25 4.20 4.15
f1 (ppm)
6.575
4.961
4.952
4.940
4.931
1.541
1.364
1.335
1.309
1.286
1.262
2
3
O
O1
3a
6a
H
N
4
O
6
5
EtO2C
O
25
2.703
2.688
2.652
2.636
2.574
2.547
2.522
2.496
2.70 2.65 2.60 2.55 2.50
f1 (ppm)
4.533
4.520
4.512
4.499
4.448
4.433
4.421
4.408
4.394
4.366
4.357
4.55 4.50 4.45 4.40 4.35
f1 (ppm)
10.0 9.5 9.0 8.5 8.0 7.5 7.0 6.5 6.0 5.5 5.0 4.5 4.0 3.5 3.0 2.5 2.0 1.5 1.0 0.5 0.0 -0.5
f1 (ppm)

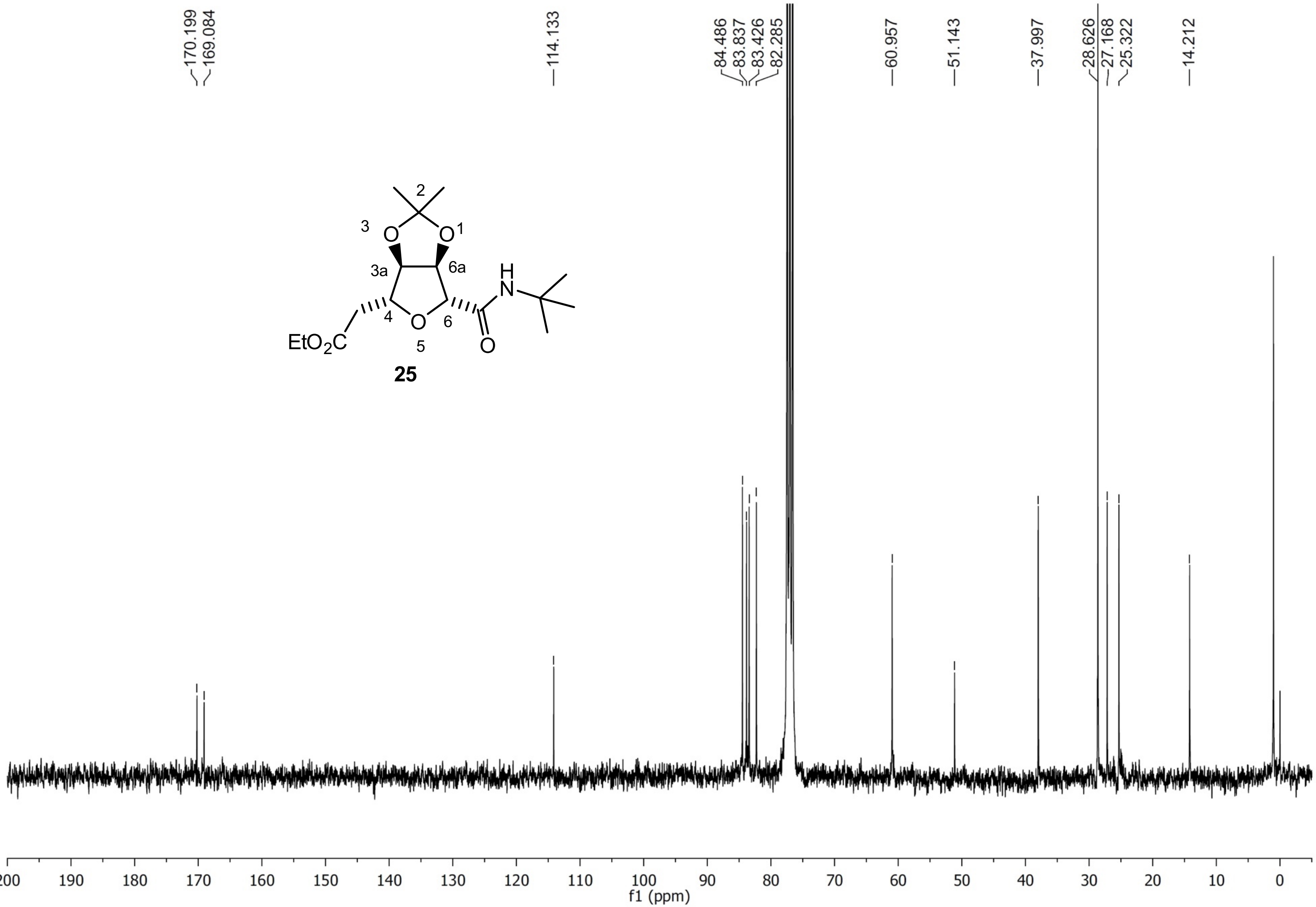
170.199
169.084
114.133
84.486
83.837
83.426
82.285
60.957
51.143
37.997
28.626
27.168
25.322
14.212
EtO2C
25
200 190 180 170 160 150 140 130 120 110 100 90 80 70 60 50 40 30 20 10 0
f1 (ppm)

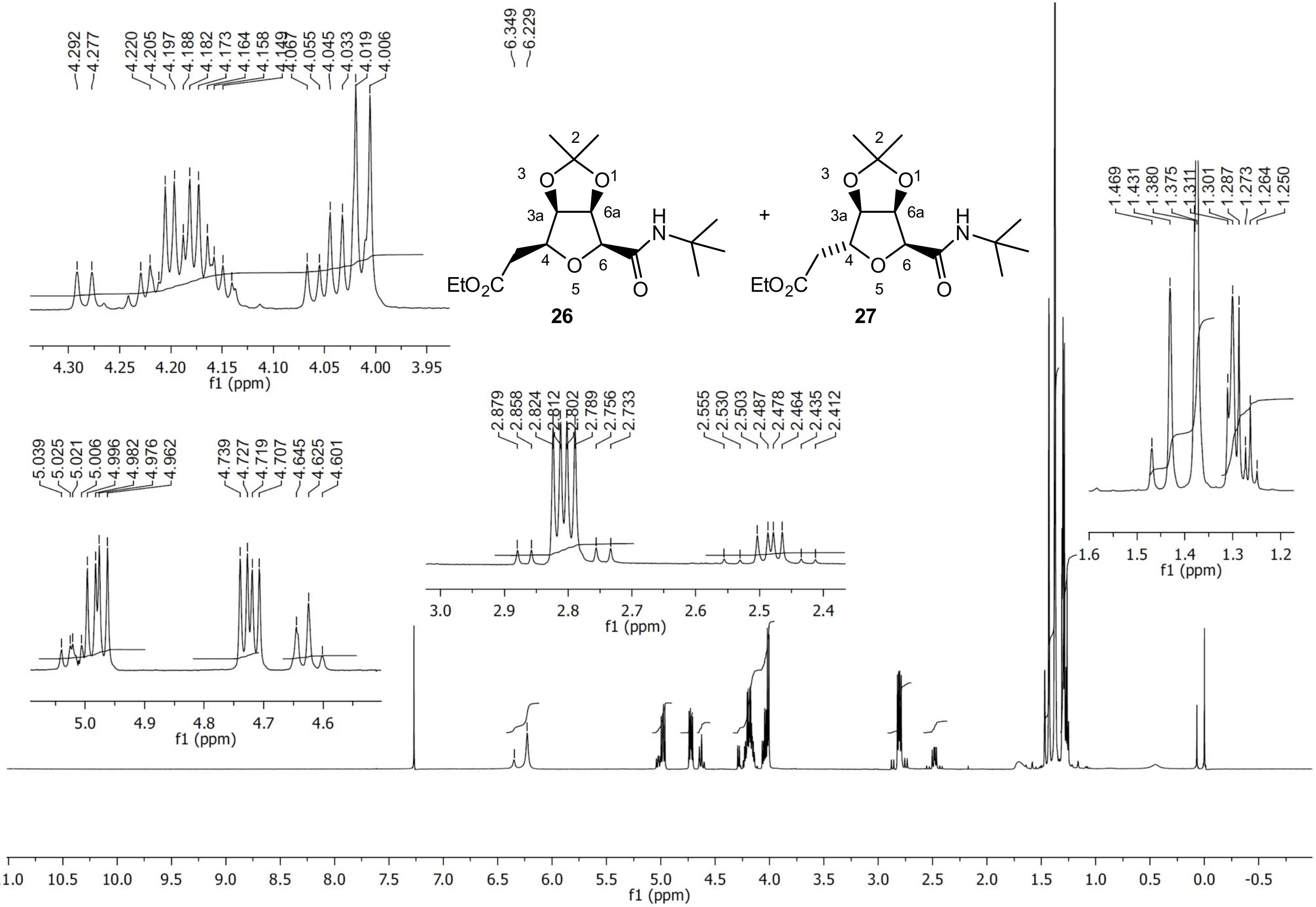

26
27
f1 (ppm)

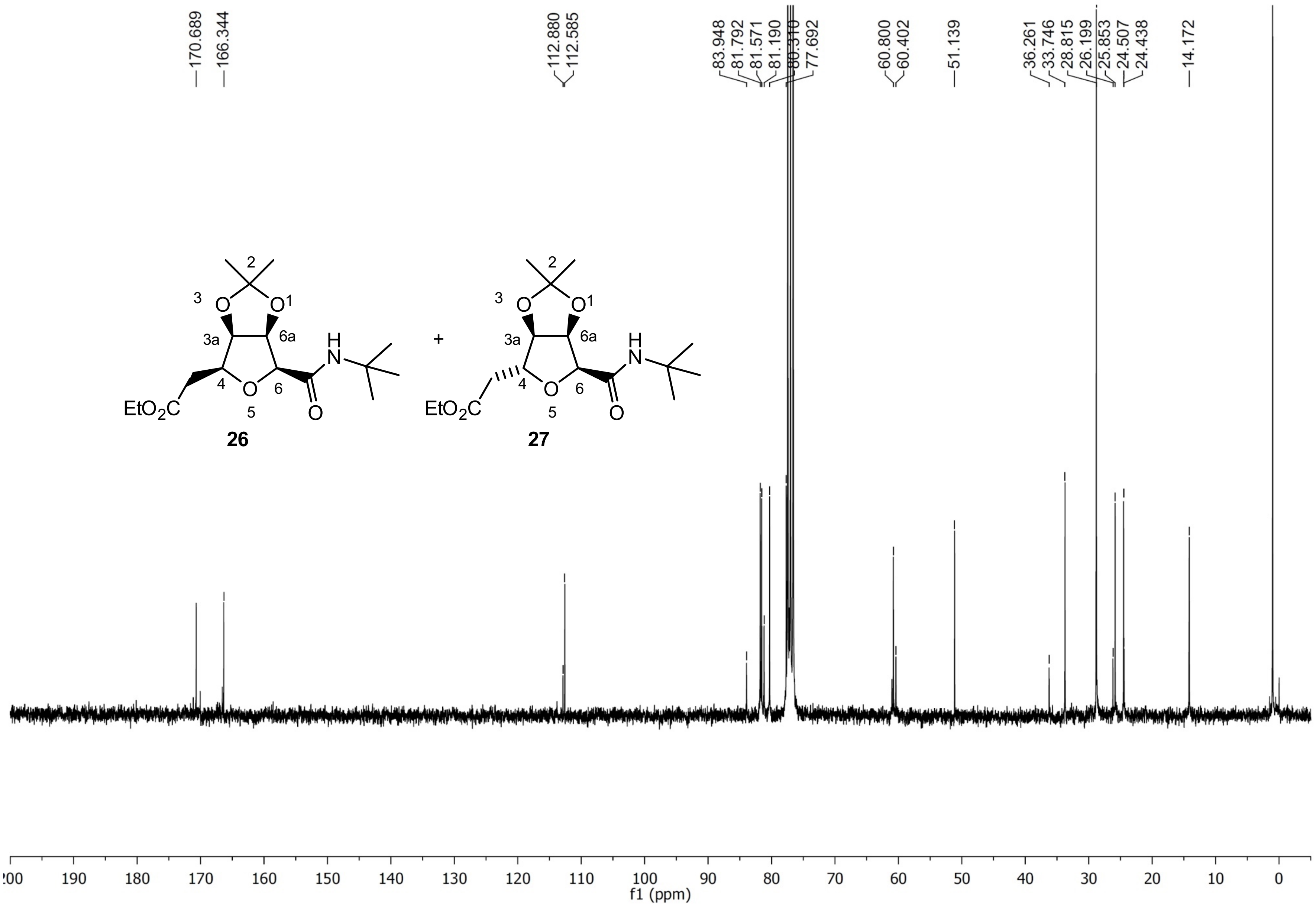
170.689
166.344
112.880
112.585
83.948
81.792
81.571
81.190
77.692
60.800
60.402
51.139
36.261
33.746
28.815
26.199
25.853
24.507
24.438
14.172
26
27
f1 (ppm)

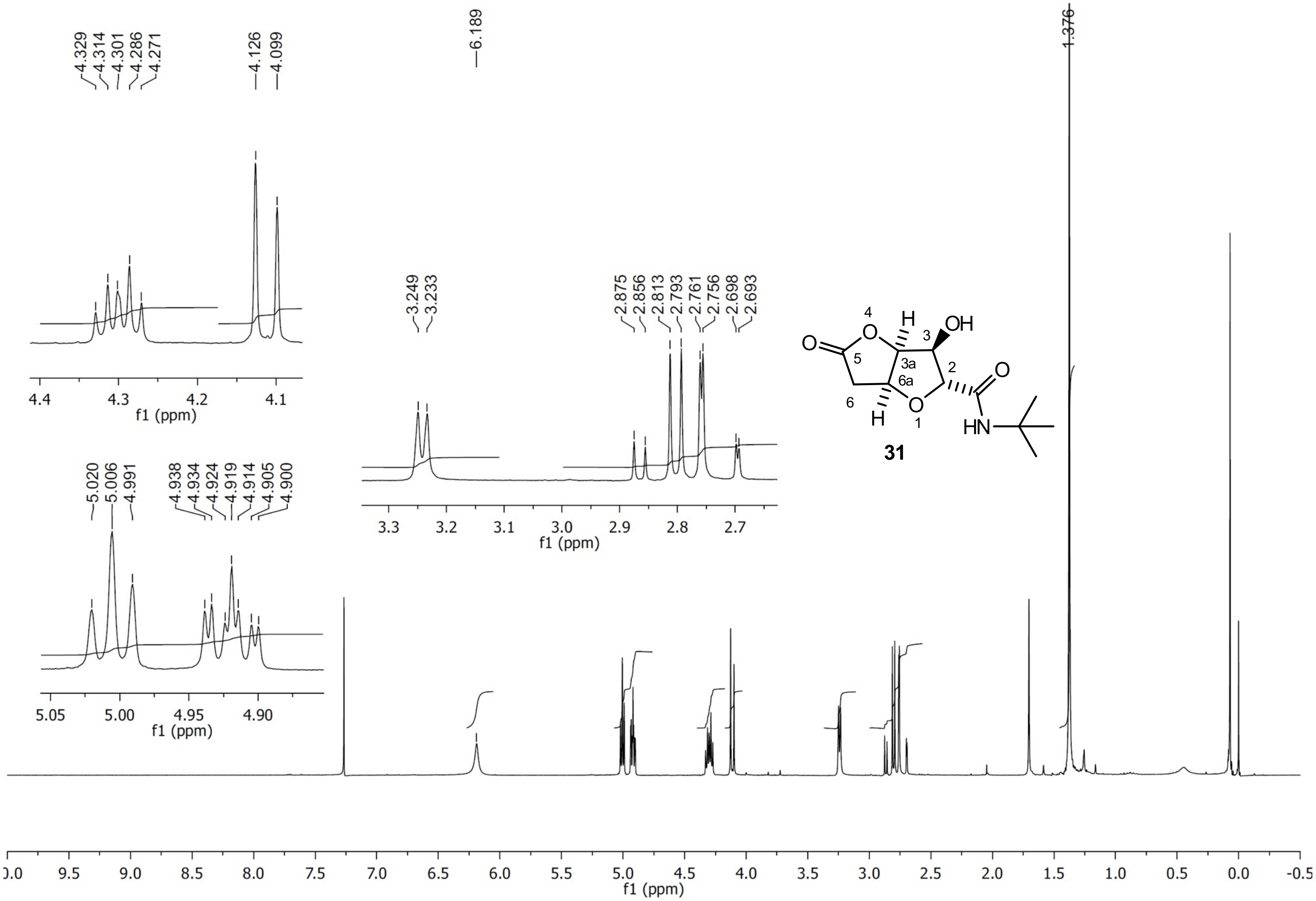

4.329
4.314
4.301
4.286
4.271
4.126
4.099
6.189
1.376
3.249
3.233
2.875
2.856
2.813
2.793
2.761
2.756
2.698
2.693
5.020
5.006
4.991
4.938
4.934
4.924
4.919
4.914
4.905
4.900
f1 (ppm)
OH
HN
31

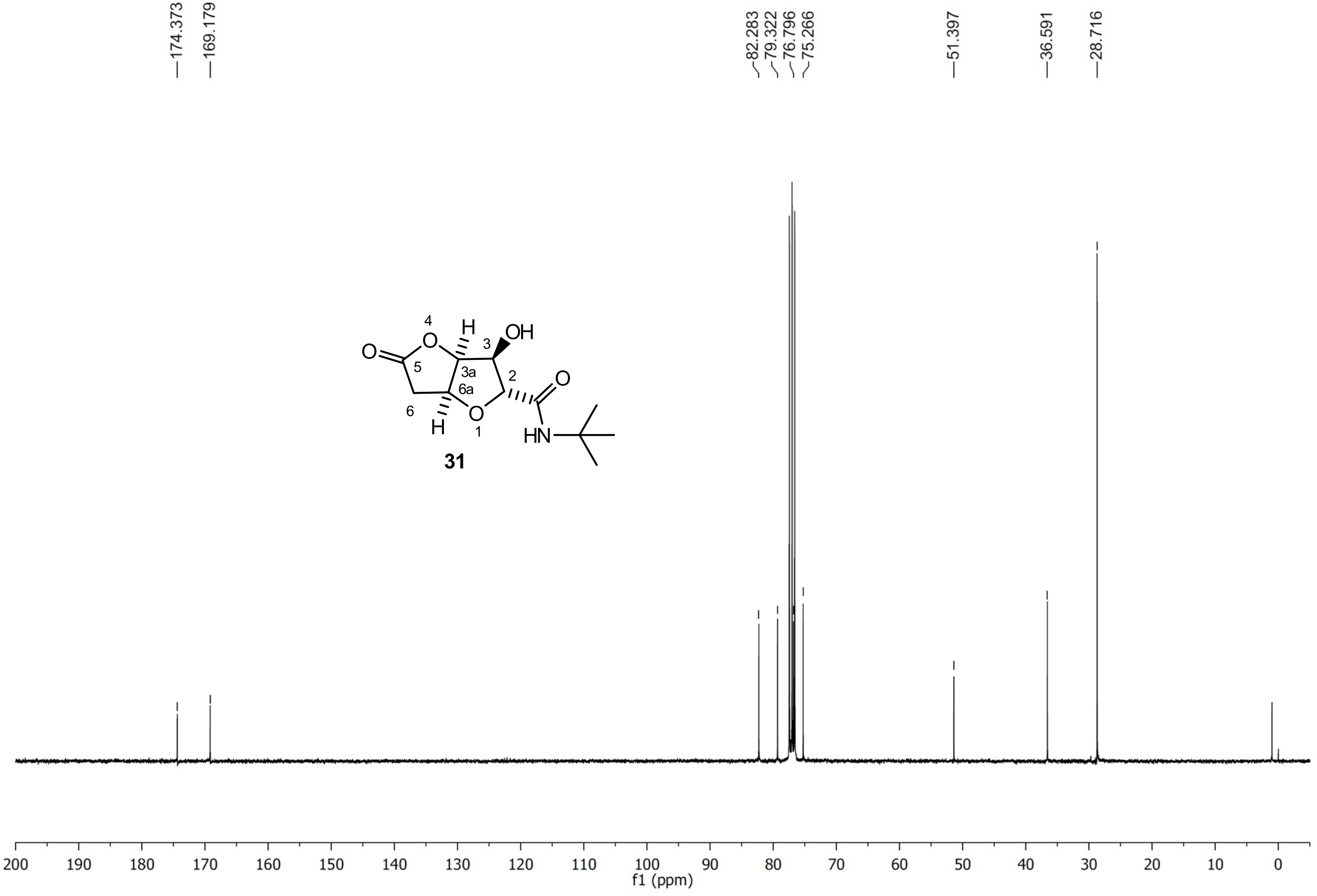
174.373
169.179
82.283
79.322
76.796
75.266
51.397
36.591
28.716
4
H
OH
3
O
5
3a
2
O
6a
6
H
O
1
HN
31
200
190
180
170
160
150
140
130
120
110
100
90
80
70
60
50
40
30
20
10
0
f1 (ppm)

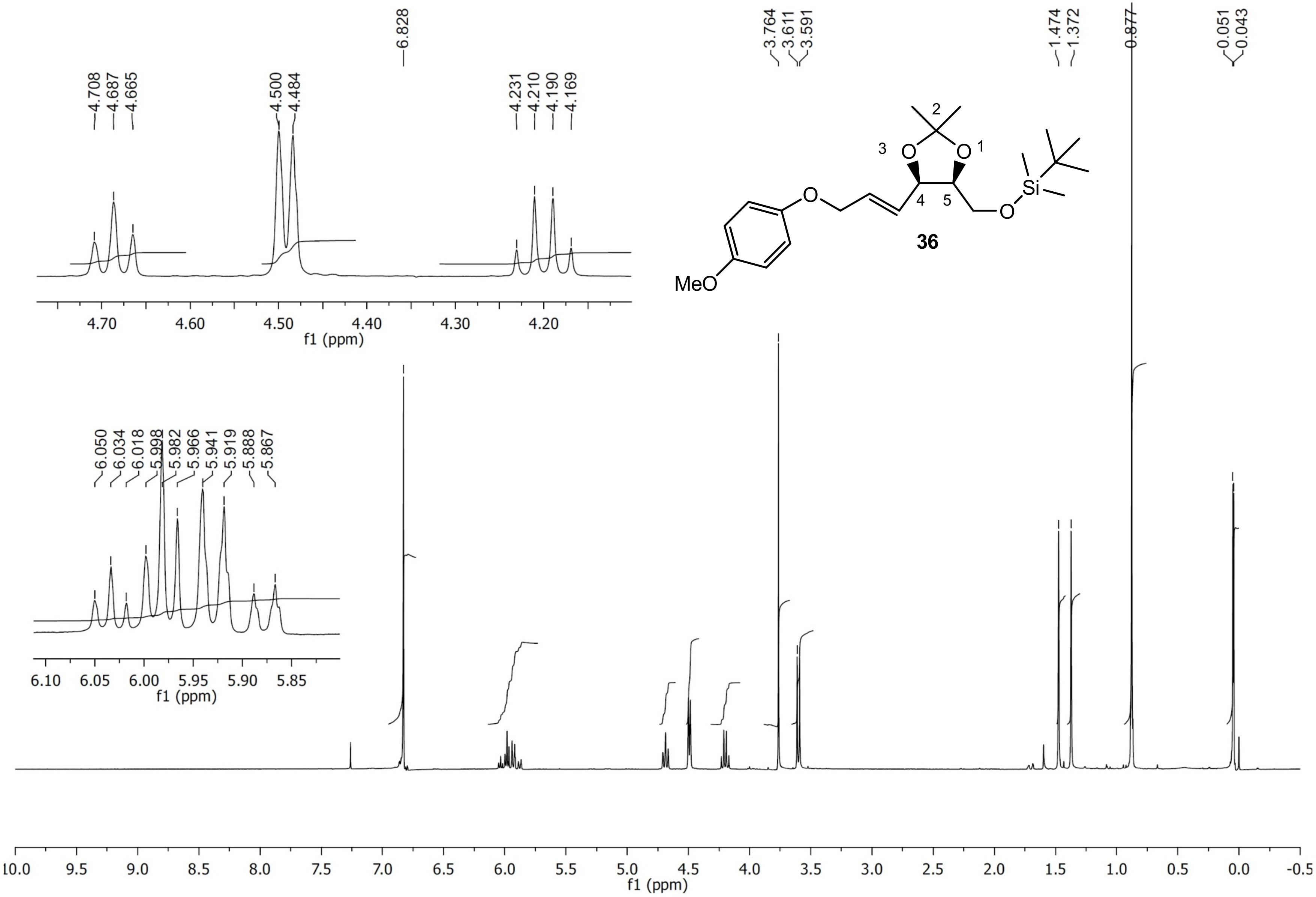
4.708
4.687
4.665
4.500
4.484
6.828
4.231
4.210
4.190
4.169
3.764
3.611
3.591
1.474
1.372
0.877
0.051
0.043
36
MeO
6.050
6.034
6.018
5.998
5.982
5.966
5.941
5.919
5.888
5.867
f1 (ppm)

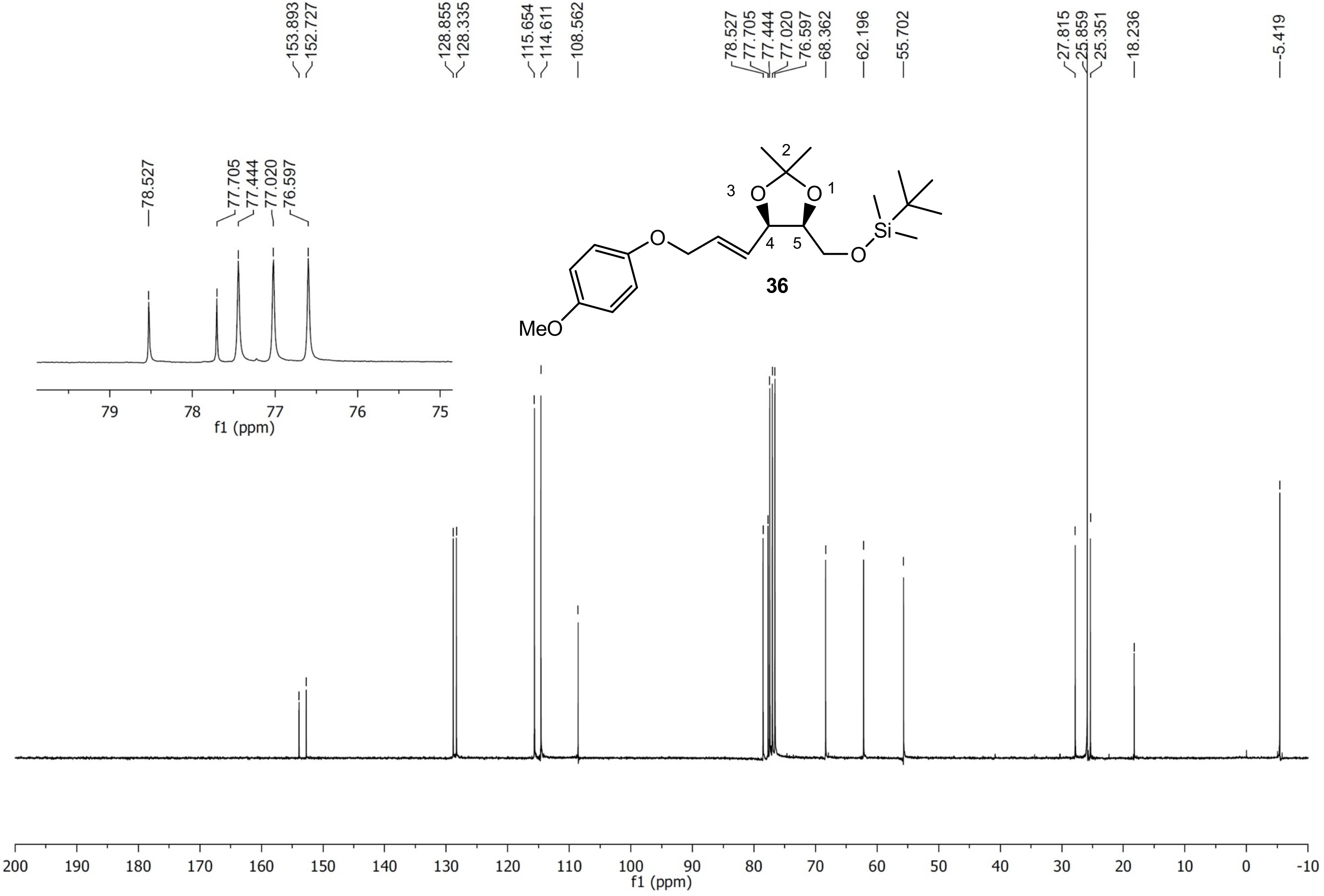
153.893
152.727
128.855
128.335
115.654
114.611
108.562
78.527
77.705
77.444
77.020
76.597
68.362
62.196
55.702
27.815
25.859
25.351
18.236
-5.419
MeO
O
Si
36
f1 (ppm)

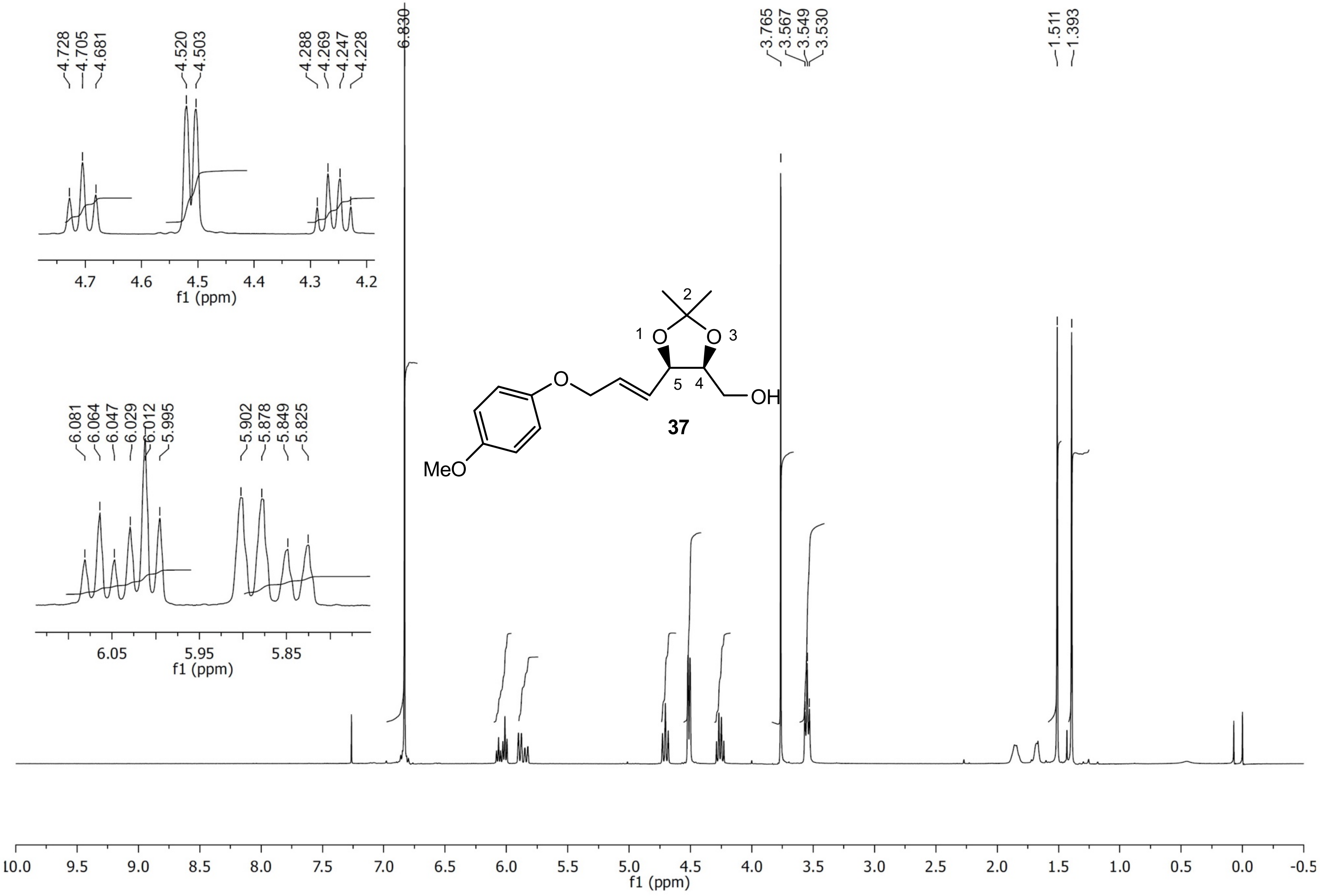
4.728
4.705
4.681
4.520
4.503
4.288
4.269
4.247
4.228
6.830
3.765
3.567
3.549
3.530
1.511
1.393
6.081
6.064
6.047
6.029
6.012
5.995
5.902
5.878
5.849
5.825
MeO
O
OH
1
2
3
4
5
37
f1 (ppm)

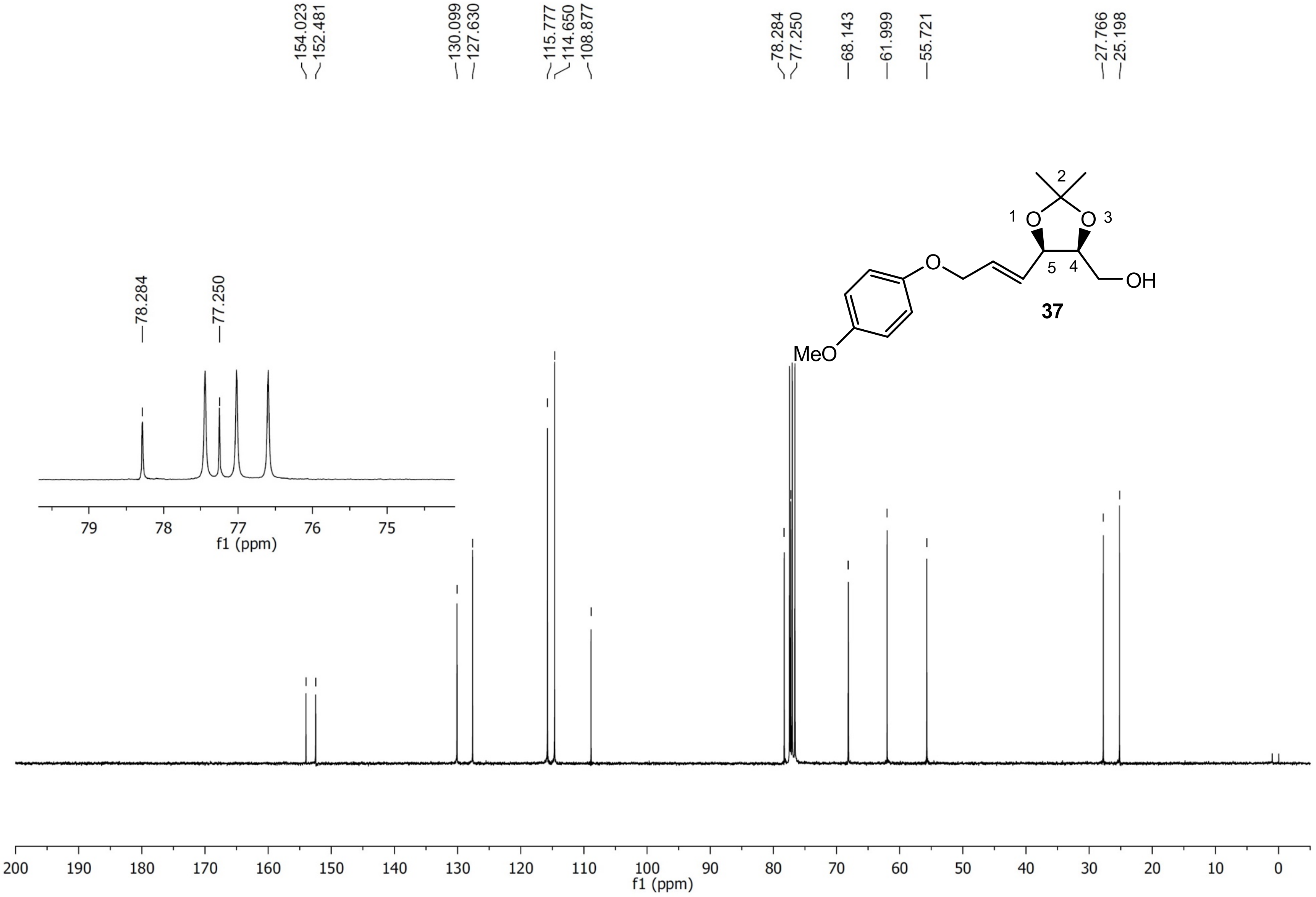
154.023
152.481
130.099
127.630
115.777
114.650
108.877
78.284
77.250
68.143
61.999
55.721
27.766
25.198
f1 (ppm)
37
MeO
OH

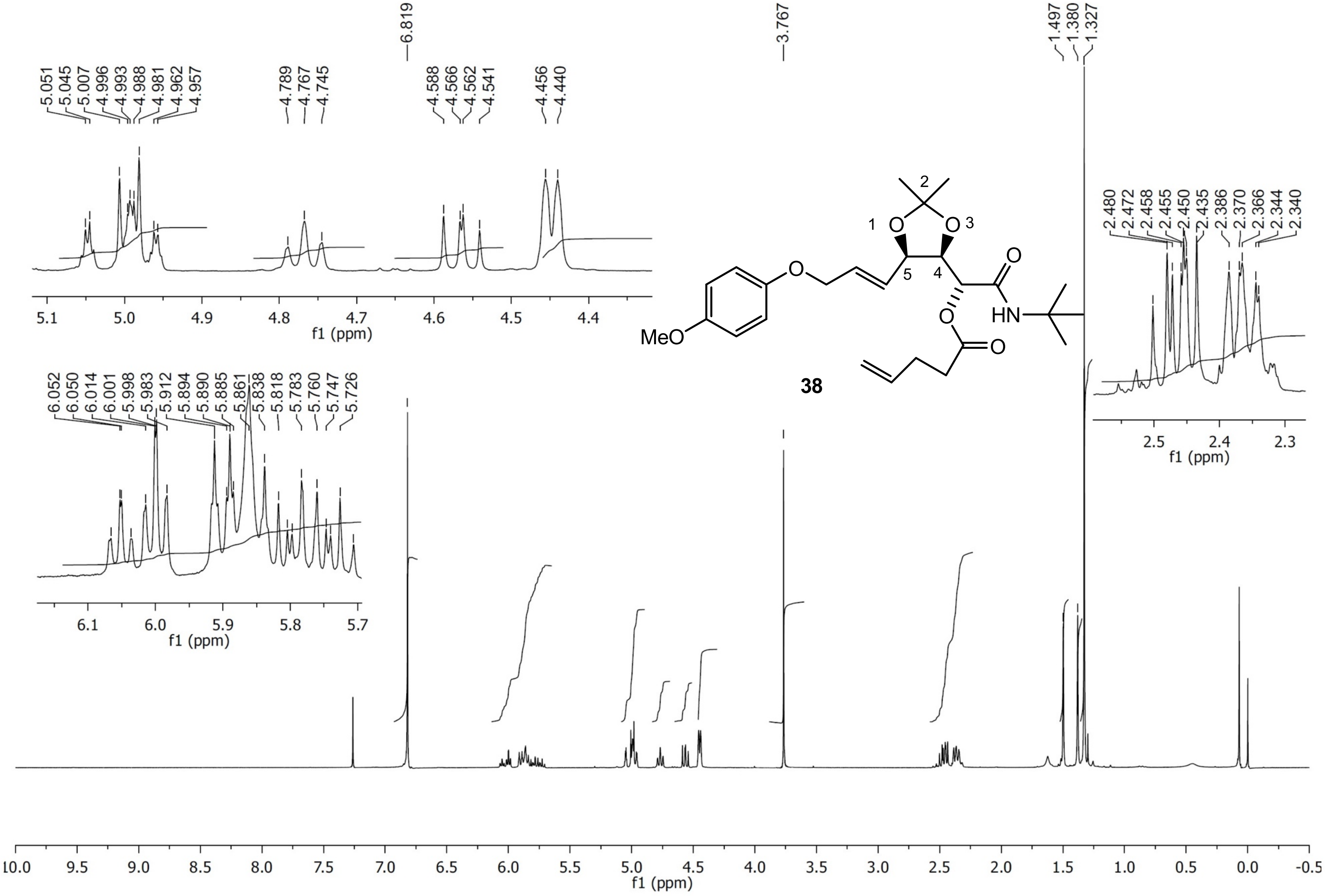

6.819
3.767
1.497
1.380
1.327
5.051
5.045
5.007
4.996
4.993
4.988
4.981
4.962
4.957
4.789
4.767
4.745
4.588
4.566
4.562
4.541
4.456
4.440
2.480
2.472
2.458
2.455
2.450
2.435
2.386
2.370
2.366
2.344
2.340
6.052
6.050
6.014
6.001
5.998
5.983
5.912
5.894
5.890
5.885
5.861
5.838
5.818
5.783
5.760
5.747
5.726
MeO
O
HN
38
f1 (ppm)

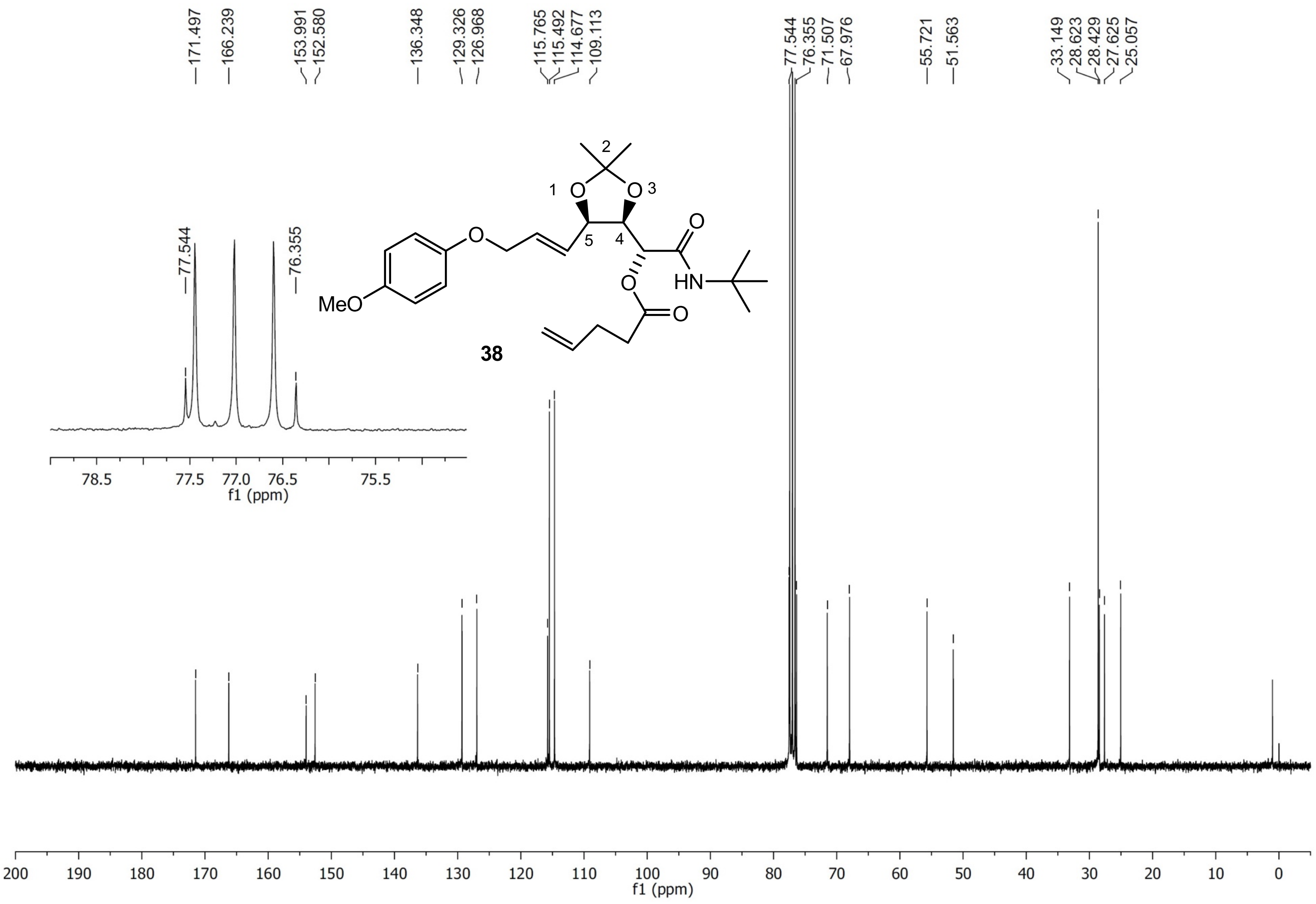
171.497
166.239
153.991
152.580
136.348
129.326
126.968
115.765
115.492
114.677
109.113
77.544
76.355
71.507
67.976
55.721
51.563
33.149
28.623
28.429
27.625
25.057
MeO
O
1
2
3
4
5
HN
38
77.544
76.355
78.5
77.5
77.0
76.5
75.5
f1 (ppm)
200
190
180
170
160
150
140
130
120
110
100
90
80
70
60
50
40
30
20
10
0
f1 (ppm)

4. Determination of diastereomeric ratios by ^{1}H-NMR

NMR of crude **20a** (entry 1 of Table 3). Ratio = 80 : 20

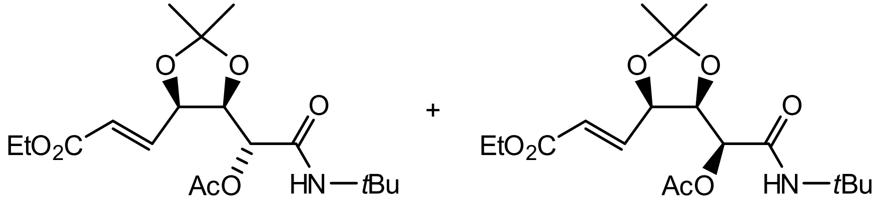

anti-**20a** *syn*-**20a**

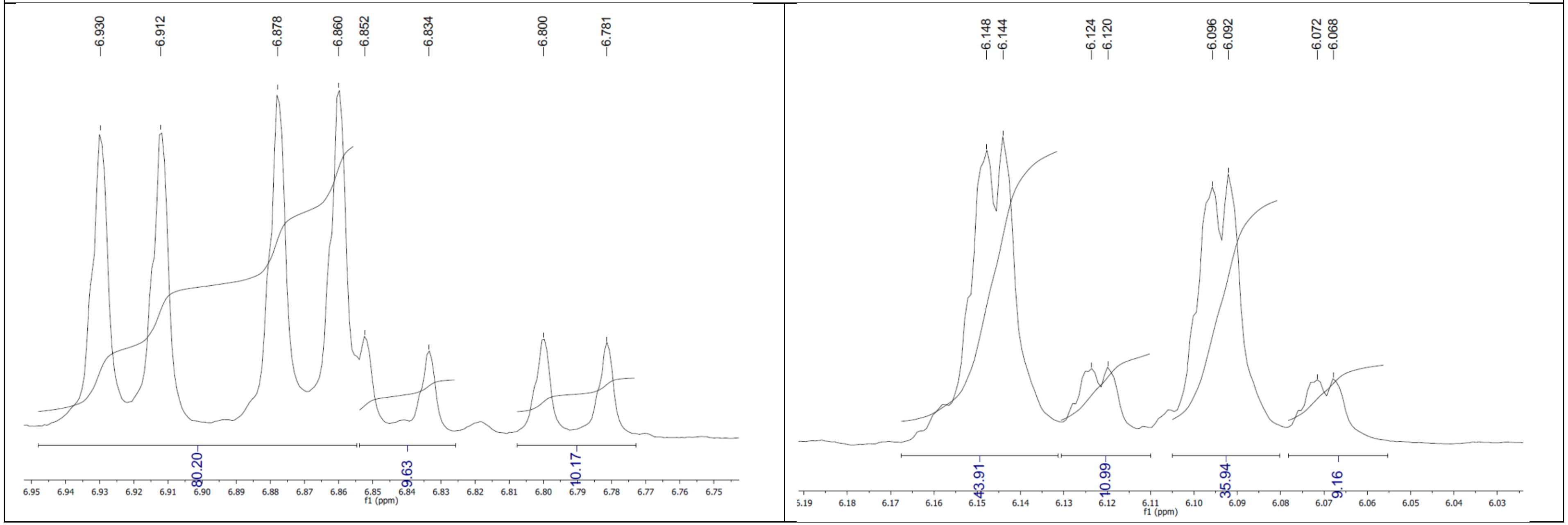

NMR of crude **20d** (entry 4 of Table 3). Ratio = 93 : 7

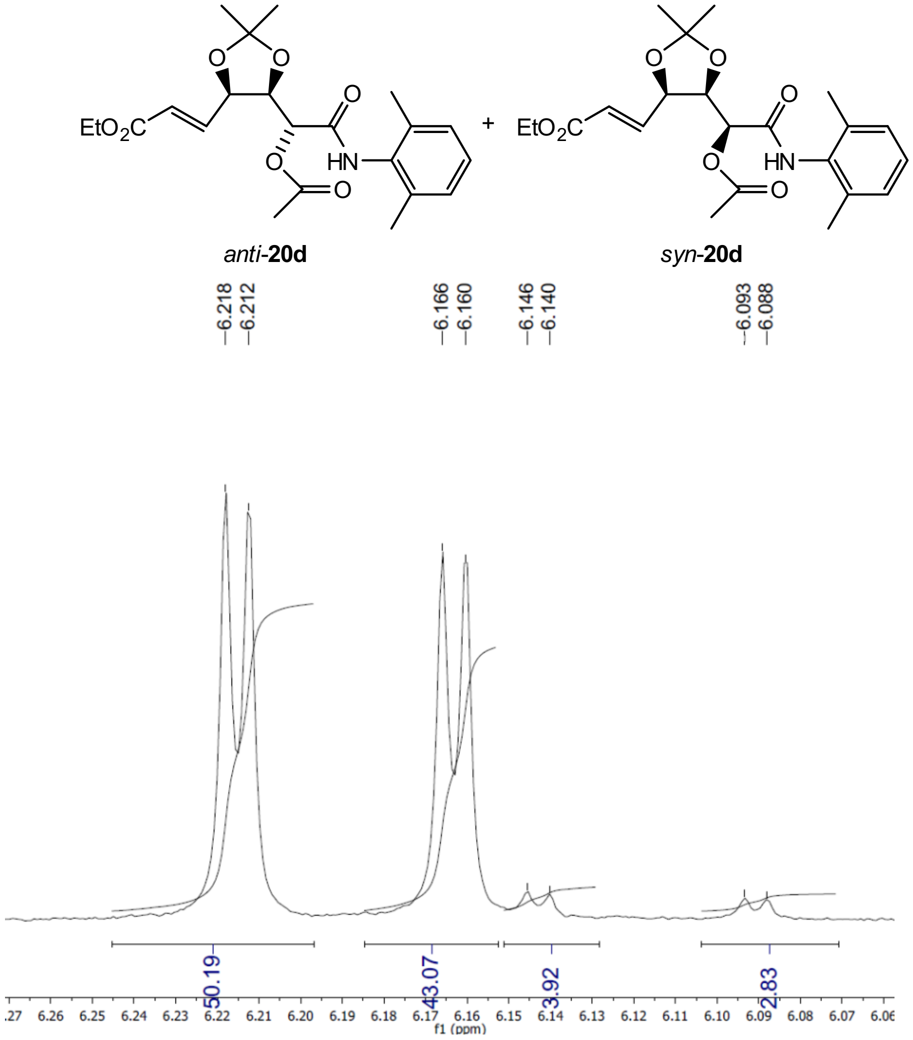

NMR of crude **20e** (entry 5 of Table 3). Ratio = 98 : 2

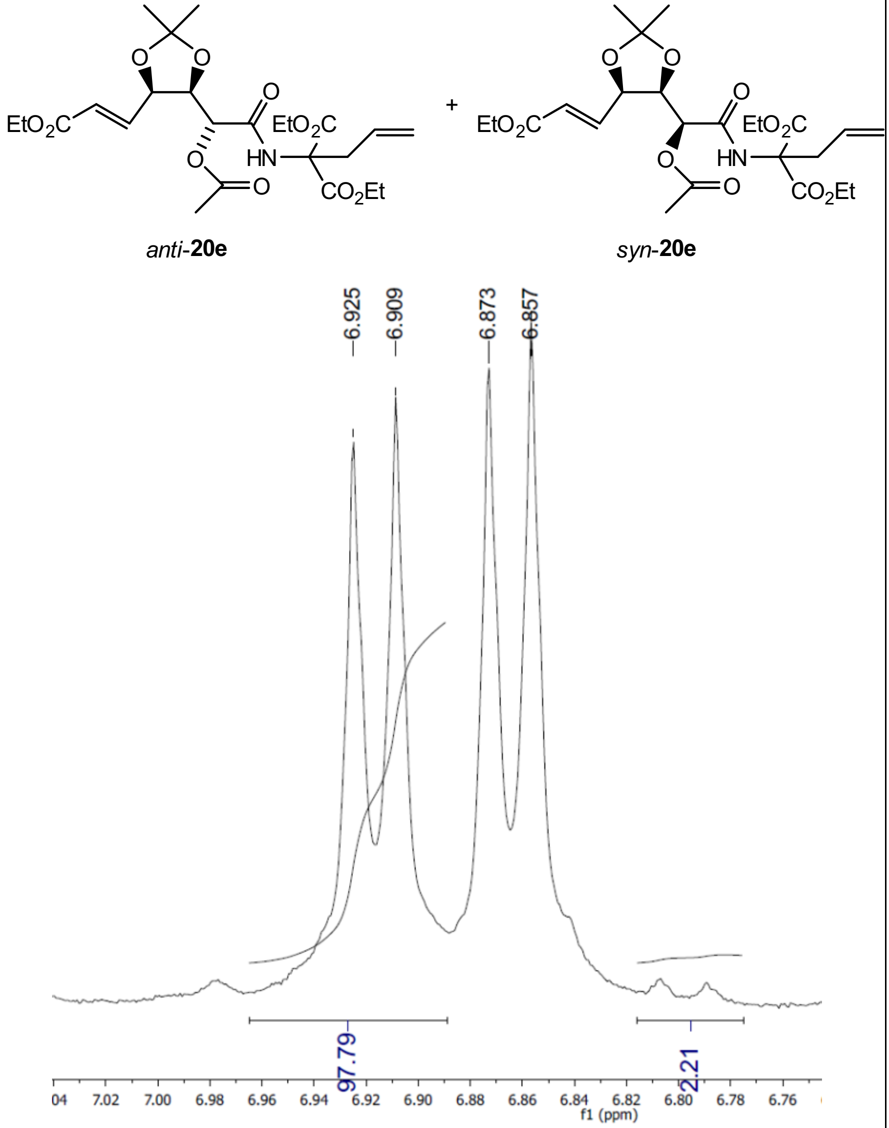

NMR of crude **20f** (entry 6 of Table 3). Ratio = 90 : 10

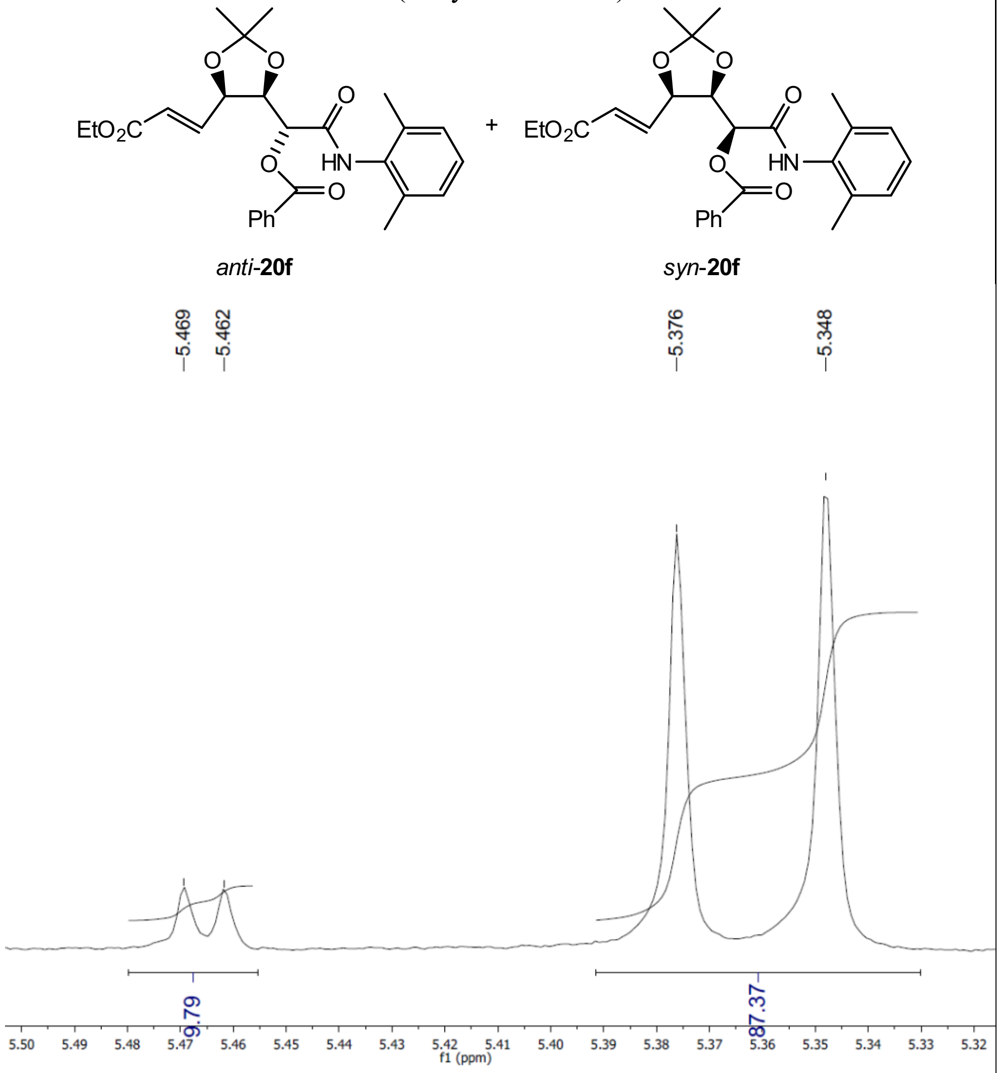

NMR of crude **20h** (entry 8 of Table 3). Ratio = 88 : 12

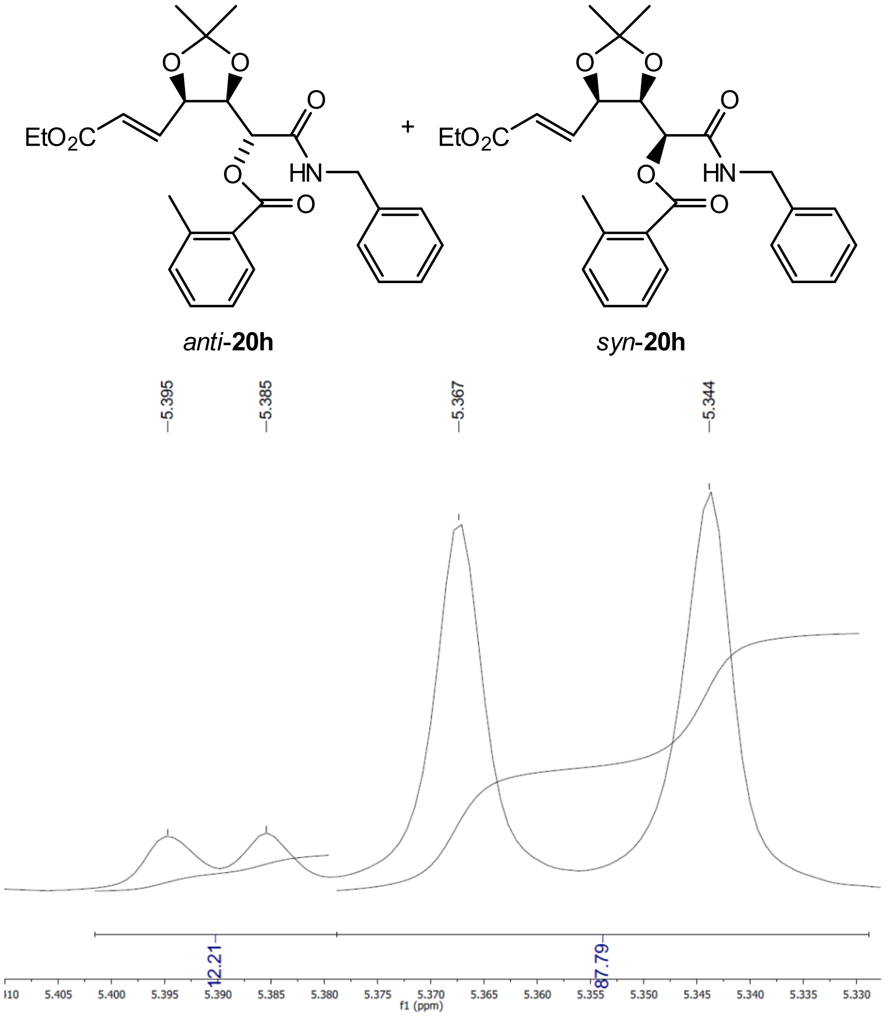

NMR of crude **20i** (entry 9 of Table 3). Ratio = 84 : 16

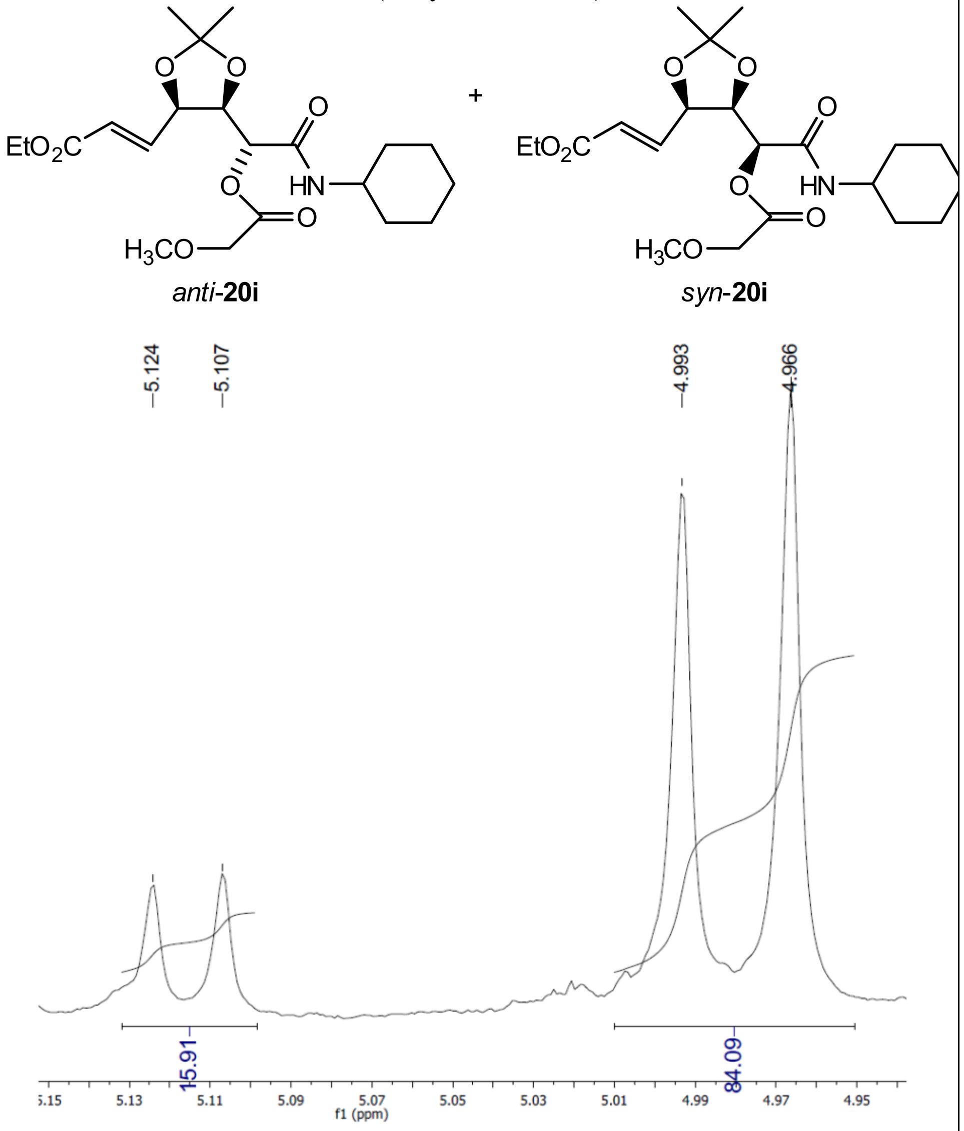

NMR of crude mixture of **24** and **25** (entry 3 of Table 4). Ratio = 92 : 8

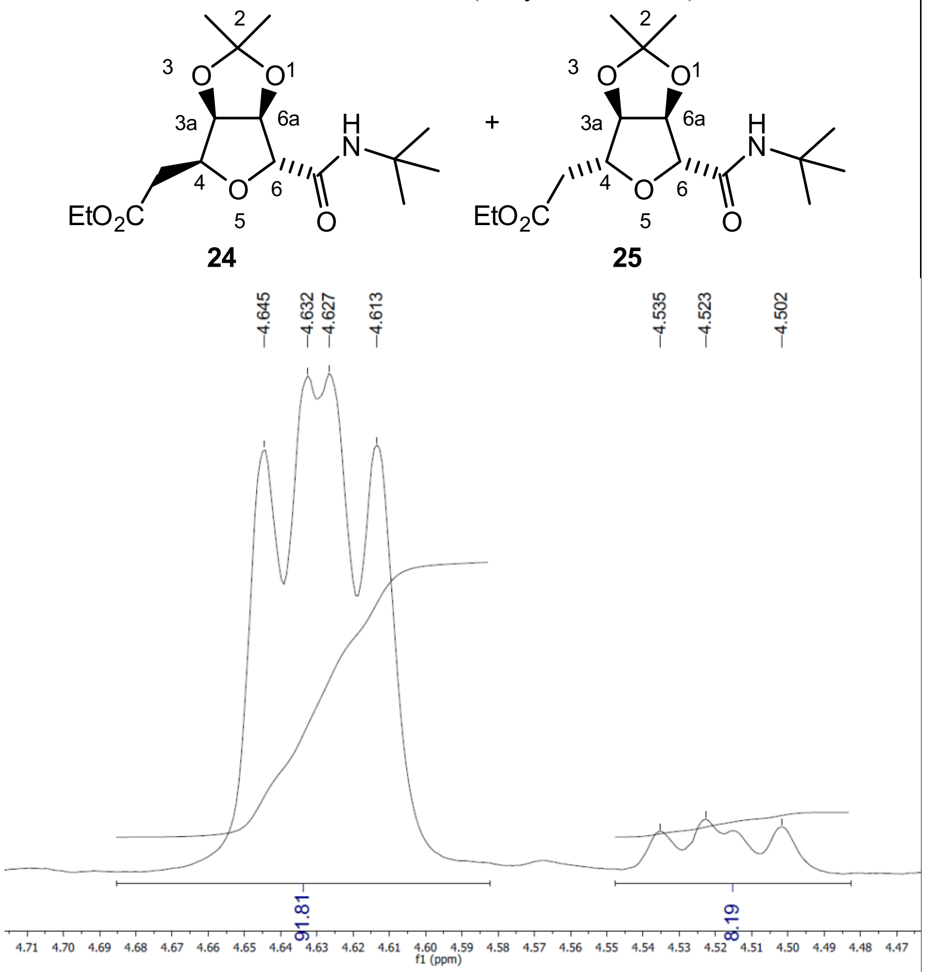

NMR of crude mixture of **24** and **25** (entry 3 of Table 4). Ratio = 92 : 8

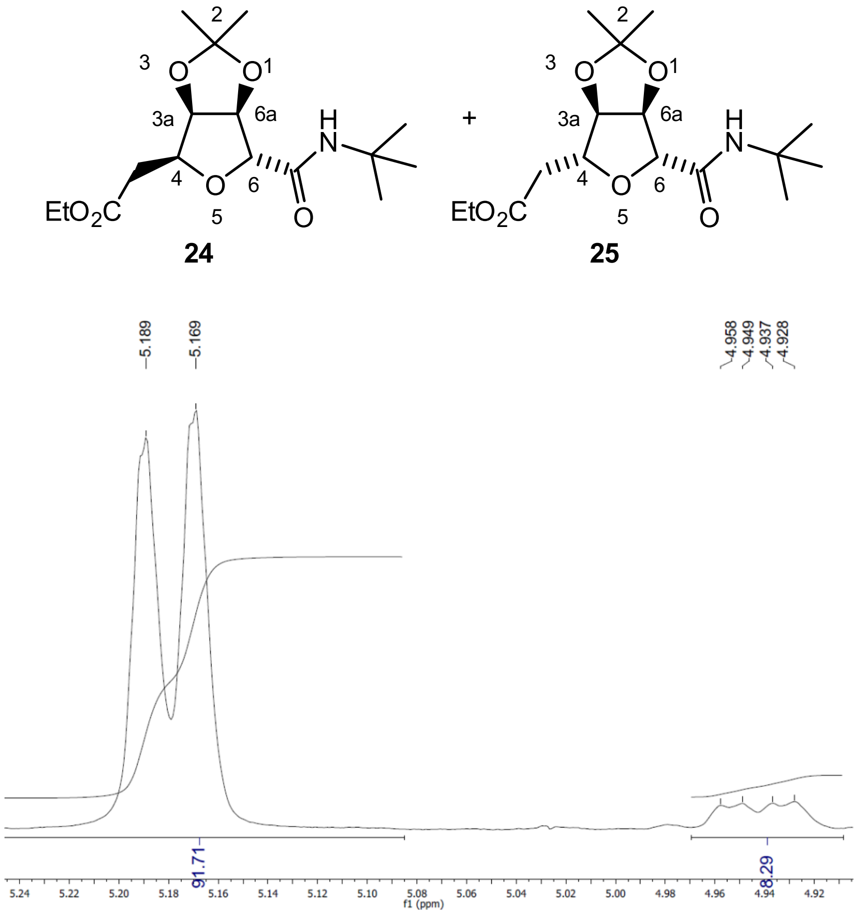

NMR of purified mixture of **26** and **27** (entry 8 of Table 4). Ratio = 82 : 18

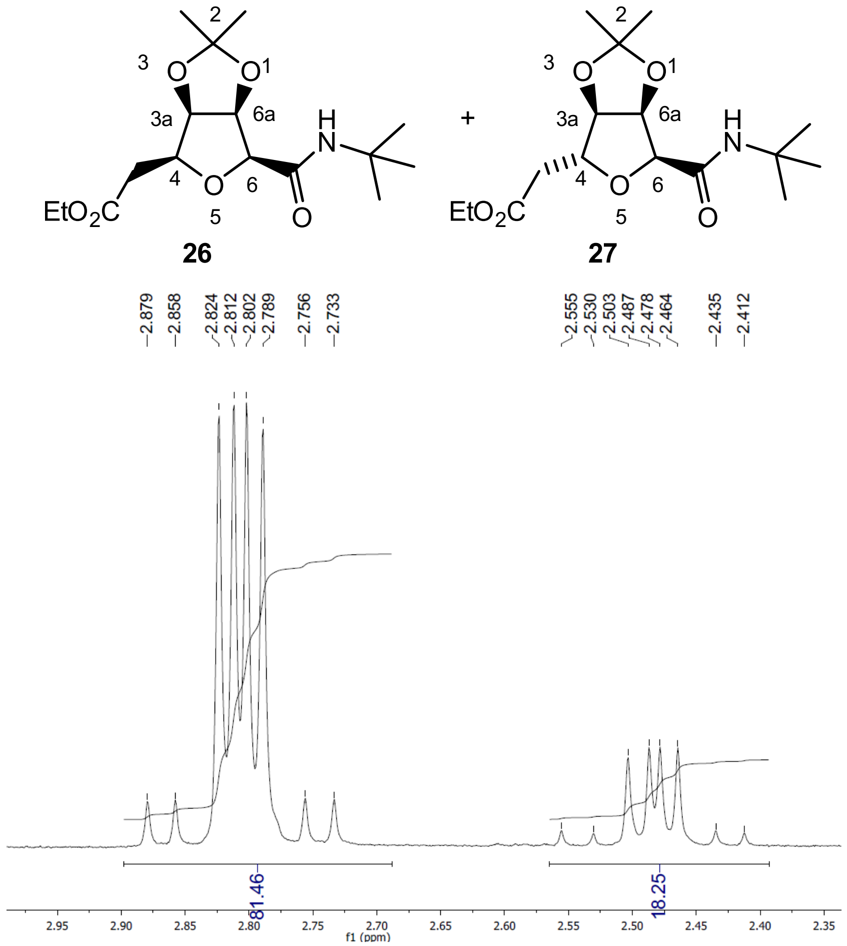

5. Determination of diastereomeric ratios by HPLC

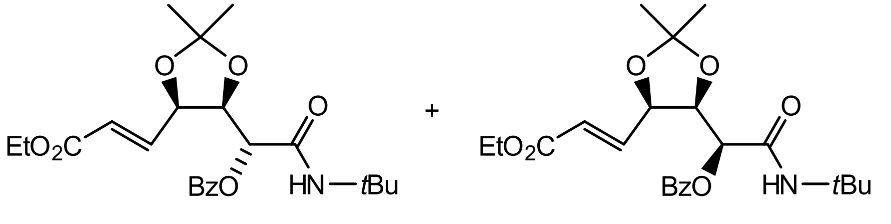

anti-**20b** *syn*-**20b**

(entry 2 of Table 3)

Column HYDRO RP 150 x 3 mm, 4 µ. Flow: 0.5 mL/min. Temp.: 25°C. Detection: UV, 220 nm. Eluent: H_2O / CH_3CN 50:50 (isocratic elution).

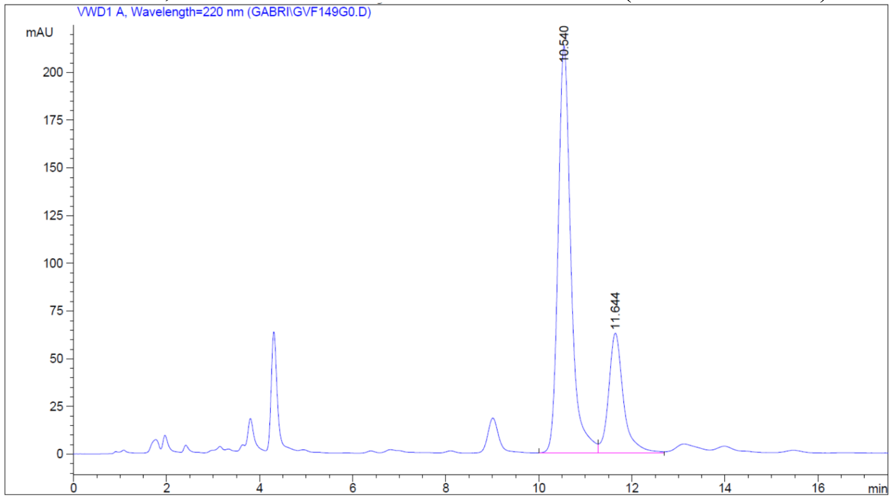

Peak #	RetTime [min]	Type	Width [min]	Area mAU *s	Height [mAU]	Area %
1	10.540	BV	0.2904	4089.25732	213.48854	74.5544
2	11.644	VV	0.3318	1395.67041	62.86977	25.4456

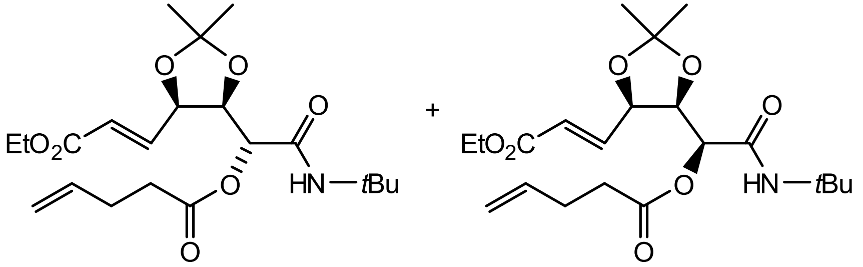

anti-**20c** *syn*-**20c**

(entry 3 of Table 3)

Column HYDRO RP 150 x 3 mm, 4 µ. Flow: 0.5 mL/min. Temp.: 25°C. Detection: UV, 210 nm. Eluent: H_2O / CH_3CN 50:50 (isocratic elution).

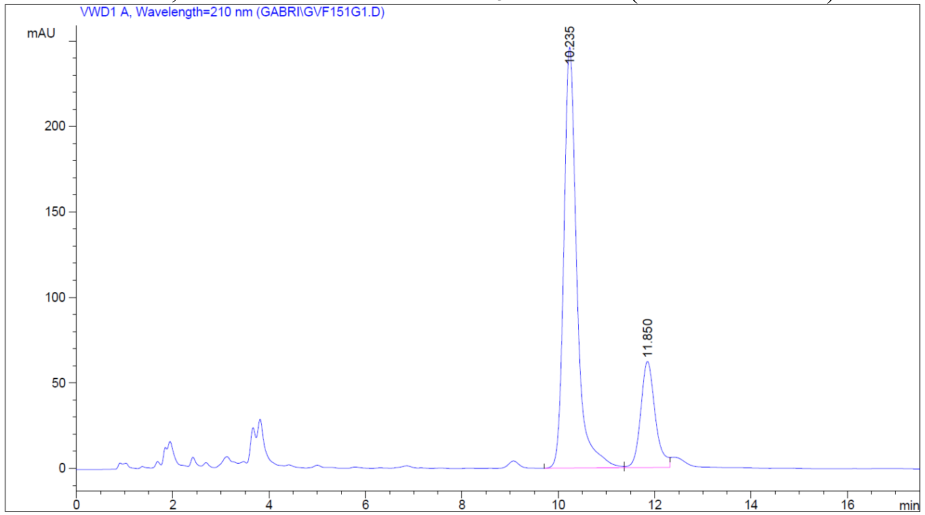

Peak #	RetTime [min]	Type	Width [min]	Area mAU *s	Height [mAU]	Area %
1	10.235	PV	0.2831	4624.19482	246.28583	78.1362
2	11.850	VV	0.3160	1293.92480	62.08185	21.8638

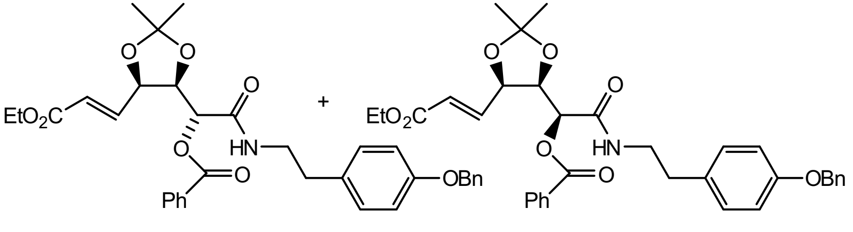

anti-**20g** *syn*-**20g**

(entry 7 of Table 3)

Column C6 PHENYLIC 150 x 3 mm, 3 µ. Flow: 0.34 mL/min. Temp.: 30°C. Detection: UV, 220 nm. Eluent: H_2O / MeOH 35:65 for 20 min, then up to 30 : 70 until min 25. Then H_2O : MeOH 30 : 70.

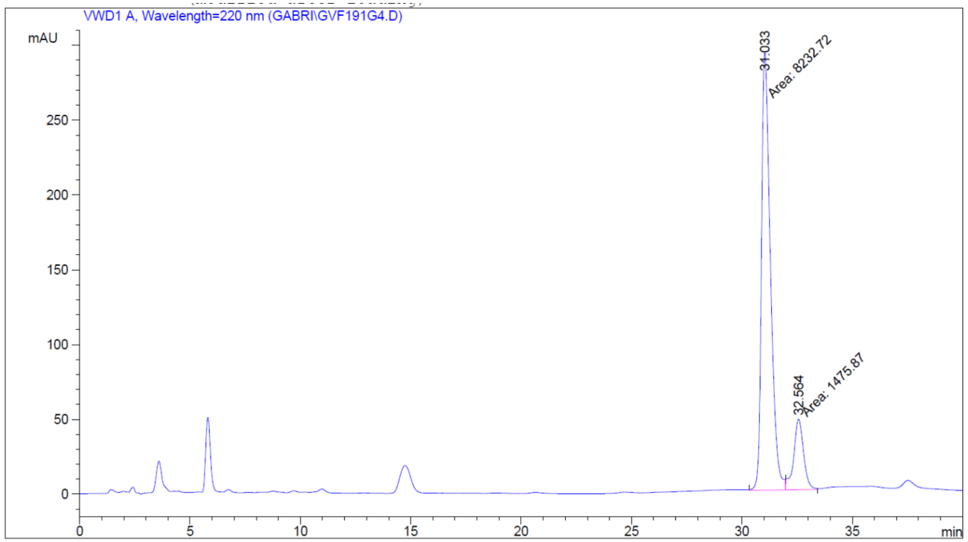

Peak #	RetTime [min]	Type	Width [min]	Area mAU *s	Height [mAU]	Area %
1	31.033	MF	0.4691	8232.72168	292.49667	84.7983
2	32.564	FM	0.5218	1475.86829	47.14125	15.2017
